# Antimicrobial peptide developed with machine learning sequence optimization targets drug resistant *Staphylococcus aureus* in mice

**DOI:** 10.1172/JCI185430

**Published:** 2025-04-22

**Authors:** Biswajit Mishra, Anindya Basu, Fadi Shehadeh, LewisOscar Felix, Sai Sundeep Kollala, Yashpal Singh Chhonker, Mandar T. Naik, Charilaos Dellis, Liyang Zhang, Narchonai Ganesan, Daryl J. Murry, Jianhua Gu, Michael B. Sherman, Frederick M. Ausubel, Paul P. Sotiriadis, Eleftherios Mylonakis

**Affiliations:** 1 Department of Medicine, Houston Methodist Hospital, Houston, Texas, USA.; 2 School of Pharmaceutical Sciences, and; 3 School of Biomolecular Engineering & Biotechnology, Rajiv Gandhi Technological University, Gandhinagar, Bhopal, Madhya Pradesh, India.; 4 Department of Electrical and Computer Engineering, National Technical University of Athens, Athens, Greece.; 5 Department of Pharmacy Practice and Science, College of Pharmacy, University of Nebraska Medical Center, Omaha, Nebraska, USA.; 6 Department of Molecular Biology, Cell Biology & Biochemistry, Brown University, Providence, Rhode Island, USA.; 7 Electron Microscopy Core, Houston Methodist Academic Institute, Houston, Texas, USA.; 8 Department of Biochemistry and Molecular Biology, Sealy Center for structural Biology and Molecular Biophysics, The University of Texas Medical Branch at Galveston, Galveston, Texas, USA.; 9 Department of Molecular Biology, Massachusetts General Hospital, Boston, Massachusetts, USA.; 10 Department of Genetics, Harvard Medical School, Boston, Massachusetts, USA.; 11 Archimedes - Athena Research Center, Marousi, Greece.

**Keywords:** Infectious disease, Microbiology, Bacterial infections

## Abstract

As antimicrobial resistance rises, new antibacterial candidates are urgently needed. Using sequence space information from over 14,743 functional antimicrobial peptides (AMPs), we improved the antimicrobial properties of citropin 1.1, an AMP with weak antimethicillin resistant *Staphylococcus aureus* (MRSA) activity, producing a short and potent antistaphylococcal peptide, CIT-8 (13 residues). At 40 μg/mL, CIT-8 eradicated 1 × 10^8^ drug-resistant MRSA and vancomycin resistant *S*. *aureus* (VRSA) persister cells within 30 minutes of exposure and reduced the number of viable biofilm cells of MRSA and VRSA by 3 log_10_ and 4 log_10_ in established biofilms, respectively. CIT-8 (at 32 μg/mL) depolarized and permeated the *S*. *aureus* MW2 membrane. In a mouse model of MRSA skin infection, CIT-8 (2% w/w in petroleum jelly) significantly reduced the bacterial burden by 2.3 log_10_ (*P* < 0.0001). Our methodology accelerated AMP design by combining traditional peptide design strategies, such as truncation, substitution, and structure-guided alteration, with machine learning–backed sequence optimization.

## Abstract 2

Machine learning assisted antimicrobial peptide development against drug-resistant pathogens.

## Introduction

The World Health Organization (WHO) identifies antimicrobial resistance (AMR) as one of the biggest threats to global health, leading to more hospitalized patients, longer hospital stays, higher medical costs, and increased mortality (1). With the rapid rise in antimicrobial resistance to clinically available antibiotics, novel antibiotic candidates are urgently needed (2). Antimicrobial peptides (AMPs) are promising candidates to develop into new clinically relevant antibiotics because they kill drug-resistant pathogens, modulate host immune responses, and are less likely to induce antimicrobial resistance (3). Several modified AMPs, such as daptomycin (lipopeptide), bacitracin (cyclic peptide), and gramicidin S (cyclic peptide), are currently in clinical use, suggesting that more AMPs could be identified or designed and developed to combat drug resistance (4).

AMPs can be designed using noncomputational (traditional) or computational methods (5). The traditional methods of AMP design involve optimizing a single or a limited number of peptide templates by truncation (6), substitution (7), sequence shuffling (8), motif hybridization (9), structure-based approaches (10), and/or de novo combinatorial library-based strategies (11). These optimization processes often rely on evolutionary information concerning natural AMPs, such as the use of frequently occurring amino acids (12), motifs (13), or quantitative structure-activity–relationship (QSAR) models (14). A traditional approach to AMP design preserves the original template properties in terms of activity, selectivity, and stability (15, 16). However, it limits sequence optimization because it does not “test” all potentially favorable combinations of amino acids that represent a broad combinatorial sequence space corresponding to all known functional AMPs.

In contrast to traditional methods of AMP design, computational methods utilize extensive sequence-space information from dedicated AMP databases to compute physicochemical parameters or identify sets of potentially active amino acid substitutions using pattern recognition methodologies. Computational methods include in silico approaches based on a linguistic grammar-based model (17) and ab initio approaches based on a database filtering technology (13). More recently, the use of machine learning (ML) in antibacterial drug discovery has been shown to be a promising computational drug design methodology (18–20). However, AMP design strategies based on ML are mostly associated with developing neural networks and deep learning models based on genomic and proteomic sources (20, 21). Despite the ability of ML algorithms to process large data sets, their use has been essentially limited to identification and prediction of AMPs (22–24) rather than the generation of novel AMPs. There are only a few attempts that have employed machine learning to design and optimize AMPs, such as in the design of temporin AMPs from frogs (19) and the design of lipopolysaccharide-binding peptides (LBD_B_) (25) by leveraging genetic algorithms combined with fitness matrix-based amino acid substitutions.

In this study, we combined traditional and ML-based AMP design strategies to preserve the original template-dependent properties typical of traditional designs while at the same time carrying out sequence optimization powered by ML. We sought to demonstrate that our approach is highly efficacious for improving AMPs. We used peptide sequence–space information from publicly available AMP databases, employed k-means clustering that incorporated physicochemical parameters regulating AMP properties (26), and derived knowledge graphs to identify the most preferred amino acid occurrences. We implemented our peptide design strategy on citropin 1.1, a natural AMP with modest activity against methicillin-resistant *Staphylococcus aureus* (MRSA), with the goal of developing a more potent and shorter derivative AMP that is active against MRSA. We describe the strategy used to optimize the anti-MRSA properties of short citropin1.1 peptides and report the antistaphylococcal efficacy of a specific engineered derivative, CIT-8, in vitro and in vivo in a topical skin infection model. We also describe the cytotoxic properties of CIT-8 as well as its mechanism of action. In addition, 3 additional AMPs were also modified to substantially enhance their activity against MRSA, employing the same protocol used to design CIT-8, thereby demonstrating the generalizability of our design methodology. Our findings highlight the potential of ML in accelerating the development of potent AMPs while reducing the time and cost associated with traditional combinatorial approaches. By integrating ML with targeted sequence modifications, our approach provides a scalable and efficient framework for designing potent AMPs to combat antibiotic-resistant pathogens.

## Results

### Peptide design and optimization based on traditional AMP design principles.

**Figure 1:**
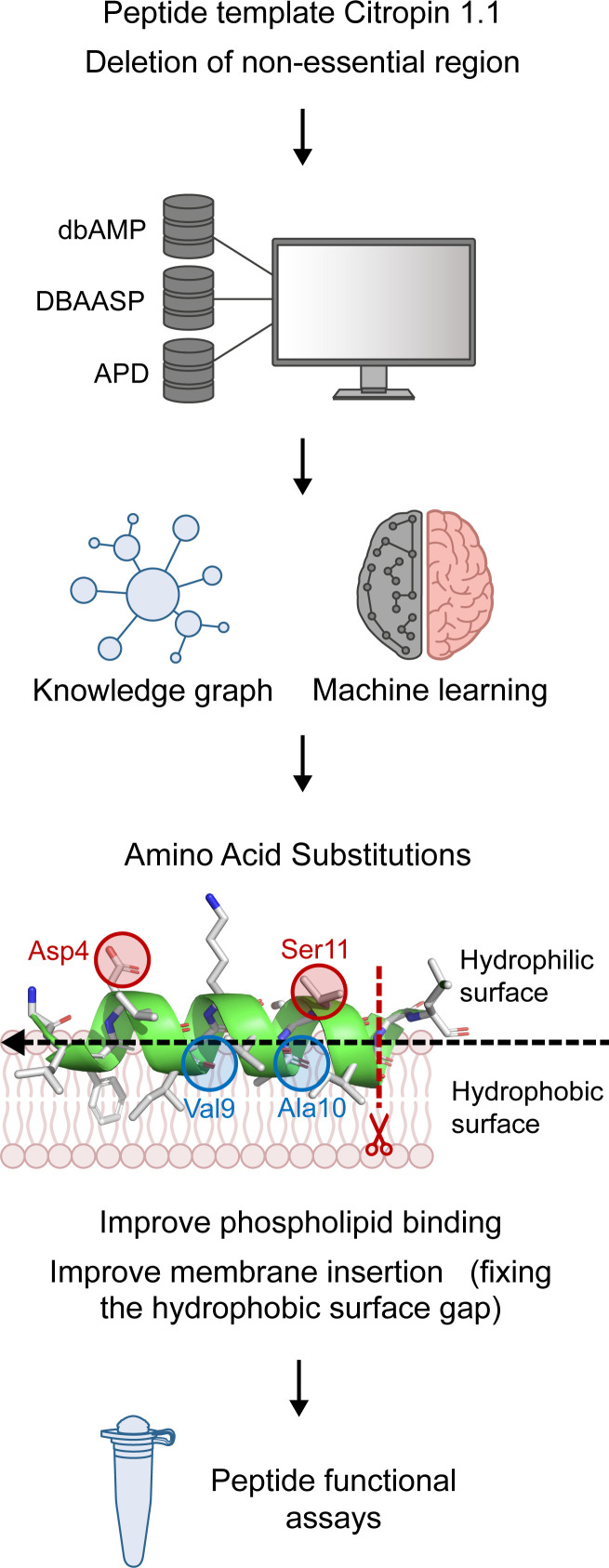
CIT-based peptide design and optimization strategy.

We hypothesized that short and potent antistaphylococcal AMPs could be designed by modifying nonactive or weakly active AMP templates using a combination of traditional AMP design strategies with machine learning guidance. Our goal was to enhance the antimicrobial efficacy of AMPs (denoted as templates) by producing shorter and more potent derivatives using our approach. To test this hypothesis, we selected the citropin 1.1 template because of its modest efficacy against MRSA, aiming to sequentially perform truncation, structure-guided alteration, and substitution assisted by ML to enhance the interaction of the peptide with the bacterial membrane to create highly potent AMP candidates (Figure 1).

Citropin 1.1 is a natural 16-amino-acid–long cationic (+2) and helical AMP secreted by *Litoria citropa*, an Australian blue mountains tree frog (Swiss Prot ID; P81835). First, we sought to shorten the peptide and improve its stoichiometric interaction with the bacterial membrane. We optimized the α-helix of citropin 1.1 by removing the nonessential helix-breaking C-terminus, which is comprised of a nonamphipathic extension containing two glycine residues and one leucine residue (27). The resultant peptide, CIT-1, had 13 amino acid residues with 3 helical turns. Structurally, CIT-1 displayed a negatively charged aspartic acid residue at the fourth position and a neutral serine residue at the eleventh position on its hydrophilic face. On its hydrophobic face, it had a distinct nonhomogeneous surface gap lacking larger hydrophobic amino acids at positions 9 and 10, which were occupied by valine and alanine, respectively (Supplemental Figure 1; supplemental material available online with this article; https://doi.org/10.1172/JCI185430DS1).

As the initial AMP-membrane interaction is a charge-charge driven one (3), we postulated that substituting the fourth aspartic acid and the eleventh serine positions of CIT-1 with positively charged residues would facilitate interaction with the bacterial membrane’s negatively charged phospholipid head groups. To enhance the membrane insertion capability of the peptide, we also reasoned that valine 9 and alanine 10 could be replaced with bulkier hydrophobic amino acid residues to ensure more effective interaction between the peptide-hydrophobic amino acids and the hydrophobic chains of the bacterial membrane phospholipids.

### Identification of the optimal amino acid substitutions using ML and knowledge graphs.

After using traditional AMP design principles as described in the previous section to shorten citropin 1.1 to CIT-1 and to identify potential amino acid positions for amino acid substitutions to improve its anti-MRSA properties, we employed machine learning to determine which amino acids would be most suitable to replace the original ones at positions 4, 9, 10, and 11. To train the ML algorithm, we created an aggregated AMP dataset that consisted of 14,743 unique peptide sequences with AMP properties after preprocessing (access date: February 9, 2024) and calculated the molecular weight, the 5-dimensional PCP descriptors, GRAVY, hydrophobic moment, helicity, and topological surface area (TPSA), which are used as a quantitative means to identify property motifs in sequences of protein families. The peptides included in the dataset had molecular weights ranging from 2,637.71 to 58,471.10 g/mol, GRAVY scores between –4.5 and 4.5, mean helicity of 0.36 (SD: 0.22) and mean hydrophobic moment of 0.58 (SD: 0.22) (Supplemental Table 1).

**Figure 2:**
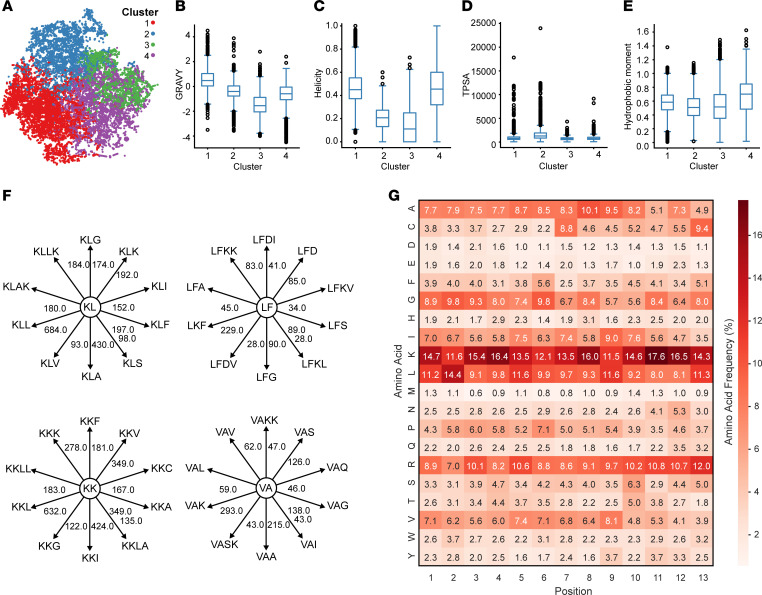
Exploration of AMP sequence space. (**A**) t-SNE transformed physicochemical property space, grouped by k-means clustering. (**B**–**E**) Comparison of the physicochemical parameters of the 4 clusters of AMPs in our dataset based on (**B**) GRAVY, (**C**) helicity, (**D**) TPSA, and (**E**) hydrophobic moment. (**F**) Graph of amino acid group occurrence patterns (AGO). Each node represents a peptide fragment sequence, while the connected nodes indicate the most common parental fragment sequences with corresponding NOO (number of occurrences) values annotated on the edges. (**G**) Heat-map showing the percentage of each amino acid (APO) in all 13-mer helical AMP fragments in our dataset.

The dataset we created consisted of peptides from various prokaryotic and eukaryotic sources, which exhibited varied antimicrobial activity against several Gram-positive and Gram-negative bacteria and displayed diverse secondary structures including α-helix, β-sheets, α + β, and non-αβ peptides. To select the most appropriate group of peptides to use to identify potential substitutions at positions 4, 9, 10, and 11, we used the k-means method to cluster the dataset into groups that exhibited distinct physicochemical characteristics (Figure 2A). A silhouette analysis of the dataset showed that four clusters provided the most well-defined and distinct grouping of peptides, meaning that the peptides within each cluster were more similar to each other while being more different from those in other clusters. Cluster 1 included peptides predominantly characterized by their high GRAVY (mean: 0.53, SD: 0.73) and helicity (mean: 0.48, SD: 0.17) and low TPSA (Figure 2, B–E, and Supplemental Table 2). Cluster 2 contained peptides that had lower hydrophobicity, helicity and hydrophobic moment compared to cluster 1, but high TPSA. Peptides in Cluster 3 had even lower GRAVY, helicity, and hydrophobic moment compared with cluster 1, but shared comparable TPSA. Finally, Cluster 4 was comprised of peptides with lower GRAVY, similar helicity and TPSA, and higher hydrophobic moment compared to cluster 1 peptides.

As we were interested in designing antistaphylococcal helical peptides and recognized that AMPs with high GRAVY, helicity, and an extended hydrophobic surface are characteristic features of potent helical antistaphylococcal peptides (13), we selected Cluster 1 to obtain the amino acid patterns that would guide the substitutions at positions 4, 9, 10, and 11 in CIT-1. Our supposition was that, by taking into consideration the pattern of the neighboring amino acids at these positions, we could identify the optimal evolutionarily favored substitutions. We defined this pattern of a small group (2–4 amino acid residues) of selective amino acids cooccurring in an AMP sequence as “amino acid group occurrence” (AGO), which served as the foundation for our substitutions in the CIT-1 template. We constructed a comprehensive knowledge graph to identify the AGO patterns in the peptide sequences of Cluster 1 peptides. This graph connected fragments based on sequence containment, where a parent fragment contained the child fragment within its sequence, allowing for a detailed analysis of peptide structure and relationships within the cluster.

For position 4, the knowledge graph query for the parent fragments (2 or 3 cooccurring amino acid residues) of ‘leucine-phenylalanine’ (positions 2–3) (denoted as ‘LF’) showed a preferential substitution of D (aspartate (present in CIT-1) with ‘K.’ This substitution was based on its recurrence in parent fragments, with ‘LFK’ having the highest AGO instances (number of occurrences [NOO] = 229) followed by LFG (NOO = 90) and then LFS (NOO = 89) (Figure 2F). Next, we replaced the serine at eleventh position with lysine, as the corresponding AGO instances for parent fragment ‘VA’ (position 9–10) were ‘VAK’ (NOO = 293), which was greater than that for the native sequence ‘VAS’ (NOO = 126).

For positions 9 and 10, we used 2 consecutive leucine residues to fill the hydrophobic gap, as guided by the AGO patterns (Figure 2F). More specifically, for position 9, the parent sequence ‘KK’ (position 7–8) was followed by ‘L’ with ‘KKL’ being the most prevalent AGO instance (NOO = 632) compared to ‘KKI’ (NOO = 424), ‘KKA’ (NOO = 349) and ‘KKV’ (NOO = 349). Similarly, the second leucine (position 10) was justified by the AGO patterns of the parent fragment ‘KL’ (position 8–9) with prevalent ‘KLL’ AGO with NOO = 684 compared to ‘KLA’ (NOO = 430), ‘KLF’ (NOO = 197) and ‘KLK’ (NOO = 192).

Finally, we sequentially substituted each amino acid at position 4, 9, 10, and 11 on CIT-1 in a stepwise manner to generate 8 peptide candidates with different charge-to-hydrophobicity contents. These peptides included CIT-1 (contains the original negatively charged aspartic acid residue at fourth position), CIT-2 (contains a neutral serine residue at fourth position), CIT-3 (included a charged lysine residue at fourth position), CIT-4 (with lysine residues at fourth and eleventh positions), CIT-5 (CIT-1 + hydrophobicity improved at positions 9–10), CIT-6 (CIT-2 + hydrophobicity improved at positions 9–10), CIT-7 (CIT-3 + hydrophobicity improved at positions 9–10) and CIT-8 (CIT-4 + hydrophobicity improved at positions 9–10) (Supplemental Figure 2). Peptides CIT-1 and CIT-8 contained the minimum and maximum AGO instances, respectively.

### Validation of the choice of amino acids for substitutions.

To verify that the amino acid substitutions in CIT-1 based on AGO patterns are evolutionary conserved, we examined the amino acid occurrences in all 13-mer helical peptides in our dataset. We calculated the most helical 13-mer sequence fragment for each peptide and generated a heatmap from the entire dataset, displaying amino acid frequencies at each position. This allowed us to compare and contrast the structural nuances of the CIT peptides, specifically at the targeted positions 4, 9, 10, and 11 (Figure 2G). The heatmap revealed that pronounced amino acid percent occurrence (APO) frequencies are similar to AGO patterns directed substitutions. At position 4, lysine occurred at 16.4% compared with 8.2% for arginine. At position 9, leucine appeared at 11.6%, followed by isoleucine at 9%, phenylalanine at 3.5%, and tryptophan at 2.3%. At position 10, leucine occurred at 9.2%, compared with 8.2% for alanine and 7.6% for isoleucine. At position 11, lysine appeared at 17.6%, compared with 10.8% for arginine (Figure 2G).

### Antimicrobial potency and cytotoxicity.

We conducted standard minimum inhibitory concentration (MIC) assays on peptides CIT-1 through CIT-8 to check the antimicrobial potencies of the CIT-derived peptides. Peptides CIT-1 to CIT-5 had MICs greater than 32 μg/mL and were ineffective in killing *S*. *aureus* strain MW2, despite having net charges ranging from + 2 to + 5 and 61% hydrophobicity to bind the bacterial membrane effectively (Table 1). In contrast, CIT-6, CIT-7, and CIT-8 had MICs of 2–4 μg/mL (Table 1). These 3 peptides had either neutral or positive substitutions at position 4 and improved hydrophobicity by filling hydrophobic structural gaps at positions 9–10. The helical wheel plot showed that peptides CIT-6, CIT-7, and CIT-8, which were the most antimicrobial, had well-distributed hydropathy (Supplemental Figure 3).

To measure toxicity, we tested the hemolytic potential of CIT-derived peptides against human RBCs, calculating the concentration of peptide that induced 50% hemolysis (HL_50_) (Supplemental Figure 4A). Compared with CIT-6 and CIT-7, CIT-8 had the lowest HL_50_ value of 68 μg/mL, 17 times greater than its MIC. In addition, we tested the cytotoxicity potential of CIT-8 against human liver–derived HEPG2 cells. The concentration of CIT-8 needed to kill 50% of HEPG2 cells (LD_50_) was 96 μg/mL, indicating a large therapeutic window (Supplemental Figure 4B).

### Design of additional AMPs.

To demonstrate that our machine learning–based peptide design approach is not limited to CIT-8, we generated additional peptides using three randomly selected new templates with weak anti-MRSA efficacy: hylaseptin P1, mastoparan-L, and r-CAMEL. The ML-designed peptides derived from these templates using our AMP design strategy (described in Supplemental Table 3) exhibited substantially improved MIC values (MIC = 2 μg/mL) against MRSA compared with their respective WT peptide sequences (MIC greater than 32 μg/mL) (Supplemental Table 3), suggesting that our protocol for designing AMPs is generalizable and not limited to CIT-8.

Furthermore, unlike traditional AMP design methods that involve limited sequence modifications, our hybrid approach, combining ML with traditional peptide design, enables a single-step sequence optimization, efficiently transforming weakly active templates into highly potent and shorter AMPs. By leveraging traditional analysis, we first shortened the template peptide and identified key amino acid positions for modification, an essential step that ML alone could not achieve. In parallel, ML-driven analysis eliminated the need for exhaustive combinatorial synthesis, which would have traditionally required testing over 160,000 peptide variants from a 13-mer AMP template by substituting four positions with all 20 amino acids (20^4^ = 160,000). Even with a more constrained approach, limiting substitutions to two charged residues (lysine or arginine) and 6 key nonpolar hydrophobic residues (leucine, phenylalanine, isoleucine, tryptophan, proline, and methionine), at least 144 variants would still need to be screened (that is, test 2 positions with 2 amino acids and 2 positions with 6 amino acids; 2^2^ × 6^2^ = 144). These findings underscore the advantage of integrating ML with traditional AMP design, providing a rapid, cost-effective, and targeted strategy for developing potent antimicrobial agents against resistant pathogens compared with conventional peptide design methods.

### Antimicrobial robustness of CIT-8 peptide.

Once we determined that CIT-8 had the lowest hemolytic activity among the selected peptides, we investigated its potential as a potent antistaphylococcal agent. We tested the robustness of its antibacterial activity in the presence of physiological salts and serum. The MIC of CIT-8 remained the same (4 μg/mL) even in the presence of 150 mM NaCl, 2.5 mM CaCl_2_, 8 mM ZnSO_4_, or 1 mM MgSO_4_. The presence of 5%–10% human serum increased the MIC by only 2-fold (Table 2).

To further characterize the antimicrobial potency of CIT-8, we tested it against various drug-resistant *S*. *aureus* strains. The MIC of CIT-8 was 4-8 μg/mL against various VRSA, MRSA, VISA, and *S*. *aureus* clinical isolates (Table 3).

### Antimicrobial potency of CIT-8 against drug-resistant S. aureuspersister cells and biofilms.

**Figure 3:**
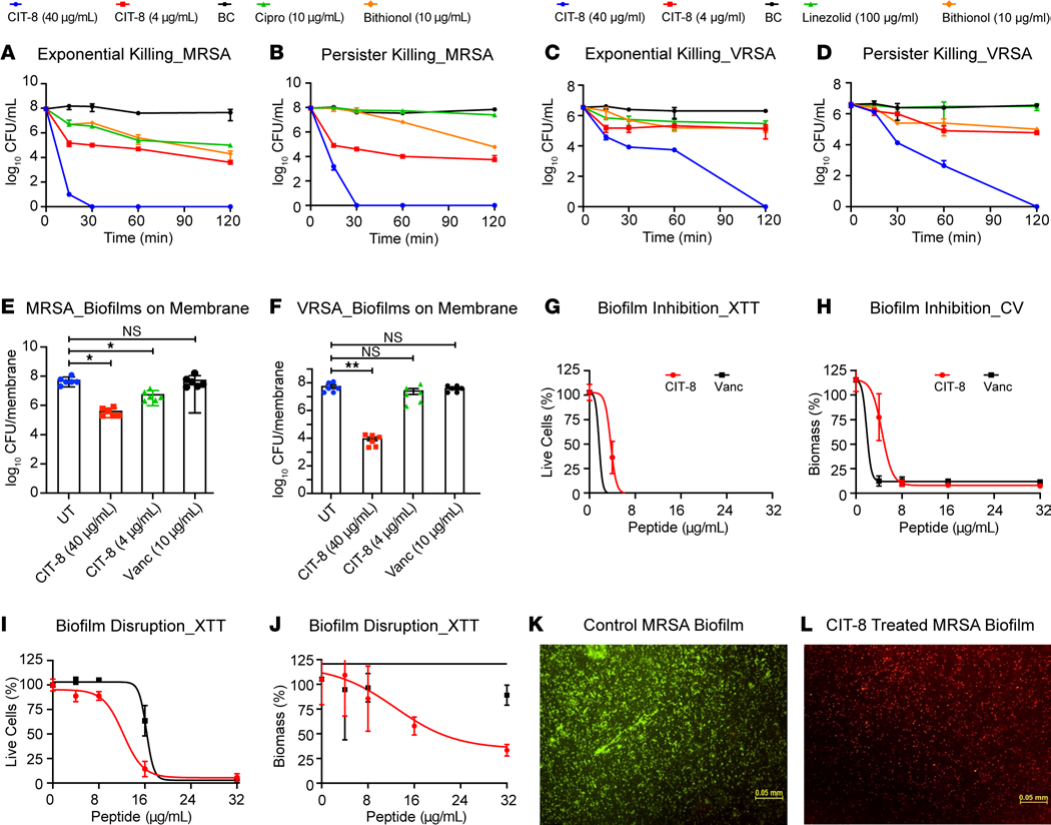
Antibiofilm and antipersister activity of CIT-8. (**A** and **B**) Killing kinetics of CIT-8 against *S*. *aureus* MW2 in (**A**) exponential phase and (**B**) gentamicin-induced persister cells at concentrations of 4 and 40 μg/mL, compared with untreated bacterial (BC) control, colony forming units (CFU) counts were monitored for 120 minutes (*n* = 2, replicated thrice). (**C** and **D**) Killing kinetics of CIT-8 against *S*. *aureus* strain VRS1 in (**C**) exponential phase and (**D**) gentamicin-induced persister cells at 4 and 40 μg/mL, CFU counts were monitored for 120 minutes (*n* = 2, replicated thrice). We included ciprofloxacin (cipro) (at 10 μg/mL) and linezolid (at 100 μg/mL) as antibiotic controls and bithionol (at 10 μg/mL) as a positive control. (**E** and **F**) Disruption of 24 hour established biofilms of (**E**) MRSA (*S*. *aureus* MW2), and (**F**) VRSA (*S*. *aureus* VRS1) by CIT-8, measured as log reductions in bacterial loads on solid membranes treated with 4 and 40 μg/mL of CIT-8 (*n* = 6, *P < 0.05 by 1-way ANOVA followed by Dunnett’s multiple comparison test). We included 10 μg/mL vancomycin (Vanc) as control. (**G** and **H**) Inhibition of *S*. *aureus* MW2 biofilm formation by CIT-8 at concentrations ranging from 4–32 μg/mL after 24 hours of treatment, assessed using (**G**) live-cell viability (XTT assay), and (**H**) biomass quantification (crystal violet staining) (*n* = 3, replicated twice). (**I** and **J**) Disruption of 24 hours *S*. *aureus* MW2 established biofilms by CIT-8 at 4–32 μg/mL, evaluated by (**I**) reductions in live-cell viability (XTT assay) and (**J**) biomass loss (crystal violet staining) (*n* = 3, replicated twice). (**K** and **L**) Fluorescence microscopy images (10×) of 24 hour-established *S*. *aureus* MW2 biofilms stained with live/dead staining, (**K**) untreated control, and (**L**) biofilms treated with 32 μg/ml of CIT-8. Scale bars: 0.05 mm.

To further determine the antimicrobial spectrum of CIT-8, we tested the rate of killing of CIT-8 against exponentially growing cells as well as *S*. *aureus* persister cells generated by growing them to a stationary phase in the presence of gentamicin (20 μg/mL). At 10 × MIC, CIT-8 killed almost all exponentially growing MRSA cells and completely eradicated approximately 1 × 10^8^ CFU MRSA persister cells within 30 minutes of exposure (Figure 3, A and B). Similarly, CIT-8 (at 10 × MIC) also eradicated both exponential and VRSA persister cells within 120 minutes of treatment (Figure 3, C and D). Ciprofloxacin (at 10 μg/mL) reduced exponentially growing MRSA cells by 1.5 logs within 120 minutes but did not have any effect on the MRSA persister cells. A positive control, bithionol, previously shown to have anti-MRSA-persister–cell activity (28), reduced the titers of both exponential and MRSA persister cells by 1.8 and 1.5 logs, respectively. Likewise, bithionol also reduced exponential and VRSA persister cells by 1.0 and 1.5 logs within 120 minutes of exposure. However, another clinically used antibiotic that targets the bacterial protein synthesis linezolid (at 100 μg/mL), only reduced the exponentially growing VRSA by 0.7 logs and had no impact on VRSA persister cells with in 120 minutes of treatment. As a positive control, a potent AMP, melittin, known to disrupt bacterial membranes (29), showed rapid killing kinetics similar to CIT-8, eliminating approximately 1 × 10^8^ and 1 × 10^7^ CFU of both exponential and persisters cells of MRSA and VRSA, respectively, within 120 minutes of peptide exposure (Supplemental Figure 5, A–D).

Because biofilms are tolerant to many antibiotics, we also tested the activity of CIT-8 on biofilms of MRSA and VRSA grown on a solid membrane support (Figure 3, E and F). CIT-8 (at 40 μg/mL) reduced biofilm burdens of MRSA and VRSA by 2.2 and 3.8 logs, respectively, while the control AMP, melittin, at the same concentration, achieved reductions of 1.85 and 3.9 logs on biofilms grown on a solid membrane (Figure 3, E and F, and Supplemental Figure 5, E and F).

We also tested the ability of the CIT-8 peptide to inhibit and disrupt *S*. *aureus* MW2 biofilms in a biofilm disassembly assay (Figure 3, G–J). During the *S*. *aureus* MW2 biofilm formation stage, CIT-8 killed 50% of the biofilm cells at 4 μg/mL and reduced the bacterial biomass by approximately 50% at 6 μg/mL (MBIC_50_; Figure 3, G and H). Similarly, in the case of an *S*. *aureus* MW2 biofilm that had been established for 24 hours, CIT-8 at 12 μg/mL killed 50% of the live bacterial cells (Figure 3I) and effectively disrupted 50% of the biomass contents at 24 μg/mL (MBEC_50_) (Figure 3J). Compared with CIT-8, the AMP control, melittin, effectively inhibited *S*. *aureus* MW2 biofilm formation and disrupted 24-hour established biofilms with lower MBIC_50_ and MBEC_50_ values of 4.8 and 2.3 μg/mL, respectively (Supplemental Figure 5, G–J).

We further employed confocal laser scanning microscopy to confirm the antibiofilm effect of CIT-8 by staining biofilms with SYTO9 (for staining live cells) and propidium iodide (for staining dead cells). At 32 μg/mL, CIT-8 treatment resulted in a predominantly red–colored biofilm mass, indicating a high proportion of dead cells (Figure 3K), similar to biofilms treated with the control AMP, melittin, at the same concentration (Supplemental Figure 5, K–M). In contrast, untreated biofilms appeared predominantly green, indicating live bacterial cells (Figure 3L).

### Mechanism of action of CIT-8 peptide in vitro.

We conducted peptide-membrane interaction studies employing circular dichroism, NMR, and Molecular Dynamics (MD) studies to elucidate the mode of action (MOA) of CIT-8. First, we performed circular dichroism spectroscopy of CIT-8 in the presence of SDS micelles to determine the conformational changes in the secondary structure of CIT-8 upon its interactions with bacterial membrane mimics (Supplemental Figure 6). Importantly, the CD spectrum of CIT-8 showed 2 apparent dips, at 208 and 222 nm, indicating that the peptide developed helicity upon exposure to SDS, confirming a real-time membrane interaction (Supplemental Figure 6).

**Figure 4:**
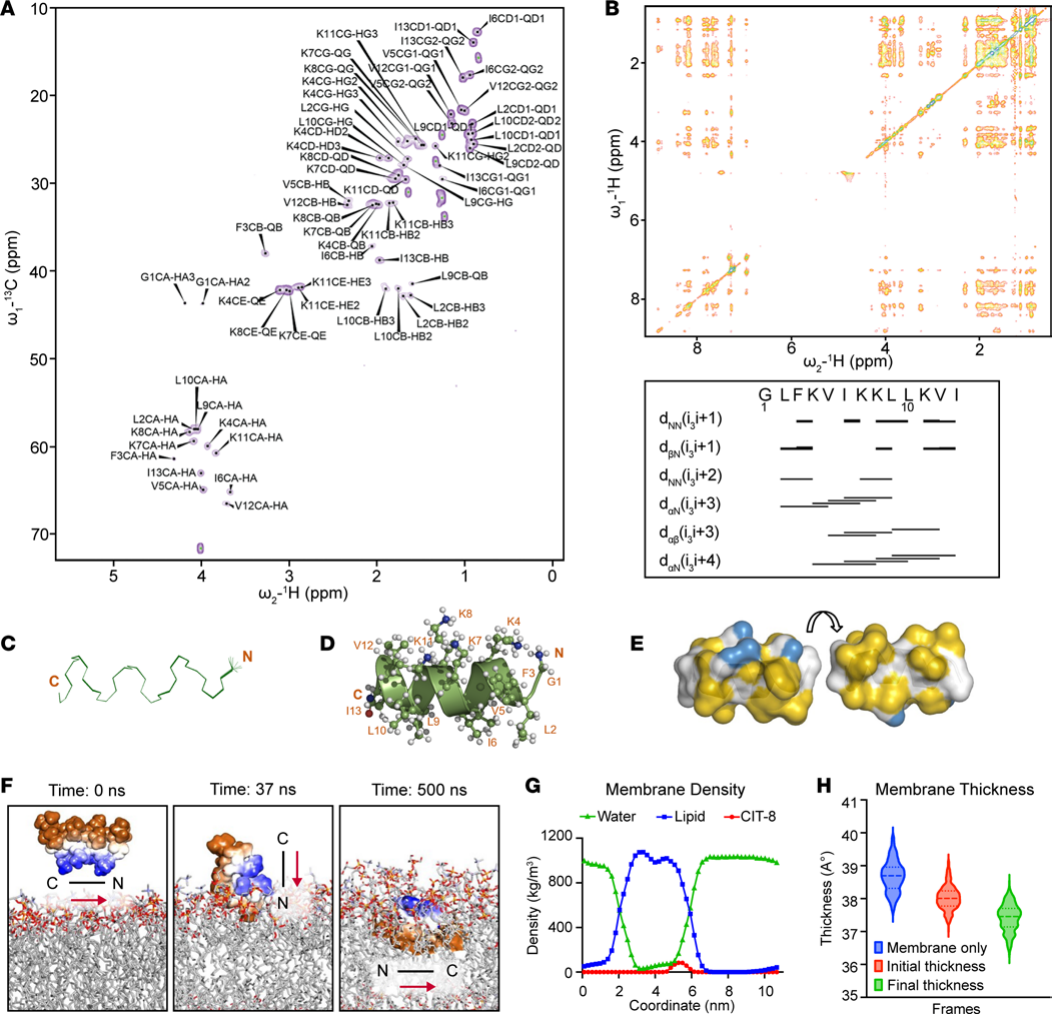
Structural insights to membrane targeting by CIT-8. (**A**) Natural abundance 2D- ^13^C-HSQC spectrum of CIT-8. (**B**) 2D-NOESY spectrum and summary of important NOESY distance restraints used in the CIT-8 structure calculation. (**C**) CIT-8 NMR solution structure ensemble. (**D**) Ribbon representation of the first conformer in the ensemble. (**E**) Two surface representations obtained by 180º rotation along the x-axis showing the distribution of hydrophobic (yellow) and charged (blue) residues. All structure figures were prepared in Pymol using the YRB script. (**F**) Snapshot of an all-atom MD simulation of peptide CIT-8 in the presence of DOPC:DOPG (7:3) mimetic membrane model showing complete peptide insertion at 500 ns. Blue, charged residues; brown, hydrophobic residues. (**G**) Changes in membrane lipid density upon CIT-8-induced water perturbation. (**H**) Changes in membrane thickness upon CIT-8 interaction with model membrane.

Next, to gain additional details on CIT-8 membrane interactions at the molecular level, we used NMR to determine the 3D structure of CIT-8 in SDS (Figure 4, A–E). Figure 4, A and B show homo- and heteronuclear 2D NMR spectra. All residues of the NMR structure ensemble fell in the 100% favorable regions of the Ramachandran plot, and there were no distance violations greater than 0.15 Å (Figure 4C). CIT-8 adopted a helical structure (Figure 4D) with an even distribution of hydrophilic and hydrophobic residues on opposite faces (Figure 4E). Supplemental Table 4 summarizes the NMR structural calculation statistics. CIT-8 had a well-defined hydrophilic face comprising the lysine residues at positions 4, 7, 8, and 11 and a hydrophobic face comprising leucine 2, isoleucine 6, leucine 9, leucine 10, and isoleucine 13. The peptide-membrane interface was composed of glycine 1, phenylalanine 3, and valine 5. The spatial arrangement of the hydrophobic amino acids imparted a broad hydrophobic surface that interacted with SDS membranes (Figure 4E).

To explore changes in the membrane architecture upon CIT-8 interactions with bacterial membranes, we performed MD simulations of CIT-8 in the presence of a DOPC:DOPG (7:3) model membrane mimicking Gram-positive bacterial membranes (30). CIT-8 bound to the membrane within the first 37 nanoseconds (ns), with the N-terminus protruding into the membrane surface (Figure 4F). The key residues that formed hydrogen bonds were lysine 7, which interacted with DOPC with 31% occupancy, and lysine 11, which interacted with DOPG with 56% occupancy (Supplemental Table 5). CIT-8 remained bound to the outer leaflet of the bilayer for the rest of the simulation (500 ns) and remained amphipathic inside the membrane (Figure 4F). The partial density plot of the system confirmed the position of CIT-8 inside the membrane during the simulation time period. We observed an incremental change in water density (indicated in green) and a simultaneous decrease in lipid density (indicated in blue) at around 5 nm, indicating water perturbation in the membrane upon CIT-8 insertion (Figure 4G). The CIT-8 peptide was located predominantly at about 1.75 nm inside the outer membrane leaflet (Figure 4G). Interestingly, CIT-8 binding resulted in membrane thinning (Figure 4H) and an increase in the membrane surface area per lipid ratio (Supplemental Figure 7), indicating destabilization of the model membrane upon CIT-8 interaction, which likely facilitates membrane permeabilization.

### MOA of CIT-8 peptide in live S. aureus MW2 bacterial cells.

**Figure 5:**
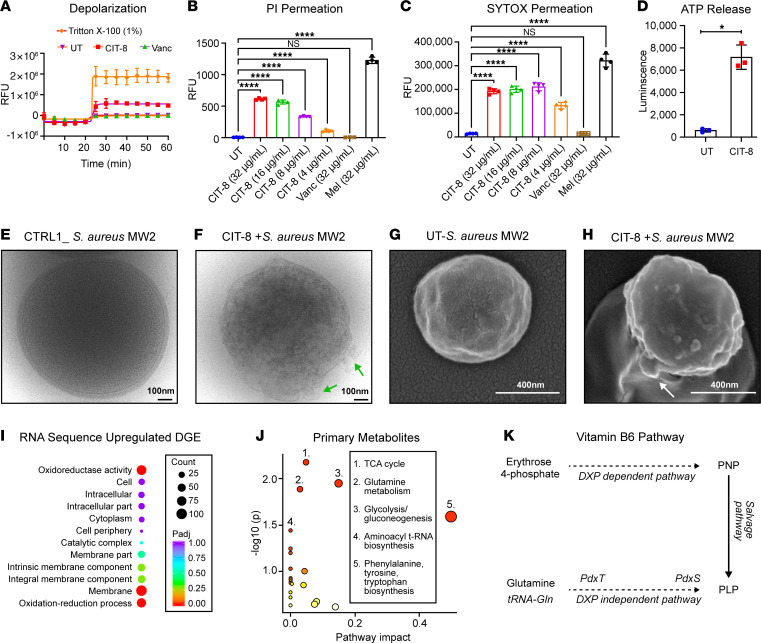
Mechanism of action of CIT-8 and associated stress response by MRSA. (**A**) Fluorescence-based, DIBAC4(3)-assisted *S*. *aureus* MW2 membrane depolarization caused by CIT-8 peptide (at 32 μg/mL) monitored for 40 minutes after peptide exposure (*n* = 3). (**B** and **C**) Fluorescence-based membrane permeability of *S*. *aureus* MW2 treated with CIT-8 (4–32 μg/mL), untreated bacteria (UT), vancomycin (Vanc), and melittin (Mel) at 32 μg/mL after 60 minutes, assessed using (**B**) PI and (**C**) SYTOX Green fluorescence (*n* = 4, ****P < 0.0001 by 1-way ANOVA followed by Dunnett’s multiple comparison test). (**D**) ATP release from *S*. *aureus* MW2 upon CIT-8 (at 32 μg/mL) interaction for 30 minutes (* denotes *P* < 0.05 by Student’s *t*-test, unpaired 2-tailed). (**E**) Cryo-EM image of control *S*. *aureus* MW2. (**F**) Cryo-EM image of *S*. *aureus* MW2 treated with CIT-8 at 80 μg/mL for 60 minutes (green arrows indicate membrane perturbation). (**G**) SEM image of control *S*. *aureus* MW2. (**H**) SEM image of *S*. *aureus* MW2 treated with CIT-8 at 40 μg/mL for 60 minutes (white arrows indicate membrane blebbing). (**I**) RNA-seq–derived differential gene expression (DGE) of significantly upregulated genes (*n* = 2 samples, *P* < 0.05, calculated using DESeq2 (76) in *S*. *aureus* MW2 by CIT-8 (at 2 μg/mL) treated for 30 minutes. (**J**) Pathway analysis of the targeted metabolome of *S*. *aureus* MW2 treated with peptide CIT-8 at 4 μg/mL for 30 minutes, revealing significant alterations in key stress and metabolic pathways (*n* = 3, significant metabolite in pathways were determined by their *P* < 0.05 obtained by Student’s *t* test, unpaired, 2-tailed). (**K**) Stress responsive vitamin B6 pathway in *S*. *aureus* MW2, indicating key regulatory genes (*pdxT* and *pdxS* revelated by our RNA-seq analysis) and metabolite (Erythrose 4-phosphate, identified by our targeted metabolomics analysis) positions in the pathway. Scale bars: 100 nm (**E** and **F**); 400 nm (**G** and **H**).

We conducted additional biophysical experiments to observe the effect of CIT-8 on live *S*. *aureus* cells. We monitored the change in transmembrane potential induced by CIT-8 on *S*. *aureus* MW2 cells using a DIBAC_4_(3) fluorescence-based dye assay. Upon interaction with *S*. *aureus* MW2 cells, CIT-8 caused a rapid increase in DIBAC_4_(3) fluorescence, although the intensity was lower than with Triton-X 100 (Figure 5A). To confirm membrane disruption, we performed a fluorescence-based dye permeation assay using propidium iodide dye. *S*. *aureus* MW2 cells treated with CIT-8 exhibited increased fluorescence compared with untreated controls. This increase in fluorescence was due to the entry of membrane-impermeable propidium iodide into the cells where it bound to DNA, indicating that CIT-8 compromised the bacterial membrane (Figure 5B). Vancomycin, which targets the cell wall rather than the membrane, did not induce fluorescence, while melittin, which disrupts the bacterial membrane, resulted in elevated fluorescence levels.

Results from another membrane-impervious dye, SYTOX Green, were consistent with the membrane-disrupting hypothesis for CIT-8. As shown in Figure 5C, *S*. *aureus* MW2 cells treated with CIT-8 at concentrations of 4–32 μg/mL for 1 hour exhibited significantly increased fluorescence levels, attributed to the formation of SYTOX:DNA complexes.

In order to evaluate whether the ML-designed peptides from three additional templates have improved membrane disrupting properties, we conducted membrane permeation experiments using both propidium iodide (Supplemental Figure 8A) and SYTOX green (Supplemental Figure 8B) dyes. The ML-designed hylaseptin P1, mastoparan-L and r-CAMEL–derived peptides showed increased fluorescence levels when coincubated with *S*. *aureus* MW2, indicating compromised bacterial membranes in all three cases in the presence of the ML-designed peptides, whereas the WT peptides templates did not increase the propidium iodide or SYTOX fluorescence (Supplemental Figure 8).

We also measured ATP leakage from *S*. *aureus* MW2 cells in the presence of CIT-8 (Figure 5D) using a luminescence-based ATP assay. Treatment with CIT-8 increased luminescence levels compared with untreated controls, suggesting ATP leakage (Figure 5D).

To obtain visual representation of the membrane impact caused by CIT-8, we performed cryo-transmission and scanning electron microscopy (Figures 5, E–H). Cryo-EM showed that CIT-8 treatment of *S*. *aureus* MW2 cells caused membrane disruption and perturbation (Figure 5F), compared with untreated cells (Figure 5E), which had intact cytoplasmic membranes. In addition, the “transparency” exhibited by CIT-8-treated cells also suggested leakage of cytoplasmic materials through the damaged cell membrane (Figure 5F). In agreement with the cryo-EM observations, SEM images of the CIT-8–treated *S*. *aureus* MW2 cells showed a heterogenous, rough membrane surface (Figure 5H). The damaged bacterial cells also showed blebbing, vacuoles, and invaginations (Figure 5H).

Finally, we tested the susceptibility to CIT-8 of an *S*. *aureus* *mprF* transposon mutant from the Nebraska Transposon Mutant Library (NTML) (31), which lacks the capability to transfer a lysyl group on the bacterial membrane surface. Deletion of *mprF* gene in *S*. *aureus* decreases the net negative charge of the bacterial membrane, thereby reducing the electrostatic interactions with cationic AMPs (32). We observed a 2-fold reduction in MIC of CIT-8 against *mprF* transposon mutant compared with the *S*. *aureus* JE2 background (Supplemental Table 6). The increased antimicrobial potency of CIT-8 in the *mprF* transposon mutant, which has an increased net negative charge, is consistent with the conclusion that CIT-8 targets the cell membrane of the bacteria.

### Extended effects of CIT-8 in bacterial physiology.

To identify potential CIT-8 targets in *S*. *aureus* MW2, we carried out RNA-seq analysis. At 0.5× MIC, CIT-8 affected the expression of several genes involved in membrane regulation, the vitamin B6 pathway, and purine and aminoacyl tRNA biosynthesis (Supplemental Tables 7 and 8). Treatment with CIT-8 upregulated 179 genes by more than 2-fold (*P* < 0.05) (Figure 5I and Supplemental Table 7). Several essential genes involved in the DXP-independent pathway for vitamin B6 production were upregulated, including *pdxT* (5.9-fold) and *pdxS* (5.3-fold). CIT-8 treatment also caused an upregulation of key enzymes in purine biosynthesis, namely *purL*, *purS*, *purQ*, and *purH*, by 5.2-, 5.0-, 5.0-, and 3.6-fold, respectively. CIT-8 downregulated 267 genes by at least 2-fold or more (*P* < 0.05, Supplemental Table 8). Notably, several downregulated genes are involved in aminoacyl tRNA biosynthesis, including *tRNAGln* (8.5-fold), *tRNAser* (8.4-fold), *tRNAarg* (7.7-fold), *tRNAcys* (6.8-fold), and other *aminoacyl tRNA* genes (Supplemental Figure 9 and Supplemental Table 8).

Next, we performed targeted metabolomics to complement the RNA-seq data. We measured the change in primary metabolite levels in CIT-8–treated *S*. *aureus* MW2 cells using LC-MS/MS. From PLSDA plots, we found that CIT-8 at 8 μg/mL caused substantial metabolic alterations in *S*. *aureus* MW2 when compared with untreated controls (Supplemental Figure 10). In the CIT-8–treated *S*. *aureus* MW2 cells, ten primary metabolites were significantly altered (*P* value < 0.05) (Supplemental Figure 11). Pathway impact analysis showed that CIT-8 affected amino acid synthesis (e.g., phenylalanine, tyrosine, and tryptophan), aminoacyl *tRNA* biosynthesis, glycolysis, the TCA cycle, metabolism of glutamine, glycerophospholipid synthesis, and pyrimidine biosynthesis (Figure 5J). Our targeted metabolomics also revealed a downregulation of erythrose-4-phosphate, a key component of the DXP-independent pathway for vitamin B6 biosynthesis (33).

To determine whether the effects of CIT-8 on genes involved in vitamin B6 biosynthesis is a consequence of these genes being a direct target of CIT-8 or an indirect stress response by the bacteria, we tested whether a *pdxS*-transposon mutant (pyridoxal 5’-phosphate synthase subunit *PdxS*) was more susceptible or resistant to CIT. However, CIT-8 had the same MIC for the *pdx-*transposon mutant and the parent *S*. *aureus* strain JE2, even in the presence of 100 μM supplemental external vitamin B6 (Supplemental Table 9), suggesting that CIT-8 is not directly targeting the vitamin B6 biosynthetic pathway.

### In vivo therapeutic efficacy of CIT-8 peptides.

**Figure 6:**
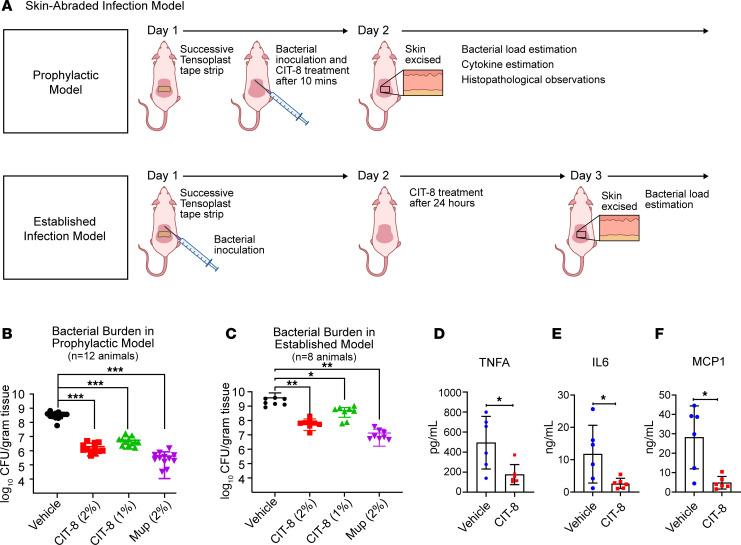
In vivo efficacy of CIT-8 in a skin-abraded murine infection model infected with *S*. *aureus* MW2. (**A**) Schematic representation of the skin-abraded murine model representing both prophylactic and established models. (**B**) Quantified bacterial load from skin specimens collected from mice infected with exponential phase *S*. *aureus* MW2 and treated after 10 minutes (representing a prophylactic model) with CIT-8 (2% w/w), CIT-8 (1% w/w), and mupirocin (2% w/w) ointments compared with vehicle control (*n* = 12, ****P < 0.0001, **P < 0.01, *P < 0.05 calculated by 1-way ANOVA followed by Dunnett’s multiple comparison test). (**C**) Quantified bacterial load from skin specimens collected from mice infected with exponential phase *S*. *aureus* MW2 and treated after 24 hours (representing an established infection model) with CIT-8 (2% w/w), CIT-8 (1% w/w), and mupirocin (2% w/w) ointments compared with vehicle control (*n* = 8, **P < 0.01, *P < 0.05 calculated by 1-way ANOVA followed by Dunnett’s multiple comparison test). Cytokine estimations for (**D**) TNFA, (**E**) IL6, and (**F**) MCP1 (*n* = 6, *P < 0.05, calculated by Student’s *t* test, unpaired, 2-tailed) in murine skin treated with CIT-8 (2% w/w) after 10 minutes of bacterial infection in a prophylactic model.

We tested the in vivo efficacy of CIT-8 using an abraded skin infection model where mice were infected with 1 × 10^7^ CFU of *S*. *aureus* MW2 (Figure 6). We formulated a CIT-8 peptide ointment (1% and 2% w/w) using white petroleum jelly as a base and applied it to treat *S*. *aureus* MW2 wound infections in a prophylactic (acute) and an established infection model (Figure 6A). A single treatment of CIT-8 (2% w/w) applied after 10 minutes of bacterial inoculation (representing a prophylactic/acute model) reduced *S*. *aureus* MW2 burden by 2.3 log (*P* < 0.0001) compared with the vehicle-treated animal group. The CIT-8 (1% w/w) formulation lowered the burden by 1.7 log (*P* < 0.0001) (Figure 6B). For comparison, we included mupirocin (2% w/w) ointment as a positive control, which reduced the bacterial burden by 2.8 log (*P* < 0.0001). Similarly, in an established infection model, a single dose of CIT-8 applied after 24 hours of *S*. *aureus* MW2 inoculation reduced 0.85 log (*P* < 0.0208) (at 1% w/w) and 1.8 log (*P* < 0.0088) (at 2% w/w) of *S*. *aureus* MW2 compared with the vehicle-treated animals (Figure 6C). The antibiotic control mupirocin (2% w/w) ointment decreased the bacterial load of S. *aureus* MW2 by 2.4 log (*P* < 0.0078). The CIT-8 peptide–treated (2% w/w) skin in the prophylactic model also displayed reduced levels of proinflammatory cytokines and chemokines, including TNFA (2.5-fold, *P* = 0.019), IL6 (5-fold, *P* = 0.039), and MCP1 (5-fold, *P* = 0.006) (Figure 6, D–F).

A repeat of the experiment testing the efficacy of CIT-8 in the prophylactic (acute) skin abrasion model using *n* = 8 animals (Supplemental Figure 12) showed that treatment with CIT-8 (2% w/w) reduced the *S*. *aureus* MW2 bacterial burden by 2.1 log (*P* < 0.0034) and that 1% w/w CIT-8 reduced it by 1.4 log (*P* < 0.0045) (Supplemental Figure 12). In this experiment we also carried out histopathology analysis of skin tissues using H&E and Gram stains (Supplemental Figure 13). Abraded skin with vehicle control (no peptide) showed several key features: (a) a disorganized epidermal layer (broken epidermis at some points), (b) sloughing of the keratin layer with the presence of a dense population of poly mononuclear cells (PMN), and (c) mononuclear cells (MN) in both the epidermis and dermis, indicating enhanced inflammation (Supplemental Figure 13 Panel A). The untreated skin also showed bacterial biofilms, as demonstrated by Gram staining (Supplemental Figure 13 Panel B). CIT-8 treatment (2% w/w formulation) resulted in a more intact epidermis, reduced numbers of PMN and MN (less inflammation), and no bacterial biofilm patches in contrast with vehicle-treated mice (Supplemental Figure 13), suggesting that CIT-8 may have protective activity against *S*. *aureus* on the skin.

## Discussion

Addressing the world-wide rise in the antimicrobial resistance requires the development of a next generation of antimicrobial agents (34). AMPs represent a class of antiinfective molecules with the potential to act as new antibiotics (3). Here, we present an AMP design strategy to accelerate AMP therapeutic discovery. Our method combines noncomputational, traditional strategies (template-based design), including truncation (6), substitution (7), and structure-based approaches (10) with ML-guided peptide optimization, in a single comprehensive AMP design strategy. Using this methodology and starting from a natural AMP with minimal antistaphylococcal activity, we developed CIT-8, a short (13-mer) citropin-1.1–derived peptide. Leveraging the entire sequence space information present in AMP databases further refined our approach. Importantly, as proof of concept, CIT-8 demonstrated extensive antistaphylococcal activity, killed MRSA and VRSA persisters, inhibited biofilm formation, disrupted established biofilms, and was effective at reducing MRSA burden in an established infection model using skin-abraded mice. CIT-8 also suppressed the levels of proinflammatory cytokines.

In our study, we truncated citropin 1.1 to stabilize the amphipathic α-helix by deleting the glycine expansion on the C-terminus. Citropin 1.1 includes an α-helical structure along the entire backbone from residue 1–12 in presence of SDS micelles (35). The presence of glycine and prolines destabilizes the α-helix (36). Because positively charged residues of the peptides interact with the negatively charged phospholipid head groups for effective peptide-membrane binding (37), we substituted the negatively charged aspartic acid and the neutral serine on the hydrophilic face to improve peptide-membrane ionic interaction. We also filled a hydrophobic surface gap with bulkier amino acids on the template peptide to further improve membrane binding on the hydrophobic face.

Similar to our AMP design approach, in a previous study, the substitution of charged amino acid residues for noncharged polar residues on the polar face of *Aristicluthys nobilia* IFN-I and Alyteserin 1c–derived peptides was shown to increase antimicrobial activity (38). Additionally, Wang et al. developed a potent anti-MRSA peptide, 17BIPHE2, by filling the hydrophobic gaps in GF-17d3 using bulkier biphenyl residues (16). However, in these aforementioned studies, the amino acid substitutions were guided solely by QSAR, which lacks the capability to guide the selection of the most appropriate amino acids. Our report integrates ML with QSAR to identify evolutionarily favored amino acids to optimize amino acid substitutions.

To select the optimal amino acid candidates for the substitutions, we used unsupervised ML to explore the physicochemical space of more than 14,000 functional AMPs. ML-based approaches in drug discovery are widely used to identify or create new antimicrobial candidates and typically involve training a model on a dataset to either predict the antimicrobial activity of a given set of peptides or generate new potential AMP candidates (18, 20, 21, 39). ML-based models for de novo AMP design include AMPGAN (40), which utilizes a generative adversarial network, and CFPS (41), which uses deep generative variational autoencoders. To predict antimicrobial activity, models such as CalcAMP (42) and iAMPCN (43) leverage convolutional neural networks. Unlike these models, the approach described in our work does not rely on prediction or sequence generation for new candidates but instead utilizes ML algorithms to understand the inherent characteristics of existing AMPs. Using k-means clustering, we selected peptide sequences that had similar properties to known helical antistaphylococcal peptides (13, 44) and constructed a knowledge graph to derive the AGO and APO patterns that guided the substitutions (45, 46). By transforming complex peptide sequence data into interpretable patterns, our method incorporates the extraction of evolutionary knowledge that informs the rational design of peptides (19, 21, 25, 47). In fact, our hybrid approach identified the most promising peptides across the four templates, citropin 1.1, hylaseptin P1, mastoparan-L, and r-CAMEL, in a single-step method, selecting those with the highest number of AGO instances.

In MRSA, antimicrobial resistance is linked to life-threatening infections, such as pneumonia, sepsis, and endocarditis (48). The situation is further complicated by the presence of *S*. *aureus* bacteria in alternative physiological states such as biofilms and persister cells, which are often neglected in early antimicrobial drug discovery stages even though they contribute substantially to chronic, relapsing infections (49). Interestingly, the citropin 1.1 template demonstrated weak anti-MRSA activity (MIC of 32 μg/mL against *S*. *aureus* JE2, a virulent community acquired *S*. *aureus* USA300 isolate) (50). However, CIT-8, which we derived from citropin 1.1, exhibited potent antistaphylococcal activity against a wide range of antibiotic-resistant *S*. *aureus*.

Moreover, CIT-8 eradicated MRSA and VRSA persisters that survived in extremely high doses of gentamicin (20 μg/mL). Given that persister cell membranes are more robust than those of growing cells, and even minor structural changes in small molecules can alter their antipersister abilities (51), our design strategy of informed structural modifications increased the efficacy of the derivative peptide CIT-8 against the bacterial membrane, resulting in a peptide, CIT-8, with antipersister properties. CIT-8 also inhibited biofilm formation and disrupted established MRSA biofilms that resulted in lower bacterial cell density and a higher prevalence of dead cells, similar to a lactoferrin and cathelicidin hybrid AMP, Lf-KR-12, which reportedly weakens the biofilm matrix by forming pores within lipid components (52).

Notably, CIT-8 demonstrated potent antistaphylococcal activity even in the presence of physiological salts and serum concentrations. Salt ions compete with AMPs for bacterial membranes, decreasing AMP-membrane binding effectiveness (52), as seen in AMPs such as human defensins, linear and tetrameric LfcinB6, and gramicidin S (52, 53). Similarly, the presence of serum influences the antimicrobial potencies of several AMPs because of AMP binding to serum proteins such as albumin and apolipoproteins (54). However, AMPs with high cationic charges and hydrophobicity such as the arginine-rich decamer peptides D5 and D6, cecropin-4–derived C18 peptides, human cathelicidin-derived peptides 106 and 110, and SAAP-148, are known to mask the salt and serum effects (15, 55, 56). Our approach of enhancing CIT-8 binding to the bacterial membrane ultimately increased the charge and hydrophobicity of the peptide, which appear to be key factors for its serum and salt resistance.

In our peptide design, the replacement of neutral amino acids with charged ones on the hydrophilic face and the incorporation of hydrophobic substitutions on the nonpolar face substantially enhanced the amphipathicity in CIT-8. Our NMR studies suggest that CIT-8 is amphipathic and has a broad hydrophobic-membrane–interacting surface, similar to DFTamP1 (13) and DFT503 (32). In general, amphipathicity enables α-helical AMPs to bind effectively to anionic bacterial membranes and exert their antimicrobial effects (3, 57). In addition, our MD studies showed that CIT-8 positions itself inside the outer leaflet, parallel to the phospholipid bilayer in a DOPC:DOPG (7:3) model membrane. Because CIT-8 does not have the horizontal length to form a toroidal pore, it cannot align perpendicularly to the lipid membrane, as longer peptides such as magainin 2 (58) or melittin can (59). SEM and cryo-TEM imaging suggest that CIT-8 created large membrane defects on the *S*. *aureus* membrane. CIT-8 might adopt an alternate membrane deforming strategy that makes large membrane deformations similar to a carpet model (60).

Apart from targeting the bacterial membrane, CIT-8 also impacts other bacterial physiological functions. Our RNA sequencing of *S*. *aureus* MW2 suggests that CIT-8 treatment altered genes involved in vitamin B6 biosynthesis, purine biosynthesis, and aminoacyl *tRNA* biosynthesis. Vitamin B6 is known to serve as a cofactor for many enzymes related to amino acid metabolism in *Bacillus subtilis* (61). Purine metabolism is linked to peptide-induced stress signals, as observed in LfcinB and Bactenecin 7 peptides (62). In addition, the aminoacyl *tRNA* biosynthesis pathways are associated with the bacterial response to reduced protein synthesis due to amino acid stress (63). To validate our RNA-seq–based finding regarding the impact of CIT-8 on the vitamin B6 biosynthetic pathway, we conducted targeted metabolomics. Our results revealed a downregulation of erythrose-4–phosphate, a key component of the DXP-independent pathway for vitamin B6 biosynthesis (33). This simultaneous downregulation of erythrose-4–phosphate and upregulation of *pdx* genes (from RNA-seq analysis) may suggest a compensatory feedback mechanism. The reduction in erythrose-4–phosphate levels may indicate a disruption in the DXP-independent pathway, triggering an adaptive response in which the cell upregulates *pdx* gene expression to sustain vitamin B6 production. Given the concurrence between RNA-seq and metabolomics data regarding alterations in the vitamin B6 pathway, we sought to confirm whether this pathway contributes to additional MOA of CIT-8 against *S*. *aureus* (Figure 5K). However, our evaluation of the NTML *pdxS* transposon mutant (31), which did not exhibit any susceptibility changes to CIT-8, is consistent with the conclusion that CIT-8 primarily targets the bacterial membrane for bacterial killing and the impact on the vitamin B6 pathway is most likely a component of an indirect stress response. Nevertheless, further studies are needed to elucidate the regulatory mechanisms of the vitamin B6 biosynthetic pathway and its potential involvement in bacterial adaptation to CIT-8 exposure.

Importantly, CIT-8 (2% w/w) in petroleum-based formulation was effective in reducing MRSA bacterial burden, protected the skin, resulted in fewer epidermal and dermal deformities, and prevented biofilm formation in a murine model of *S*. *aureus* wound infection (15). Wound injuries represent over 4% of all emergency department visits in the United States (64) and are often linked with biofilm infections (65). Unlike traditional antibiotics, AMPs offer promising potential for wound care due to their broad antimicrobial spectrum resulting from their membrane-targeting abilities (3), which also mitigate the risk of resistance development (3). While several AMPs, including SAAP-148 (15), 17BIPHE2 (16), P60.4Ac (66), D-IK-8 (67), WR-12 (67), and piscidin 3 (68) have demonstrated potent topical antistaphylococcal effects, we believe that our approach to designing shorter, potent, peptides holds substantial potential.

In summary, by integrating traditional peptide design methods with unsupervised ML techniques, we developed an AMP design strategy that enabled us to make informed modifications to a peptide template, resulting in the creation of a short and potent AMP. Our approach, which involved reducing nonessential regions of the peptide template and selectively substituting amino acids at strategic positions, not only fine tuned the desired antimicrobial characteristics but also minimized the number of peptide candidates to be chemically synthesized. Furthermore, in a proof-of-concept study, we successfully enhanced citropin 1.1 to develop CIT-8, demonstrating the practical utility of our computational approach. Because CIT-8 exhibited potent antimicrobial activity against MRSA in vitro as well as in a localized mouse model of MRSA infection, development of CIT-8 as an antistaphylococcal agent looks promising. Moreover, utilizing our strategy to develop short-length AMP candidates can reduce chemical synthesis costs and help make the commercial development of AMPs easier, faster, and more targeted.

## Methods

### Sex as a biological variable.

Only female mice were used in this study. Sex was not considered as a biological variable in the experimental design or analysis. No specific rationale guided the selection of female mice, and given the immunological nature of the model, we did not anticipate sex-based differences in the outcomes.

### Data collection and processing.

We downloaded all the peptide sequences that had documented antimicrobial activity against any Gram-positive or Gram-negative bacteria from the Antimicrobial Peptide Database (APD) (https://aps.unmc.edu/) (69), the Database of Antimicrobial Activity and Structure of Peptides (DBAASP) (https://dbaasp.org/home) (70), and the Database for Antimicrobial Peptides (dbAMP) (https://awi.cuhk.edu.cn/dbAMP/) (71). Details of data collection and processing are provided in the Supplemental Materials.

### Data visualization and clustering.

To visualize the peptides in a 2-dimensional plane, we employed the t-distributed Stochastic Neighbor Embedding (t-SNE) technique (72) and transformed the 5-dimensional PCP descriptors. We selected this nonlinear dimensionality reduction method because of its effectiveness in preserving local data structures and revealing patterns in high-dimensional datasets (72). Using matplotlib and seaborn for plotting, we created scatter plots to display the t-SNE–transformed data points. A different color was used for each antimicrobial activity classification to explore potential clusters or patterns.

We used k-means clustering, an unsupervised ML algorithm, to categorize the peptide sequences into distinct groups based on their physicochemical properties (73). We used this approach to uncover inherent patterns within our dataset, facilitating the identification of peptides with similar characteristics that might correlate with their antimicrobial activities or structural features. We utilized silhouette analysis to visualize the separation distance between the resulting clusters and to determine the number of clusters for k-means clustering (74).

### Knowledge graph and ontology.

To aid in the systematic analysis of peptide sequences and their fragments, we developed an ontology that captures the hierarchical relationship between peptides and peptide fragments. We defined our data model using the OWL 2 Web Ontology Language (https://www.w3.org/TR/owl2-overview/) and the ontology was constructed using the OWLReady2 Python library (codes are represented below) (75).

*with* onto:

*class* Fragment(Thing):

pass

*class* hasSequence(DatatypeProperty, FunctionalProperty):

domain = [Fragment]

range = [str]

*class* numberOfOccurrences(DatatypeProperty, FunctionalProperty):

domain = [Fragment]

range = [int]

*class* length(DatatypeProperty, FunctionalProperty):

domain = [Fragment]

range = [int]

*class* hasParent(ObjectProperty):

domain = [Fragment]

range = [Fragment]

*class* hasChild(ObjectProperty):

domain = [Fragment]

range = [Fragment]

inverse_property = hasParent

The ‘Fragment’ class serves as the class of the peptide sequences and their fragments. Each ‘Fragment’ is characterized by its amino acid sequence, its length, and the frequency with which the fragment occurs in the dataset. To model the hierarchical structure inherent to peptides and their fragments, we introduced the ‘hasParent’ object property and its inverse object property ‘hasChild.’ The ‘hasParent’ and ‘hasChild’ properties linked ‘Fragment’ instances to their immediate larger (parent) and smaller fragment (child), respectively, allowing for the tracing of a fragment’s lineage within a peptide sequence.

To populate the ontology, we generated all possible fragments of lengths 1–4 amino acids from the peptide sequences included in the dataset. For each fragment, we recorded its sequence, counted its occurrences across the dataset, and calculated its length. We then established the hasParent and hasChild relationships to interconnect fragments based on sequence containment.

### Identification of common parent sequences and peptide modification protocol.

To engineer peptide sequences with potentially enhanced functional properties, we instituted a protocol that leverages the ontology of peptide fragments to guide the substitution of amino acids at specific positions within the peptide chains. This methodological adaptation was driven by the hypothesis that the most common parental fragment sequences represent evolutionarily conserved and functionally relevant amino acid occurrence patterns. We termed these preferential occurrences of specific groups of amino acids predominantly in natural AMPs as amino acid group occurrences (AGOs). We also quantitated the number of instances these AGOs occurred as the number of occurrences (NOO) and the most pronounced amino acid percent occurrence (APO) at specific position of an AMP.

We constructed a SPARQL query to retrieve parent fragments of a given sequence along with their occurrence frequencies (source code is represented below).

PREFIX: <http://example.org/fragment_onto.owl#>

SELECT ?fragment ?parentFragmentSequence ?numberOfOccurrences

WHERE {

?fragment a:Fragment.

?fragment:hasSequence ?fragmentSequence.

FILTER(?fragmentSequence IN (“[fragment of interest]”))

?fragment:hasParent ?parentFragment.

?parentFragment:numberOfOccurrences ?numberOfOccurrences.

?parentFragment:hasSequence ?parentFragmentSequence.

}RDER BY DESC(?numberOfOccurrences)

To improve peptide-membrane interactions, we specifically targeted sequence modifications that were necessary, including the initial ionic charge-charge attachment of peptides to phospholipid head groups, followed by a hydrophobic interaction between the peptide hydrophobic amino acid side chains and membrane lipid tails to alter membrane conformation. The SPARQL query was then executed to provide the most common peptide sequences in which this fragment appeared as a subsequence. The amino acid at the target position was substituted with the amino acid from the most prevalent parent sequence, which extended the original fragment by one residue at the position of interest.

### Wet lab validation of the designed Citropin 1.1 peptides.

Supplemental Materials, Information, and Methods describes in detail the materials and procedures used in this study, bacterial strains (Supplemental Table 10) and growth conditions, peptide synthesis and characterization, followed by the in vitro and in vivo assays.

### Statistics.

All values are represented as mean ± SD. All statistical analyses for in vitro and in vivo experiments were performed using GraphPad Prism (version 10.3.0). Specific statistical methods are described in the figure legends. One-way ANOVA followed by Dunnett’s multiple comparisons test was used for comparisons among multiple groups, while unpaired 2-tailed Student’s *t* test was applied for comparisons between 2 groups. Differences were considered statistically significant at *P* < 0.05.

### Study approval.

All animal experiments were approved by the Institutional Animal Care and Use Committee (IACUC) under protocol IS00008451 at the Houston Methodist Research Institute (Houston, Texas, USA).

### Data availability.

Values for all data points in graphs are reported in the Supporting Data Values file. Data are available upon request. The RNA-Seq data is freely available under the National Center for Biotechnology Information (NCBI) Sequence Read Archive (SRA) accession number PRJNA1243451.

## Author contributions

BM, FS, and EM conceived of and designed experiments. BM, AB, FS, LF, SSK, YSC, MTN, NG, CD, LZ, JG, and MBS performed the experiments. BM, FS, LF, AB, SSK, YSC, MTN, JG, MBS, DJM, PPS, FMA, and EM analyzed data. BM, AB, FS, MTN, DJM, JG, MBS, and EM contributed reagents, materials, and/or analysis tools. BM, AB, FS, MTN, YSC, FMA, and EM wrote the paper. All authors have read and agreed to publish the current version of the manuscript.

## Supplementary Material

Supplemental data

Supporting data values

## Figures and Tables

**Table 1:**
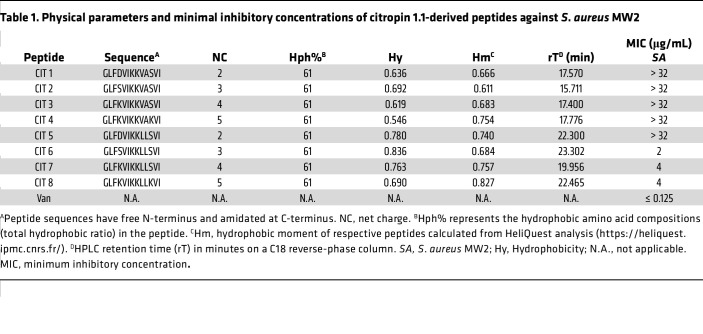
Physical parameters and minimal inhibitory concentrations of citropin 1.1-derived peptides against *S*. *aureus* MW2

**Table 2:**
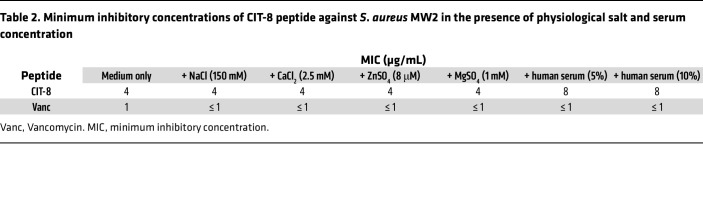
Minimum inhibitory concentrations of CIT-8 peptide against *S*. *aureus* MW2 in the presence of physiological salt and serum concentration

**Table 3:**
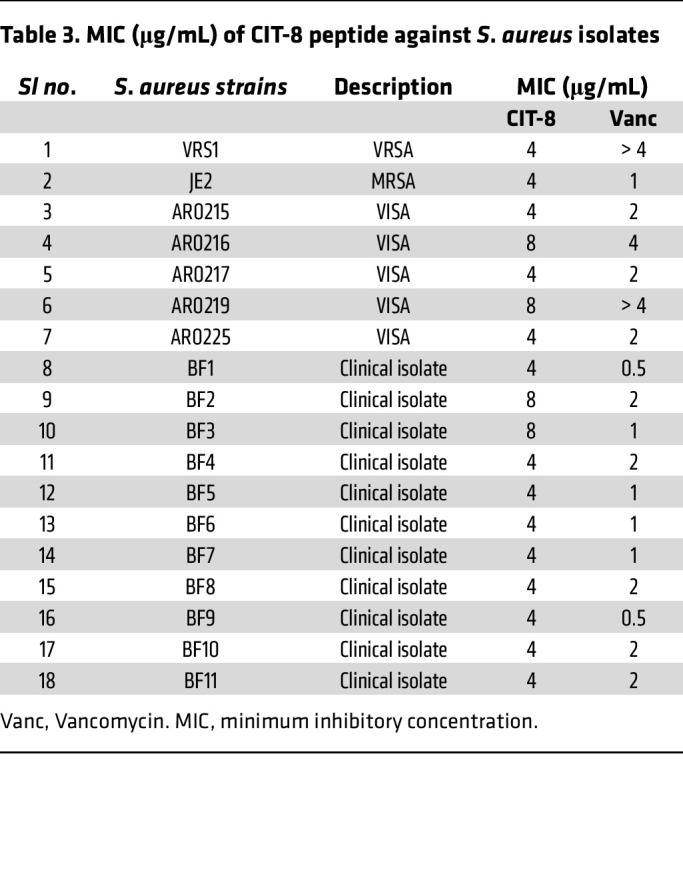
MIC (μg/mL) of CIT-8 peptide against *S*. *aureus* isolates

## Version 1 04/22/2025

In-Press Preview

## Version 2 06/16/2025

Electronic publication
