# Supplementary material for: Antimicrobial peptide developed with machine learning sequence optimization targets drug resistant Staphylococcus aureus in mice
Source: J Clin Invest. 2025 Apr 22;135(12):e185430. doi: 10.1172/JCI185430 (PMC12165799; doi:10.1172/JCI185430)
Supplement: Supplemental data [file jci-135-185430-s164.pdf]

Supplementary materials (SI)

Antimicrobial Peptide Developed with Machine Learning Sequence Optimization Targets Drug Resistant *Staphylococcus aureus* in Mice

Biswajit Mishra[1], Anindya Basu[2,3], Fadi Shehadeh[1,4], LewisOscar Felix[1], Sai Sundeep Kollala[5], Yashpal Singh Chhonker[5], Mandar T. Naik[6], Charilaos Dellis[1], Liyang Zhang[1], Narchonai Ganesan[1], Daryl J. Murry[5], Jianhua Gu[7], Michael B. Sherman[8], Frederick M. Ausubel[9,10], Paul P. Sotiriadis[4,11] and Eleftherios Mylonakis[1*]

[1]Department of Medicine, Houston Methodist Hospital, Houston, Texas, USA

[2]School of Pharmaceutical Sciences and

[3]School of Biomolecular Engineering & Biotechnology, Rajiv Gandhi Technological University, Gandhinagar, Bhopal, Madhya Pradesh, India

[4]Department of Electrical and Computer Engineering, National Technical University of Athens, Athens, Greece

[5]Department of Pharmacy Practice and Science, College of Pharmacy, University of Nebraska Medical Center, Omaha, Nebraska, USA

[6]Department of Molecular Biology, Cell Biology & Biochemistry, Brown University, Providence, Rhode Island, USA

[7]Electron Microscopy Core, Houston Methodist Academic Institute, Houston, Texas, USA

[8]Department of Biochemistry and Molecular Biology, Sealy Center for structural Biology and Molecular Biophysics, The University of Texas Medical Branch at Galveston, Galveston, Texas, USA

[9]Department of Molecular Biology, Massachusetts General Hospital, Boston, Massachusetts, USA
[10]Department of Genetics, Harvard Medical School, Boston, Massachusetts, USA
[11]Archimedes - Athena Research Center, Marousi, Greece
*__Correspondence__: Dr. Eleftherios Mylonakis, Department of Medicine, Houston Methodist Hospital, Houston, Texas, USA (e-mail: emylonakis@houstonmethodist.org)

<u>Contents:</u>

<u>Supplemental results:</u> **Tables- S1-S10**

Figures- S1-S14

<u>Supplemental materials and methods</u>

In silico data collection and processing, bacterial strains growth conditions and peptide synthesis minimal inhibitory concentration (MIC) assay, *S. aureus* persister cell and time-kill assays, biofilm viability assay on solid support, prevention of *S. aureus* MW2 biofilm formation, disruption of *S. aureus* MW2 established biofilms, fluorescence microscopy of CIT-8-treated *S. aureus* MW2 established biofilms, hemolysis of human red blood cells (hRBCs), mammalian cell cytotoxicity assays, dircular dichroism (CD), nuclear magnetic resonance (NMR), molecular dynamics (MD) simulation, membrane depolarization, SYTOX-based cell membrane permeability assay, propidium iodide-based membrane permeability, ATP release assay, cryo-electron microscopy (cryo-EM), scanning electron microscopy (SEM), use of *S. aureus* transposon mutants, RNAseq assays, metabolomic analysis, *in vivo* murine skin infection and treatment.

<u>Supplemental Tables:</u>

<u>Table 1.</u> List of 9 descriptors per peptide sequence.

<u>Table 2.</u> List of the different peptides and properties in the three clusters obtained via the k-mean clustering.

<u>Table 3.</u> Name, amino acid sequences of the template peptides, the final sequences obtained through our ML strategy, and an MIC of the peptides against *S. aureus* MW2.

<u>Table 4.</u> Summary of the NMR structural calculations statistics.

<u>Table 5.</u> Molecular dynamics derived hydrogen bond interactions of CIT-8 with DOPC: DOPG (7:3) model membrane.

<u>Table 6.</u> MIC of CIT-8 against *mprF* transposon mutant from the NTML library.

<u>Table 7.</u> List of RNA-seq derived significant upregulated genes in *S. aureus* MW2 upon CIT-8 interaction at 0.5 × MIC (Cutoff 2-fold).

<u>Table 8.</u> List of RNA-seq derived significant downregulated genes in *S. aureus* MW2 upon CIT-8 interaction at 0.5 × MIC (Cutoff 2-fold).

<u>Table 9.</u> MIC of CIT-8 against Δ*PdxS* transposon mutant from the NTML library in presence of 100 µg/ml vitamin B6.

<u>Table 10.</u> List of bacterial strains used in this study.

Supplemental Figures:

Figure 1. AlphaFold2 structure of CIT-1.

Figure 2.CIT-1 to CIT-8 AlphaFold2 peptide structures and sequences.

Figure 3. Helical wheel plots with hydropathy distribution for peptides CIT-1 to CIT-8

Figure 4. Mammalian toxicity assessment of CIT peptides (A) Hemolysis potential of citropin 1.1-derived peptides on human red blood cells (B) cellular toxicity of CIT-8 to HepG2 cell lines compared with gentamicin control (Genta).

Figure 5. Anti-biofilm and anti-persister activity of CIT-8.

Figure 6. Secondary structure conformation of the peptide CIT-8 in the presence of SDS micelles using circular dichroism.

Figure 7. Change in lipid to surface area ratio upon CIT-8 binding to DOPC: DOPG (7:3) model membrane.

Figure 8. Florescence-based membrane permeation of *S. aureus* MW2 evaluated using (A) PI and (B) SYTOX green.

Figure 9. Pathways downregulated in *S. aureus* MW2 upon CIT-8 interaction at 0.5× MIC (Cutoff 2-fold) identified by RNA-seq.

Figure 10. Partial least squares-discriminant analysis (PLSDA) plots of metabolites between *S. aureus* MW2 control and CIT-8 treated conditions with three technical replicates.

Figure 11. Heatmap representation of significantly altered metabolites in *S. aureus* MW2 bacteria treated with CIT-8.

Figure 12. Experimental repeat of *in vivo* efficacy of CIT-8 in a skin-abraded prophylactic murine model infected with *S. aureus* MW2.

Figure 13. Histopathology of vehicle and CIT-8 treated skin in the skin-abraded prophylactic murine model infected with *S. aureus* MW2.

Supplemental Table 1. List of descriptors per peptide sequence and their values.

Property	mean	std
Mw	2637.053686	2376.57108
gravy	-0.280295413	1.065363567
helicity	0.362555772	0.220982858
hydrophobic_moment100	0.577859595	0.222326721
pcp_descriptors_e1	-0.006978742	0.065195417
pcp_descriptors_e2	-0.022879287	0.10408023
pcp_descriptors_e3	-0.02772609	0.102125427
pcp_descriptors_e4	-0.1137324	0.080133575
pcp_descriptors_e5	-0.00415133	0.082308038
tpsa	1064.112784	999.3103935

Supplemental Table 2. List of the different peptides and properties in the four clusters obtained via the k-mean clustering- attached as a separate excel file.

Supplemental Table 3. Name, amino acid sequences of the template peptides, the final sequences obtained through our ML strategy, and an MIC of the peptides against *S. aureus* MW2.

**Peptide Name**	**Sequence**	***S. aureus*** **MW2 MICs (μg/ml)**
Hylaseptin P1	GIL***D***AIKAIAK***AA***G	> 32
Hylaseptin P1-ML-derived	GILKAIKAIAKLL	2
Mastoparan-L	INLKAL***A***AL***A***KKIL	32
Mastoparan-L-ML derived	LKALKALKKKIL	2
r-CAMEL	LVKL***V***A***G***IKKFLKWK	> 32
r-CAMEL-ML derived	LVKLLALIKKFL	2

The identified residues for substitutions are highlighted in the template peptide sequence using bold, italics, and underlining. In the three selected templates, we identified three substitution positions in the Hylaseptin P1 template, two positions in Mastoparan-L, and two positions in the r-CAMEL template. Substituting two positions with 20 different amino acids in the Hylaseptin P1 template (14-mer) results in 20^3=8,000 possible combinations. Similarly, modifying two positions in Mastoparan-L (14-mer) leads to 20^2=400 combinations, while altering two positions in the r-CAMEL template (15-mer) also yields 20^2=400 possible variants. In a more conservative traditional approach, modifying Hylaseptin P1 by replacing the third-position aspartic acid with either lysine or arginine, and substituting positions 12 and 13 with six key non-polar hydrophobic residues (leucine, phenylalanine, isoleucine, tryptophan, proline, and methionine) results in 72 peptide variants. For Mastoparan L, replacing the two alanines with the six hydrophobic residues generates 36 peptide variants. Similarly, in the r-CAMEL template, substituting valine at position 5 and glycine at position 7 with the six hydrophobic amino acids yields 36 peptide variants. By combining both traditional and machine learning-based approaches, we generated Hylaseptin P1-ML-derived (13-mer), Mastoparan-L-ML-derived (12-mer), and r-CAMEL-ML-derived (12-mer) peptides in a single step by selecting the higher number of AGO instances.

Supplemental Table 4. Summary of the NMR structural calculations statistics. Restraint and validation statistics for CIT-8 structure.

Structural Restraints	
NOE restraints	254
-intra-residue	74
-short-range	81
-medium-range	99
Backbone dihedral angles [a]	22
Hydrogen bonds	14
Validation Summary [b]	
Helical region	residues 2 to 11
-average backbone RMSD to mean	0.23 +/- 0.14 A
-average heavy atom RMSD to mean	0.37 +/- 0.11 A
NOE-derived distance violations >0.1 Å	2
dihedral angle violations >5°	0
CYANA target function	0.76
Ramachandran plot statistics	
-most favored region (%)	100
-additionally allowed region (%)	0
-generously allowed region (%)	0

[a] Predicted by the TALOS+ software
[b] Calculated by PROCHECK 3.2

Supplemental Table 5. Molecular dynamics derived hydrogen bond interactions of CIT-8 with DOPC: DOPG (7:3) model membrane.

With nearest DOPC | With nearest DOPG

DONOR	ACCEPTOR	OCCUPANCY	DONOR	ACCEPTOR	OCCUPANCY
LEU2-Side	DOPC1-Side	0.99%	LEU10-Side	DOPG5-Side	0.49%
VAL5-Side	DOPC1-Side	0.49%	VAL12-Side	DOPG6-Side	0.49%
VAL5-Main	DOPC1-Side	0.49%	LYS8-Main	DOPG2-Side	0.49%
LEU2-Main	DOPC1-Side	0.49%	LYS11-Side	DOPG2-Side	22.66%
PHE3-Main	DOPC1-Side	0.49%	LYS7-Side	DOPG2-Side	1.97%
LYS4-Main	DOPC1-Side	0.49%	LYS8-Side	DOPG2-Side	82.76%
DOPC1-Side	LEU2-Side	0.49%	DOPG6-Side	VAL12-Main	0.99%
DOPC1-Side	LEU2-Main	0.49%	LYS11-Side	DOPG5-Side	56.65%
LYS7-Side	DOPC1-Side	31.53%	PHE3-Side	DOPG4-Side	1.97%
GLY1-Main	DOPC3-Side	3.45%	LYS11-Side	DOPG4-Side	2.46%
GLY1-Main	DOPC1-Side	6.40%	LYS4-Side	DOPG7-Side	62.07%
PHE3-Side	DOPC1-Side	1.48%	DOPG5-Side	LEU10-Main	8.37%
DOPC1-Side	GLY1-Main	0.49%	LYS7-Side	DOPG4-Side	47.29%
DOPC12-Side	VAL12-Main	0.99%	DOPG5-Side	VAL12-Main	0.49%
LEU2-Main	DOPC3-Side	0.49%	DOPG5-Side	LEU9-Main	2.46%
			DOPG6-Side	LYS11-Main	0.49%
			GLY1-Main	DOPG7-Side	1.48%
			LYS4-Side	DOPG2-Side	43.35%
			DOPG2-Side	ILE13-Side	0.99%
			ILE13-Side	DOPG2-Side	9.85%
			DOPG7-Side	GLY1-Main	2.46%
			DOPG2-Side	VAL12-Main	1.48%
			LYS8-Side	DOPG5-Side	0.49%
			DOPG2-Side	ILE13-Main	0.99%
			DOPG5-Side	LYS11-Main	12.32%

Supplemental Table 6. MIC of CIT-8 against *mprF* transposon mutant from the NTML library.

**Strain description**	**MIC (µg/ml)**
mprF transposon mutant	2
JE2	4

Supplemental Table 7. List of RNA-seq derived significant upregulated genes in *S. aureus* MW2 upon CIT-8 interaction at 0.5× MIC (Cutoff 2-fold).

gene_id	log2Fold Change	gene_description
MW_RS13100	5.925659	hypothetical protein && -
MW_RS02625	5.73888	pyridoxal 5'-phosphate synthase lyase subunit PdxS && PF01680:SOR/SNZ family
MW_RS02630	5.352432	pyridoxal 5'-phosphate synthase glutaminase subunit PdxT && PF01174:SNO glutamine amidotransferase family
MW_RS12365	5.293872	ABC transporter permease && PF02687:FtsX-like permease family|PF12704:MacB-like periplasmic core domain
MW_RS12465	5.218527	sucrose-specific PTS transporter subunit IIBC && PF02378:Phosphotransferase system, EIIC|PF00367:phosphotransferase system, EIIB
MW_RS05115	5.208643	phosphoribosylformylglycinamidine synthase subunit PurL && PF00586:AIR synthase related protein, N-terminal domain|PF02769:AIR synthase related protein, C-terminal domain
MW_RS05105	5.06384	phosphoribosylformylglycinamidine synthase subunit PurS && PF02700:Phosphoribosylformylglycinamidine (FGAM) synthase
MW_RS05110	5.003102	phosphoribosylformylglycinamidine synthase I && PF13507:CobB/CobQ-like glutamine amidotransferase domain
MW_RS14285	4.822027	signal recognition particle sRNA large type && -
MW_RS05120	4.780343	amidophosphoribosyltransferase && PF00156:Phosphoribosyl transferase domain|PF13537:Glutamine amidotransferase domain
MW_RS12360	4.735917	ABC transporter ATP-binding protein && PF07673:Protein of unknown function (DUF1602)|PF00005:ABC transporter
MW_RS11260	4.712888	SDR family oxidoreductase && PF13460:NAD(P)H-binding
MW_RS05125	4.684106	phosphoribosylformylglycinamidine cyclo-ligase && PF00586:AIR synthase related protein, N-terminal domain|PF02769:AIR synthase related protein, C-terminal domain
MW_RS06935	4.268117	acylphosphatase && PF00708:Acylphosphatase
MW_RS03885	4.177316	ribosome-associated translation inhibitor RaiA && PF02482:Sigma 54 modulation protein / S30EA ribosomal protein|PF16321:Sigma 54 modulation/S30EA ribosomal protein C terminus
MW_RS05895	4.174026	TM2 domain-containing protein && PF05154:TM2 domain
MW_RS05130	4.157564	phosphoribosylglycinamide formyltransferase && PF00551:Formyl transferase
MW_RS14200	4.124403	HdeD family acid-resistance protein && PF03729:Short repeat of unknown function (DUF308)
MW_RS14300	4.098962	6S RNA && -

MW_RS12215	4.061072	urocanate hydratase && PF01175:Urocanase
MW_RS13590	3.913284	fructosamine kinase family protein && PF03881:Fructosamine kinase
MW_RS12025	3.65518	PH domain-containing protein && PF14470:Bacterial PH domain
MW_RS05135	3.632262	bifunctional phosphoribosylaminoimidazolecarboxamide formyltransferase/IMP cyclohydrolase && PF01808:AICARFT/IMPCHase bienzyme|PF02142:MGS-like domain
MW_RS08960	3.608883	acetate--CoA ligase && PF13193:AMP-binding enzyme C-terminal domain|PF00501:AMP-binding enzyme
MW_RS12225	3.574131	formimidoylglutamase && PF00491:Arginase family
MW_RS08585	3.561791	hypothetical protein && -
MW_RS08805	3.550333	metal-dependent hydrolase && PF13483:Beta-lactamase superfamily domain
MW_RS03995	3.455018	DUF4887 domain-containing protein && PF16228:Domain of unknown function (DUF4887)
MW_RS13895	3.376541	immunodominant staphylococcal antigen IsaB && -
MW_RS05320	3.358611	DUF5325 family protein && -
MW_RS02925	3.32713	C1q-binding complement inhibitor VraX && -
MW_RS05100	3.30955	phosphoribosylaminoimidazolesuccinocarboxamide synthase && PF01259:SAICAR synthetase
MW_RS14485	3.289163	phenol-soluble modulin PSM-alpha-3 && -
MW_RS05140	3.244946	phosphoribosylamine--glycine ligase && PF02844:Phosphoribosylglycinamide synthetase, N domain|PF02843:Phosphoribosylglycinamide synthetase, C domain|PF01071:Phosphoribosylglycinamide synthetase, ATP-grasp (A) domain
MW_RS09390	3.235221	lantibiotic immunity ABC transporter MutE/EpiE family permease subunit && PF12730:ABC-2 family transporter protein
MW_RS12510	3.215394	NarK/NasA family nitrate transporter && PF07690:Major Facilitator Superfamily
MW_RS06365	3.151603	glutathione peroxidase && PF00255:Glutathione peroxidase
MW_RS10165	3.150579	hypothetical protein && -
MW_RS01105	3.015525	acyl CoA:acetate/3-ketoacid CoA transferase && PF01144:Coenzyme A transferase
MW_RS07250	2.960175	zinc metallopeptidase && PF04298:Putative neutral zinc metallopeptidase
MW_RS11310	2.954519	PTS sugar transporter subunit IIA && PF00359:Phosphoenolpyruvate-dependent sugar phosphotransferase system, EIIA 2
MW_RS00385	2.949644	tandem-type lipoprotein && PF04507:Protein of unknown function, DUF576
MW_RS09395	2.947322	lantibiotic protection ABC transporter ATP-binding subunit && PF00005:ABC transporter
MW_RS12425	2.940317	GNAT family N-acetyltransferase && PF13508:Acetyltransferase (GNAT) domain
MW_RS13705	2.890641	antibiotic biosynthesis monooxygenase && PF03992:Antibiotic biosynthesis monooxygenase
MW_RS05300	2.847777	DUF4064 domain-containing protein && PF13273:Protein of unknown function (DUF4064)
MW_RS01805	2.846272	30S ribosomal protein S18 && PF01084:Ribosomal protein S18
MW_RS15240	2.831412	hypothetical protein && -

MW_RS14165	2.830722	bacillithiol transferase BstA && -
MW_RS03335	2.825148	dihydroxyacetone kinase subunit L && PF02734:DAK2 domain
MW_RS03805	2.80864	siderophore ABC transporter substrate-binding protein && PF01497:Periplasmic binding protein
MW_RS08740	2.807694	NADP-dependent isocitrate dehydrogenase && PF00180:Isocitrate/isopropylmalate dehydrogenase
MW_RS13610	2.799715	glyoxalase/bleomycin resistance/extradiol dioxygenase family protein && PF06983:3-demethylubiquinone-9 3-methyltransferase
MW_RS04215	2.78385	hypothetical protein && -
MW_RS10700	2.770566	ammonium transporter && PF00909:Ammonium Transporter Family
MW_RS12995	2.735856	SDR family oxidoreductase && PF00106:short chain dehydrogenase
MW_RS11480	2.728283	Asp23/Gls24 family envelope stress response protein && PF03780:Asp23 family, cell envelope-related function
MW_RS12470	2.720228	YbgA family protein && PF08349:Protein of unknown function (DUF1722)
MW_RS12905	2.712514	ATP-binding cassette domain-containing protein && PF00005:ABC transporter|PF07673:Protein of unknown function (DUF1602)
MW_RS06850	2.708572	phosphate ABC transporter substrate-binding protein PstS && PF12849:PBP superfamily domain
MW_RS12990	2.684583	hypothetical protein && -
MW_RS06715	2.672442	4-oxalocrotonate tautomerase && PF01361:Tautomerase enzyme
MW_RS01100	2.660759	acyl--CoA ligase && PF13193:AMP-binding enzyme C-terminal domain|PF00501:AMP-binding enzyme
MW_RS08030	2.659205	rhomboid family intramembrane serine protease && PF01694:Rhomboid family
MW_RS13035	2.652385	SDR family oxidoreductase && PF00106:short chain dehydrogenase
MW_RS13635	2.651061	aspartate 1-decarboxylase && PF02261:Aspartate decarboxylase
MW_RS13040	2.626166	single-stranded DNA-binding protein && PF07205:Domain of unknown function (DUF1413)
MW_RS04035	2.613654	preprotein translocase subunit SecG && PF03840:Preprotein translocase SecG subunit
MW_RS03505	2.606465	hypothetical protein && -
MW_RS13580	2.594598	hypothetical protein && -
MW_RS08745	2.59139	citrate synthase && PF00285:Citrate synthase
MW_RS08105	2.590293	helix-turn-helix transcriptional regulator && PF00571:CBS domain|PF08279:HTH domain
MW_RS05705	2.585268	N-acetyltransferase && -
MW_RS11490	2.584342	alkaline shock response membrane anchor protein AmaP && -
MW_RS01840	2.582306	GlsB/YeaQ/YmgE family stress response membrane protein && PF04226:Transglycosylase associated protein
MW_RS00375	2.582222	tandem-type lipoprotein && PF04507:Protein of unknown function, DUF576
MW_RS08475	2.570187	preprotein translocase subunit YajC && PF02699:Preprotein translocase subunit
MW_RS05090	2.567763	5-(carboxyamino)imidazole ribonucleotide mutase && PF00731:AIR carboxylase
MW_RS10780	2.546807	2-isopropylmalate synthase && PF00682:HMGL-like|PF08502:LeuA allosteric (dimerisation) domain

MW_RS08825	2.544992	universal stress protein && PF00582:Universal stress protein family
MW_RS03220	2.537655	Na+/H+ antiporter Mnh2 subunit F && PF04066:Multiple resistance and pH regulation protein F (MrpF / PhaF)
MW_RS01025	2.534	hexose-6-phosphate:phosphate antiporter && PF07690:Major Facilitator Superfamily
MW_RS13680	2.520127	hypothetical protein && -
MW_RS03615	2.519425	aldo/keto reductase && PF00248:Aldo/keto reductase family
MW_RS02165	2.51612	DUF2294 domain-containing protein && PF10057:Uncharacterized conserved protein (DUF2294)
MW_RS11340	2.515277	arginase && PF00491:Arginase family
MW_RS08225	2.507203	ComE operon protein 2 && PF00383:Cytidine and deoxycytidylate deaminase zinc-binding region
MW_RS12900	2.487598	iron export ABC transporter permease subunit FetB && PF03649:Uncharacterised protein family (UPF0014)
MW_RS03285	2.481998	glycerol-3-phosphate cytidylyltransferase && PF01467:Cytidylyltransferase-like
MW_RS09920	2.472839	YtxH domain-containing protein && -
MW_RS03570	2.470043	multidrug efflux MFS transporter NorA && PF07690:Major Facilitator Superfamily
MW_RS00130	2.461477	23S rRNA (pseudouridine(1915)-N(3))-methyltransferase RlmH && PF02590:Predicted SPOUT methyltransferase
MW_RS06975	2.459224	DUF6501 family protein && -
MW_RS11305	2.451945	BglG family transcription antiterminator && PF00874:PRD domain|PF02302:PTS system, Lactose/Cellobiose specific IIB subunit|PF05043:Mga helix-turn-helix domain|PF00359:Phosphoenolpyruvate-dependent sugar phosphotransferase system, EIIA 2|PF08279:HTH domain
MW_RS11300	2.447446	PTS mannitol transporter subunit IICB && PF02302:PTS system, Lactose/Cellobiose specific IIB subunit|PF02378:Phosphotransferase system, EIIC
MW_RS10950	2.441358	hypothetical protein && -
MW_RS04735	2.441044	adaptor protein MecA && PF05389:Negative regulator of genetic competence (MecA)
MW_RS02140	2.41201	hypothetical protein && -
MW_RS11510	2.408461	alpha/beta hydrolase && PF06028:Alpha/beta hydrolase of unknown function (DUF915)
MW_RS02800	2.402756	NAD-dependent epimerase/dehydratase family protein && PF01370:NAD dependent epimerase/dehydratase family
MW_RS04760	2.386468	CYTH domain-containing protein && PF01928:CYTH domain
MW_RS13225	2.38577	ring-cleaving dioxygenase && PF00903:Glyoxalase/Bleomycin resistance protein/Dioxygenase superfamily
MW_RS00755	2.37576	cation diffusion facilitator family transporter && PF01545:Cation efflux family
MW_RS10800	2.372959	threonine ammonia-lyase IlvA && PF00585:C-terminal regulatory domain of Threonine dehydratase|PF00291:Pyridoxal-phosphate dependent enzyme
MW_RS13160	2.357153	GntR family transcriptional regulator && PF00392:Bacterial regulatory proteins, gntR family|PF07729:FCD domain

MW_RS06840	2.348875	phosphate ABC transporter permease PstA && PF00528:Binding-protein-dependent transport system inner membrane component
MW_RS07090	2.347402	zinc-finger domain-containing protein && PF10782:Protein of unknown function (DUF2602)
MW_RS03330	2.34432	dihydroxyacetone kinase subunit DhaK && PF02733:Dak1 domain
MW_RS10090	2.343819	NETI motif-containing protein && PF14044:NETI protein
MW_RS01585	2.343367	LLM class flavin-dependent oxidoreductase && PF00296:Luciferase-like monooxygenase
MW_RS08300	2.342882	divalent metal cation transporter && PF01566:Natural resistance-associated macrophage protein
MW_RS07045	2.336228	PTS glucose transporter subunit IIA && PF00358:phosphoenolpyruvate-dependent sugar phosphotransferase system, EIIA 1
MW_RS03225	2.332274	Na+/H+ antiporter Mnh2 subunit G && PF03334:Na+/H+ antiporter subunit
MW_RS12540	2.325173	respiratory nitrate reductase subunit gamma && PF02665:Nitrate reductase gamma subunit
MW_RS02425	2.302198	septation regulator SpoVG && PF04026:SpoVG
MW_RS06630	2.294443	large conductance mechanosensitive channel protein MscL && PF01741:Large-conductance mechanosensitive channel, MscL
MW_RS09000	2.288733	DUF948 domain-containing protein && PF06103:Bacterial protein of unknown function (DUF948)
MW_RS08865	2.285232	septation ring formation regulator EzrA && PF06160:Septation ring formation regulator, EzrA
MW_RS11285	2.283743	Cof-type HAD-IIB family hydrolase && PF08282:haloacid dehalogenase-like hydrolase
MW_RS02640	2.283348	CtsR family transcriptional regulator && PF05848:Firmicute transcriptional repressor of class III stress genes (CtsR)
MW_RS10005	2.274665	DNA polymerase IV && PF00817:impB/mucB/samB family|PF11799:impB/mucB/samB family C-terminal domain
MW_RS06980	2.273592	hypothetical protein && -
MW_RS07175	2.263934	cell division regulator GpsB && PF05103:DivIVA protein
MW_RS14305	2.258034	hypothetical protein && -
MW_RS03165	2.251268	alpha/beta hydrolase && PF00561:alpha/beta hydrolase fold
MW_RS06845	2.2508	phosphate ABC transporter permease subunit PstC && PF00528:Binding-protein-dependent transport system inner membrane component
MW_RS00015	2.248065	S4 domain-containing protein YaaA && PF13275:S4 domain
MW_RS10685	2.239649	carbohydrate kinase && PF00294:pfkB family carbohydrate kinase
MW_RS11315	2.235732	mannitol-1-phosphate 5-dehydrogenase && PF08125:Mannitol dehydrogenase C-terminal domain|PF01232:Mannitol dehydrogenase Rossmann domain
MW_RS04260	2.234784	glycine cleavage system protein GcvH && PF01597:Glycine cleavage H-protein
MW_RS11850	2.229494	hypothetical protein && -
MW_RS01665	2.223598	NAD(P)H-dependent oxidoreductase && PF03358:NADPH-dependent FMN reductase

MW_RS12720	2.220888	biotin synthase BioB && PF06968:Biotin and Thiamin Synthesis associated domain|PF04055:Radical SAM superfamily
MW_RS06060	2.216858	ADP-forming succinate--CoA ligase subunit beta && PF00549:CoA-ligase|PF08442:ATP-grasp domain
MW_RS04155	2.203929	MSCRAMM family adhesin clumping factor ClfA && PF04650:YSIRK type signal peptide|PF10425:C-terminus of bacterial fibrinogen-binding adhesin
MW_RS07840	2.192932	tripeptidase T && PF07687:Peptidase dimerisation domain|PF01546:Peptidase family M20/M25/M40
MW_RS01595	2.18039	protein-ADP-ribose hydrolase && PF01661:Macro domain
MW_RS11485	2.169264	DUF2273 domain-containing protein && PF10031:Small integral membrane protein (DUF2273)
MW_RS01095	2.157744	acyl-CoA dehydrogenase family protein && PF00441:Acyl-CoA dehydrogenase, C-terminal domain|PF02771:Acyl-CoA dehydrogenase, N-terminal domain|PF02770:Acyl-CoA dehydrogenase, middle domain
MW_RS05180	2.155245	hypothetical protein && -
MW_RS11020	2.152301	YwpF-like family protein && PF14183:YwpF-like protein
MW_RS09115	2.14969	MarR family transcriptional regulator && PF01047:MarR family
MW_RS12060	2.149147	DUF4870 domain-containing protein && PF09685:Domain of unknown function (DUF4870)
MW_RS02115	2.147476	tandem-type lipoprotein && PF04507:Protein of unknown function, DUF576
MW_RS03155	2.136646	hypothetical protein && -
MW_RS06540	2.13101	hypothetical protein && -
MW_RS03620	2.114641	lipoteichoic acid-specific glycosylation protein CsbB && PF00535:Glycosyl transferase family 2
MW_RS00140	2.112095	hypothetical protein && -
MW_RS10790	2.111605	3-isopropylmalate dehydratase large subunit && PF00330:Aconitase family (aconitate hydratase)
MW_RS09575	2.111483	YlbF/YmcA family competence regulator && PF06133:Control of competence regulator ComK, YlbF/YmcA
MW_RS07970	2.107863	shikimate kinase && PF01202:Shikimate kinase
MW_RS05530	2.100985	succinate dehydrogenase cytochrome b558 subunit && PF01127:Succinate dehydrogenase/Fumarate reductase transmembrane subunit
MW_RS11505	2.092309	NADP-dependent oxidoreductase && PF00107:Zinc-binding dehydrogenase|PF16884:N-terminal domain of oxidoreductase
MW_RS12760	2.088003	type I toxin-antitoxin system Fst family toxin && -
MW_RS05165	2.086477	glycopeptide resistance-associated protein GraF && -
MW_RS04595	2.075651	YisL family protein && PF07457:Protein of unknown function (DUF1516)
MW_RS01725	2.075079	ABC-2 transporter permease && PF13346:ABC-2 family transporter protein
MW_RS09130	2.072816	proline dehydrogenase && PF01619:Proline dehydrogenase
MW_RS13220	2.070222	alpha/beta hydrolase && PF12695:Alpha/beta hydrolase family
MW_RS09400	2.068391	S8 family serine peptidase && PF00082:Subtilase family

MW_RS01505	2.066927	ABC transporter permease && PF02687:FtsX-like permease family|PF12704:MacB-like periplasmic core domain
MW_RS08705	2.062211	dephospho-CoA kinase && PF01121:Dephospho-CoA kinase
MW_RS06875	2.058965	aspartate kinase && PF00696:Amino acid kinase family
MW_RS13340	2.057541	LrgB family protein && PF04172:LrgB-like family
MW_RS09890	2.049439	hypothetical protein && -
MW_RS00750	2.046237	aldehyde dehydrogenase family protein && PF00171:Aldehyde dehydrogenase family
MW_RS08410	2.044402	CsbD family protein && PF05532:CsbD-like
MW_RS11225	2.029617	deoxyribose-phosphate aldolase && PF01791:DeoC/LacD family aldolase
MW_RS01615	2.028616	PTS ascorbate transporter subunit IIC && PF03611:PTS system sugar-specific permease component
MW_RS03970	2.028341	YvcK family protein && PF01933:Uncharacterised protein family UPF0052
MW_RS06390	2.024441	hypothetical protein && -
MW_RS13355	2.017273	sterile alpha motif-like domain-containing protein && PF06855:YozE SAM-like fold
MW_RS08580	2.016528	AbrB family transcriptional regulator && PF05145:Putative ammonia monooxygenase
MW_RS12545	2.011424	nitrate reductase molybdenum cofactor assembly chaperone && -
MW_RS03985	2.008353	ATP-dependent Clp endopeptidase proteolytic subunit ClpP && PF00574:Clp protease
MW_RS09540	2.002486	YtxH domain-containing protein && -
MW_RS04530	2.002136	NADH-dependent flavin oxidoreductase && PF00724:NADH:flavin oxidoreductase / NADH oxidase family
MW_RS09250	2.00129	membrane protein insertion efficiency factor YidD && PF01809:Haemolytic domain

Supplemental Table 8. List of RNA-seq derived significant downregulated genes in *S. aureus* MW2 upon CIT-8 interaction at 0.5× MIC (Cutoff 2-fold).

gene_id	log2 FoldChange	gene_description
MW_RS09670	-8.536468609	tRNA-Gln && -
MW_RS09710	-8.47875053	tRNA-Ser && -
MW_RS12590	-7.913399685	hypothetical protein && -
MW_RS05685	-7.713649959	tRNA-Arg && -
MW_RS09665	-6.899900398	tRNA-Cys && -
MW_RS02265	-6.893053618	tRNA-Ser && -
MW_RS02595	-6.553413925	5S ribosomal RNA && -
MW_RS09760	-5.921874566	tRNA-Leu && -
MW_RS04175	-5.914867396	thermonuclease family protein && PF00565:Staphylococcal nuclease homologue
MW_RS09970	-5.756983131	hypothetical protein && -
MW_RS04210	-5.540960177	sterile alpha motif-like domain-containing protein && PF06855:YozE SAM-like fold
MW_RS09735	-5.510307736	tRNA-Ala && -
MW_RS02555	-5.485399319	tRNA-Lys && -
MW_RS09720	-5.479911513	tRNA-Ser && -
MW_RS01495	-5.239345952	5'-nucleotidase%2C lipoprotein e(P4) family && PF03767:HAD superfamily, subfamily IIIB (Acid phosphatase)
MW_RS14970	-5.218780827	hypothetical protein && -
MW_RS15045	-5.179641086	helix-turn-helix domain-containing protein && PF01527:Transposase
MW_RS09655	-5.071958479	tRNA-Leu && -
MW_RS13120	-4.931129514	HTH-type transcriptional regulator SarU && -
MW_RS04935	-4.928406872	hypothetical protein && -
MW_RS09725	-4.917959069	tRNA-Ile && -
MW_RS14860	-4.859985523	site-specific integrase && PF00589:Phage integrase family
MW_RS02540	-4.799489515	5S ribosomal RNA && -
MW_RS07135	-4.758226368	alanine dehydrogenase && PF01262:Alanine dehydrogenase/PNT, C-terminal domain|PF05222:Alanine dehydrogenase/PNT, N-terminal domain
MW_RS03305	-4.749920747	hypothetical protein && -
MW_RS13745	-4.728241127	hypothetical protein && -
MW_RS13750	-4.678678594	hypothetical protein && -
MW_RS01980	-4.615537375	hypothetical protein && -
MW_RS01965	-4.614988368	hypothetical protein && -
MW_RS03245	-4.582878672	metal ABC transporter ATP-binding protein && PF00005:ABC transporter
MW_RS14480	-4.523055603	MafB-like protein && PF15534:Bacterial toxin 35
MW_RS09685	-4.516640838	tRNA-Tyr && -
MW_RS03660	-4.384857867	7-cyano-7-deazaguanine synthase QueC && PF06508:Queuosine biosynthesis protein QueC
MW_RS04170	-4.297048814	hypothetical protein && -
MW_RS13755	-4.163593283	anaerobic ribonucleoside-triphosphate reductase activating protein && PF04055:Radical SAM superfamily|PF13353:4Fe-4S single cluster domain
MW_RS12985	-4.151359997	histidine racemase CntK && -

MW_RS14685	-4.144393148	minor capsid protein && PF15542:Bacterial toxin 50|PF04233:Phage Mu protein F like protein
MW_RS01120	-4.136356429	DUF488 domain-containing protein && PF04343:Protein of unknown function, DUF488
MW_RS04875	-4.070486131	competence protein ComK && PF06338:ComK protein
MW_RS07125	-4.056769264	amino acid permease && PF13520:Amino acid permease
MW_RS07150	-3.989867002	PepSY domain-containing protein && PF03929:PepSY-associated TM region
MW_RS07500	-3.884268906	hypothetical protein && -
MW_RS12660	-3.8378672	putative metal homeostasis protein && -
MW_RS07120	-3.835772803	multidrug efflux MFS transporter NorB && PF07690:Major Facilitator Superfamily
MW_RS12980	-3.793844686	D-histidine (S)-2-aminobutanoyltransferase CntL && -
MW_RS07515	-3.781480658	phage major capsid protein && PF05065:Phage capsid family
MW_RS09730	-3.778694263	tRNA-Met && -
MW_RS10040	-3.772788146	Asp-tRNA(Asn)/Glu-tRNA(Gln) amidotransferase subunit GatC && PF02686:Glu-tRNAGln amidotransferase C subunit
MW_RS06590	-3.763461518	hypothetical protein && -
MW_RS09690	-3.73529537	tRNA-Thr && -
MW_RS07540	-3.711930275	HNH endonuclease && PF01844:HNH endonuclease
MW_RS00295	-3.693311358	persulfide response sulfurtransferase CstA && PF00581:Rhodanese-like domain|PF01206:Sulfurtransferase TusA|PF13686:DsrE/DsrF/DrsH-like family
MW_RS11605	-3.675676171	acetolactate synthase AlsS && PF00205:Thiamine pyrophosphate enzyme, central domain|PF02775:Thiamine pyrophosphate enzyme, C-terminal TPP binding domain|PF02776:Thiamine pyrophosphate enzyme, N-terminal TPP binding domain
MW_RS00975	-3.668299447	FMN-dependent NADH-azoreductase && PF02525:Flavodoxin-like fold
MW_RS15225	-3.654656679	minor capsid protein && PF04233:Phage Mu protein F like protein
MW_RS09265	-3.615080768	DUF4909 domain-containing protein && PF16253:Domain of unknown function (DUF4909)
MW_RS13570	-3.613498518	NAD(P)-binding domain-containing protein && PF13738:Pyridine nucleotide-disulphide oxidoreductase
MW_RS01020	-3.601338834	isoprenylcysteine carboxyl methyltransferase family protein && PF04140:Isoprenylcysteine carboxyl methyltransferase (ICMT) family
MW_RS10520	-3.579708808	hypothetical protein && -
MW_RS10405	-3.535332366	phage terminase small subunit P27 family && PF05119:Phage terminase, small subunit
MW_RS07130	-3.533733328	bifunctional threonine ammonia-lyase/L-serine ammonia-lyase TdcB && PF00291:Pyridoxal-phosphate dependent enzyme
MW_RS12975	-3.507577973	staphylopine dehydrogenase CntM && PF10100:Uncharacterized protein conserved in bacteria (DUF2338)
MW_RS10825	-3.499606698	tRNA-Leu && -
MW_RS05625	-3.451089892	alpha-hemolysin && PF07968:Leukocidin/Hemolysin toxin family
MW_RS09715	-3.433346999	tRNA-Asp && -
MW_RS10295	-3.413107132	hypothetical protein && -
MW_RS04400	-3.40211143	hypothetical protein && -
MW_RS10665	-3.397306898	cyclic lactone autoinducer peptide && PF05931:Staphylococcal AgrD protein
MW_RS08635	-3.377438557	hypothetical protein && -
MW_RS13760	-3.373198152	anaerobic ribonucleoside-triphosphate reductase && PF13597:Anaerobic ribonucleoside-triphosphate reductase
MW_RS09675	-3.370020554	tRNA-His && -
MW_RS10355	-3.368593603	hypothetical protein && -
MW_RS10460	-3.364166339	hypothetical protein && -
MW_RS10340	-3.341617529	hypothetical protein && -
MW_RS07435	-3.32651081	XkdX family protein && PF09693:Phage uncharacterised protein (Phage_XkdX)
MW_RS09680	-3.313970838	tRNA-Trp && -

MW_RS03820	-3.294701088	GrpB family protein && PF04229:GrpB protein
MW_RS03650	-3.272034708	7-carboxy-7-deazaguanine synthase QueE && PF04055:Radical SAM superfamily|PF13353:4Fe-4S single cluster domain
MW_RS07510	-3.261681626	head-tail connector protein && PF05135:Phage gp6-like head-tail connector protein
MW_RS04630	-3.2539921	LeuA family protein && PF00682:HMGL-like
MW_RS00035	-3.248870089	NAD(P)H-hydrate dehydratase && PF01256:Carbohydrate kinase
MW_RS10415	-3.237073609	nucleoside triphosphate pyrophosphohydrolase family protein && -
MW_RS13130	-3.236043227	fibronectin-binding protein FnbB && PF02986:Fibronectin binding repeat|PF04650:YSIRK type signal peptide|PF00746:Gram positive anchor|PF10425:C-terminus of bacterial fibrinogen-binding adhesin
MW_RS07375	-3.203670506	ferredoxin && PF13370:4Fe-4S single cluster domain of Ferredoxin I
MW_RS07505	-3.197387967	hypothetical protein && -
MW_RS10285	-3.189831431	CHAP domain-containing protein && PF05257:CHAP domain
MW_RS10290	-3.186608149	phage holin && PF04531:Bacteriophage holin
MW_RS10395	-3.18244463	hypothetical protein && -
MW_RS07420	-3.180608252	N-acetylmuramoyl-L-alanine amidase && PF01520:N-acetylmuramoyl-L-alanine amidase|PF08460:Bacterial SH3 domain|PF05257:CHAP domain
MW_RS09450	-3.162022307	tRNA-Ser && -
MW_RS12815	-3.152950414	APC family permease && PF13520:Amino acid permease
MW_RS10160	-3.137622168	YolD-like family protein && PF08863:YolD-like protein
MW_RS07520	-3.112673835	Clp protease ClpP && PF00574:Clp protease
MW_RS07495	-3.0833881	DUF3168 domain-containing protein && PF11367:Protein of unknown function (DUF3168)
MW_RS10475	-3.077843193	MBL fold metallo-hydrolase && PF12706:Beta-lactamase superfamily domain
MW_RS04195	-3.067570251	hypothetical protein && -
MW_RS09370	-3.047358681	serine protease SplA && PF00089:Trypsin
MW_RS07410	-3.042611901	Panton-Valentine bi-component leukocidin subunit F && PF07968:Leukocidin/Hemolysin toxin family
MW_RS11985	-3.038885745	urease subunit beta && PF00699:Urease beta subunit
MW_RS07445	-3.020530296	BppU family phage baseplate upper protein && PF10651:Domain of unknown function (DUF2479)
MW_RS07530	-3.016517807	terminase large subunit && PF03354:Phage Terminase
MW_RS01115	-3.005415341	ABC transporter substrate-binding protein && PF00496:Bacterial extracellular solute-binding proteins, family 5 Middle
MW_RS09360	-3.004247691	serine protease SplC && PF00089:Trypsin
MW_RS14710	-2.997314928	transposase && PF13751:Transposase DDE domain
MW_RS04075	-2.9877542	hypothetical protein && -
MW_RS07525	-2.987746953	phage portal protein && PF04860:Phage portal protein
MW_RS13970	-2.97980745	accessory Sec system protein translocase subunit SecY2 && PF00344:SecY translocase
MW_RS03655	-2.923399656	6-carboxytetrahydropterin synthase QueD && PF01242:6-pyruvoyl tetrahydropterin synthase
MW_RS15050	-2.923261275	exotoxin && PF02876:Staphylococcal/Streptococcal toxin, beta-grasp domain
MW_RS01835	-2.897099969	helix-turn-helix domain-containing protein && -
MW_RS10500	-2.894931657	DUF1270 domain-containing protein && PF06900:Protein of unknown function (DUF1270)
MW_RS10270	-2.885530416	SH3 domain-containing protein && PF08460:Bacterial SH3 domain
MW_RS14220	-2.871041264	cold-shock protein && PF00313:'Cold-shock' DNA-binding domain
MW_RS13630	-2.865528545	LPXTG-anchored surface protein SasK && -
MW_RS00235	-2.861981317	persulfide dioxygenase-sulfurtransferase CstB && PF00581:Rhodanese-like domain
MW_RS12180	-2.855031517	MurR/RpiR family transcriptional regulator && PF01380:SIS domain|PF01418:Helix-turn-helix domain, rpiR family

MW_RS07415	-2.852647251	Panton-Valentine bi-component leukocidin subunit S && PF07968:Leukocidin/Hemolysin toxin family
MW_RS12065	-2.849323779	CHAP domain-containing protein && PF05257:CHAP domain
MW_RS07460	-2.83432554	phage tail protein && PF06605:Prophage endopeptidase tail
MW_RS09570	-2.832299638	hypothetical protein && -
MW_RS13360	-2.831835103	CHAP domain-containing protein && PF05257:CHAP domain
MW_RS07490	-2.827829641	tail protein && PF04630:Phage tail tube protein
MW_RS09585	-2.804936985	helix-turn-helix transcriptional regulator && -
MW_RS09380	-2.802784794	DUF4888 domain-containing protein && PF16229:Domain of unknown function (DUF4888)
MW_RS12325	-2.798801608	TetR/AcrR family transcriptional regulator && PF00440:Bacterial regulatory proteins, tetR family
MW_RS14780	-2.795998206	transposase && PF01610:Transposase
MW_RS07535	-2.78317993	P27 family phage terminase small subunit && PF05119:Phage terminase, small subunit
MW_RS12885	-2.766082254	APC family permease && PF13520:Amino acid permease
MW_RS09790	-2.765339339	tRNA-Ala && -
MW_RS10380	-2.765331112	phage major capsid protein && PF05065:Phage capsid family
MW_RS12680	-2.750352786	immunoglobulin-binding protein Sbi && PF02216:B domain|PF11621:C3 binding domain 4 of IgG-bind protein SBI
MW_RS00270	-2.748918548	GNAT family N-acetyltransferase && PF00583:Acetyltransferase (GNAT) family
MW_RS10375	-2.73819273	hypothetical protein && -
MW_RS07400	-2.730385763	DUF1672 domain-containing protein && PF07901:Protein of unknown function (DUF1672)
MW_RS04080	-2.726801687	DUF1474 family protein && PF07342:Protein of unknown function (DUF1474)
MW_RS10360	-2.724281622	HK97 gp10 family phage protein && -
MW_RS13485	-2.721347879	GNAT family N-acetyltransferase && PF13508:Acetyltransferase (GNAT) domain
MW_RS12040	-2.690689653	CHAP domain-containing protein && PF05257:CHAP domain
MW_RS07450	-2.684356045	minor structural protein && -
MW_RS10245	-2.68141862	sphingomyelin phosphodiesterase && -
MW_RS10525	-2.672973211	phage antirepressor KilAC domain-containing protein && PF03374:Phage antirepressor protein KilAC domain|PF08346:AntA/AntB antirepressor
MW_RS07455	-2.665766298	hypothetical protein && -
MW_RS04380	-2.661076494	DUF3055 domain-containing protein && PF11256:Protein of unknown function (DUF3055)
MW_RS12460	-2.655968896	magnesium transporter CorA family protein && PF01544:CorA-like Mg2+ transporter protein
MW_RS07095	-2.633808881	queuosine precursor transporter && PF02592:Putative vitamin uptake transporter
MW_RS08555	-2.630208088	A24 family peptidase && PF06750:Bacterial Peptidase A24 N-terminal domain
MW_RS07425	-2.621819412	phage holin && PF04688:SPP1 phage holin
MW_RS01125	-2.619718232	hypothetical protein && -
MW_RS02090	-2.614726851	FKLRK protein && -
MW_RS10370	-2.609894966	head-tail connector protein && PF05135:Phage gp6-like head-tail connector protein
MW_RS11990	-2.60877715	urease subunit alpha && PF00449:Urease alpha-subunit, N-terminal domain|PF01979:Amidohydrolase family
MW_RS07680	-2.586130577	DUF2829 domain-containing protein && PF11195:Protein of unknown function (DUF2829)
MW_RS10310	-2.579542007	hypothetical protein && -
MW_RS02915	-2.56961947	protein VraC && -
MW_RS13475	-2.561724814	CHAP domain-containing protein && PF05257:CHAP domain
MW_RS07485	-2.559114873	Ig-like domain-containing protein && PF02368:Bacterial Ig-like domain (group 2)
MW_RS12585	-2.548038019	formate/nitrite transporter family protein && PF01226:Formate/nitrite transporter

MW_RS07655	-2.53746614	hypothetical protein && -
MW_RS10350	-2.537055839	Ig-like domain-containing protein && PF02368:Bacterial Ig-like domain (group 2)
MW_RS10400	-2.531900352	phage terminase large subunit && PF03354:Phage Terminase
MW_RS13490	-2.528426038	lytic transglycosylase IsaA && PF01464:Transglycosylase SLT domain
MW_RS10495	-2.522751782	DUF1108 family protein && PF06531:Protein of unknown function (DUF1108)
MW_RS01250	-2.521519711	response regulator transcription factor LytR && PF00072:Response regulator receiver domain|PF04397:LytTr DNA-binding domain
MW_RS00610	-2.520187121	phosphonate ABC transporter ATP-binding protein && PF00005:ABC transporter
MW_RS04640	-2.49966193	membrane protein && -
MW_RS10335	-2.4799189	phage tail tape measure protein && PF01551:Peptidase family M23|PF10145:Phage-related minor tail protein
MW_RS05650	-2.473162832	superantigen-like protein SSL14 && PF02876:Staphylococcal/Streptococcal toxin, beta-grasp domain
MW_RS10510	-2.472968083	hypothetical protein && -
MW_RS09205	-2.472093693	hypothetical protein && -
MW_RS10485	-2.470503896	AAA family ATPase && PF13476:AAA domain
MW_RS04220	-2.467768236	phosphoglycerate mutase family protein && PF00300:Histidine phosphatase superfamily (branch 1)
MW_RS00560	-2.463602441	superoxide dismutase && PF02777:Iron/manganese superoxide dismutases, C-terminal domain|PF00081:Iron/manganese superoxide dismutases, alpha-hairpin domain
MW_RS03645	-2.457876279	hypothetical protein && -
MW_RS10575	-2.448987297	bi-component leukocidin LukGH subunit H && PF07968:Leukocidin/Hemolysin toxin family
MW_RS04550	-2.448472142	argininosuccinate lyase && PF14698:Argininosuccinate lyase C-terminal|PF00206:Lyase
MW_RS04055	-2.448279661	hypothetical protein && -
MW_RS10365	-2.438074944	head-tail adaptor protein && PF05521:Phage head-tail joining protein
MW_RS09355	-2.435680991	serine protease SplF && PF13365:Trypsin-like peptidase domain
MW_RS00605	-2.425678878	phosphonate ABC transporter%2C permease protein PhnE && PF00528:Binding-protein-dependent transport system inner membrane component
MW_RS09105	-2.422137787	TIGR01212 family radical SAM protein && PF04055:Radical SAM superfamily|PF16199:Radical_SAM C-terminal domain
MW_RS02085	-2.417886984	superantigen-like protein SSL11 && PF09199:Staphylococcal superantigen-like OB-fold domain|PF02876:Staphylococcal/Streptococcal toxin, beta-grasp domain
MW_RS10535	-2.417344654	transcriptional regulator && -
MW_RS05575	-2.413028681	formyl peptide receptor-like 1 inhibitory protein && PF16104:Formyl peptide receptor-like 1 inhibitory protein
MW_RS10505	-2.409433975	DUF771 domain-containing protein && PF05595:Domain of unknown function (DUF771)
MW_RS07465	-2.389838164	phage tail family protein && PF05709:Phage tail protein
MW_RS02010	-2.388569037	superantigen-like protein SSL1 && PF09199:Staphylococcal superantigen-like OB-fold domain|PF02876:Staphylococcal/Streptococcal toxin, beta-grasp domain
MW_RS09210	-2.383599476	fluoride efflux transporter CrcB && PF02537:CrcB-like protein, Camphor Resistance (CrcB)
MW_RS05890	-2.37958531	hypothetical protein && -
MW_RS09465	-2.374926737	tRNA-Gly && -
MW_RS11600	-2.353888134	acetolactate decarboxylase && PF03306:Alpha-acetolactate decarboxylase
MW_RS10540	-2.347970604	DUF739 family protein && PF05339:Protein of unknown function (DUF739)
MW_RS09165	-2.336631913	arsenite efflux transporter membrane subunit ArsB && PF02040:Arsenical pump membrane protein
MW_RS12675	-2.33524629	hypothetical protein && -
MW_RS10565	-2.33349637	sphingomyelin phosphodiesterase && PF03372:Endonuclease/Exonuclease/phosphatase family
MW_RS03240	-2.33262399	metal ABC transporter permease && PF00950:ABC 3 transport family
MW_RS01795	-2.320575136	30S ribosomal protein S6 && PF01250:Ribosomal protein S6
MW_RS04125	-2.32032708	staphylococcal enterotoxin type C2 && PF02876:Staphylococcal/Streptococcal toxin, beta-grasp domain|PF01123:Staphylococcal/Streptococcal toxin, OB-fold domain

MW_RS07470	-2.315333097	phage tail tape measure protein && PF10145:Phage-related minor tail protein|PF01551:Peptidase family M23
MW_RS07440	-2.311473902	DUF2977 domain-containing protein && PF11192:Protein of unknown function (DUF2977)
MW_RS10330	-2.308824466	phage tail family protein && PF05709:Phage tail protein
MW_RS03630	-2.299010642	response regulator transcription factor SaeR && PF00072:Response regulator receiver domain|PF00486:Transcriptional regulatory protein, C terminal
MW_RS09365	-2.29765564	serine protease SplB && PF00089:Trypsin
MW_RS00600	-2.29706375	phosphonate ABC transporter%2C permease protein PhnE && PF00528:Binding-protein-dependent transport system inner membrane component
MW_RS03045	-2.296938491	DUF443 family protein && PF04276:Protein of unknown function (DUF443)
MW_RS12690	-2.296640299	bi-component gamma-hemolysin HlgAB subunit A && PF07968:Leukocidin/Hemolysin toxin family
MW_RS12960	-2.290573762	ABC transporter permease && PF12911:N-terminal TM domain of oligopeptide transport permease C|PF00528:Binding-protein-dependent transport system inner membrane component
MW_RS04130	-2.273597427	staphylococcal enterotoxin type L && PF02876:Staphylococcal/Streptococcal toxin, beta-grasp domain
MW_RS10560	-2.26915246	site-specific integrase && PF00589:Phage integrase family|PF14659:Phage integrase, N-terminal SAM-like domain
MW_RS01130	-2.268063447	nitric oxide dioxygenase && PF00970:Oxidoreductase FAD-binding domain|PF00042:Globin
MW_RS02220	-2.26675518	autolysin/adhesin Aaa && PF01476:LysM domain|PF05257:CHAP domain
MW_RS07650	-2.259170926	hypothetical protein && -
MW_RS10220	-2.259127748	hypothetical protein && -
MW_RS07715	-2.254430604	site-specific integrase && PF00589:Phage integrase family
MW_RS12455	-2.252306375	TetR/AcrR family transcriptional regulator && PF00440:Bacterial regulatory proteins, tetR family|PF14278:Transcriptional regulator C-terminal region
MW_RS08040	-2.249684937	50S ribosomal protein L33 && PF00471:Ribosomal protein L33
MW_RS10385	-2.246426994	HK97 family phage prohead protease && PF04586:Caudovirus prohead serine protease
MW_RS10410	-2.238056257	HNH endonuclease && PF01844:HNH endonuclease
MW_RS09405	-2.236533021	flavoprotein && PF02441:Flavoprotein
MW_RS02940	-2.234498185	DUF5327 family protein && -
MW_RS01790	-2.231403226	hypothetical protein && -
MW_RS04070	-2.227036447	helix-turn-helix domain-containing protein && PF01381:Helix-turn-helix
MW_RS10325	-2.225970535	hypothetical protein && -
MW_RS11570	-2.214207261	hypothetical protein && -
MW_RS12860	-2.209977809	ABC transporter permease && PF00528:Binding-protein-dependent transport system inner membrane component
MW_RS06210	-2.205682381	30S ribosomal protein S15 && PF00312:Ribosomal protein S15
MW_RS06380	-2.204869633	MerR family transcriptional regulator && PF13411:MerR HTH family regulatory protein
MW_RS09895	-2.187370698	radical SAM/CxCxxxxC motif protein YfkAB && PF04055:Radical SAM superfamily|PF08756:YfkB-like domain
MW_RS03425	-2.177410729	HTH-type transcriptional regulator SarX && -
MW_RS03080	-2.171671083	DUF443 domain-containing protein && PF04276:Protein of unknown function (DUF443)
MW_RS11265	-2.169616204	Zn(II)-responsive metalloregulatory transcriptional repressor CzrA && PF01022:Bacterial regulatory protein, arsR family
MW_RS02405	-2.159805145	Veg family protein && PF06257:Biofilm formation stimulator VEG
MW_RS13280	-2.147776649	hypothetical protein && -
MW_RS00215	-2.142017781	hypothetical protein && -
MW_RS00300	-2.13366614	persulfide dioxygenase-sulfurtransferase CstB && PF00753:Metallo-beta-lactamase superfamily|PF00581:Rhodanese-like domain
MW_RS07430	-2.130093693	DUF2951 domain-containing protein && PF11166:Protein of unknown function (DUF2951)
MW_RS08425	-2.128085095	tRNA threonylcarbamoyladenosine dehydratase && PF00899:ThiF family
MW_RS13965	-2.119215455	accessory Sec system protein Asp1 && PF16993:Accessory Sec system protein Asp1

MW_RS04475	-2.117792303	FAD/NAD(P)-binding protein && PF13434:L-lysine 6-monooxygenase (NADPH-requiring)
MW_RS09660	-2.117405532	tRNA-Gly && -
MW_RS09910	-2.113403047	DUF1128 family protein && PF06569:Protein of unknown function (DUF1128)
MW_RS08665	-2.113128406	translation initiation factor IF-3 && PF00707:Translation initiation factor IF-3, C-terminal domain|PF05198:Translation initiation factor IF-3, N-terminal domain
MW_RS08655	-2.112542568	50S ribosomal protein L20 && PF00453:Ribosomal protein L20
MW_RS09110	-2.107162984	class I SAM-dependent methyltransferase && PF06962:Putative rRNA methylase
MW_RS07785	-2.104895487	hypothetical protein && -
MW_RS12485	-2.093850117	DUF4889 domain-containing protein && PF16230:Domain of unknown function (DUF4889)
MW_RS01060	-2.093625534	glycerophosphoryl diester phosphodiesterase membrane domain-containing protein && PF10110:Membrane domain of glycerophosphoryl diester phosphodiesterase|PF03009:Glycerophosphoryl diester phosphodiesterase family
MW_RS09415	-2.090386229	lantibiotic dehydratase && PF14028:Lantibiotic biosynthesis dehydratase C-term|PF04738:Lantibiotic dehydratase, C terminus
MW_RS04940	-2.087886509	glycosyltransferase && PF00534:Glycosyl transferases group 1
MW_RS11270	-2.078751082	CDF family zinc efflux transporter CzrB && PF01545:Cation efflux family
MW_RS05430	-2.073999242	heme uptake protein IsdB && PF05031:Iron Transport-associated domain|PF04650:YSIRK type signal peptide
MW_RS05460	-2.071181104	class B sortase && PF04203:Sortase family
MW_RS10390	-2.068028555	phage portal protein && PF04860:Phage portal protein
MW_RS11435	-2.064250552	YjiH family protein && PF07670:Nucleoside recognition
MW_RS05445	-2.052748762	iron-regulated surface determinant protein IsdD && -
MW_RS13765	-2.052505542	CitMHS family transporter && PF03600:Citrate transporter
MW_RS05230	-2.035194659	hypothetical protein && -
MW_RS13595	-2.031906896	quinone-dependent dihydroorotate dehydrogenase && PF01180:Dihydroorotate dehydrogenase
MW_RS00450	-2.030675387	staphyloferrin B ABC transporter permease subunit SirC && PF01032:FecCD transport family
MW_RS00370	-2.024614481	phosphatidylinositol-specific phospholipase C && PF00388:Phosphatidylinositol-specific phospholipase C, X domain
MW_RS00165	-2.024424631	PBP2a family beta-lactam-resistant peptidoglycan transpeptidase MecA && PF05223:NTF2-like N-terminal transpeptidase domain|PF03717:Penicillin-binding Protein dimerisation domain|PF00905:Penicillin binding protein transpeptidase domain
MW_RS09420	-2.021271753	gallidermin/nisin family lantibiotic && PF02052:Gallidermin
MW_RS00265	-2.018596875	staphylococcal enterotoxin type H && PF01123:Staphylococcal/Streptococcal toxin, OB-fold domain|PF02876:Staphylococcal/Streptococcal toxin, beta-grasp domain
MW_RS13955	-2.01623208	accessory Sec system protein Asp3 && PF15432:Accessory Sec secretory system ASP3
MW_RS06335	-2.013976954	aquaporin family protein && PF00230:Major intrinsic protein
MW_RS10250	-2.004041505	hypothetical protein && -
MW_RS10480	-2.003633403	recombinase RecT && PF03837:RecT family
MW_RS10570	-2.00001074	bi-component leukocidin LukGH subunit G && PF07968:Leukocidin/Hemolysin toxin family

Supplemental Table 9. MIC of CIT-8 against *PdxS* transposon mutant from the NTML library in presence of 100 µg/ml vitamin B6.

**Strain description and condition**	**MIC (µg/ml)**
PdxS transposon mutant	4
PdxS transposon mutant + 100 µg/ml vitamin B6	4
JE2	4
JE2 + 100 µg/ml vitamin B6	4

Supplemental Table 10. List of bacterial strains used in this study.

Sl no.	***S. aureus* strains**	**Description**
1	VRS1	VRSA
2	JE2	MRSA
3	AR0215	VISA
4	AR0216	VISA
5	AR0217	VISA
6	AR0219	VISA
7	AR0225	VISA
8	BF1	Clinical isolate
9	BF2	Clinical isolate
10	BF3	Clinical isolate
11	BF4	Clinical isolate
12	BF5	Clinical isolate
13	BF6	Clinical isolate
14	BF7	Clinical isolate
15	BF8	Clinical isolate
16	BF9	Clinical isolate
17	BF10	Clinical isolate
18	BF11	Clinical isolate

Supplemental Figure 1. Alpha Fold structure of CIT-1. (A) α-helical representation of CIT-1 peptide showing the hydrophobic gap (black arrow). Selective amino acids represented are aspartic acid (at position 4), valine (at position 9), alanine (at position 10) and serine (at position 11). (B) A representation of CIT-1 with polar/ charged amino acids as ball and sticks and hydrophobic amino acids as hydrophobic surface. Structures were generated from https://colab.research.google.com/github/sokrypton/ColabFold/blob/main/AlphaFold2.ipynb

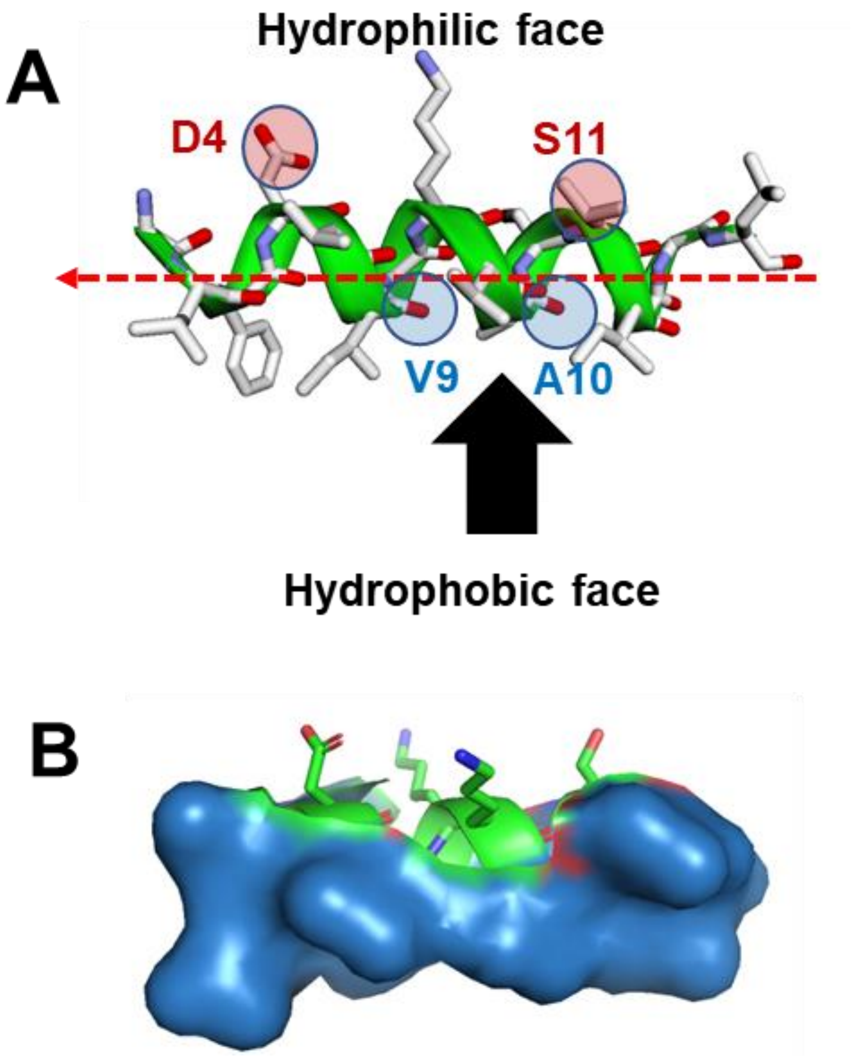

Supplemental Figure 2. CIT-1 to CIT-8 AlphaFold peptide structures and sequences. Orange arrows represent hydrophobic gap. Structures were generated from https://colab.research.google.com/github/sokrypton/ColabFold/blob/main/AlphaFold2.ipynb

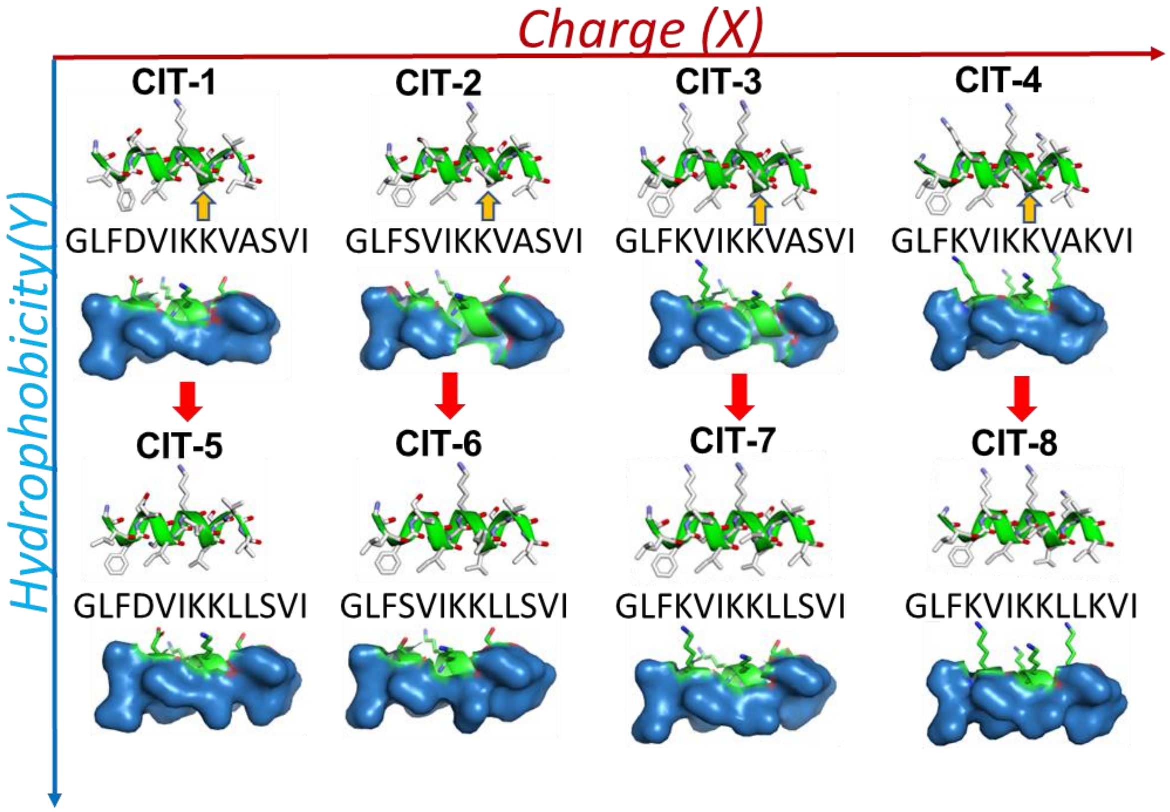

Supplemental Figure 3. Helical wheel plots with hydropathy distribution for peptides CIT-1 to CIT-8. Helical wheel plots were generated using (https://heliquest.ipmc.cnrs.fr/). The hydropathy values were calculated by using the Kyte-Doolittle scale (1).

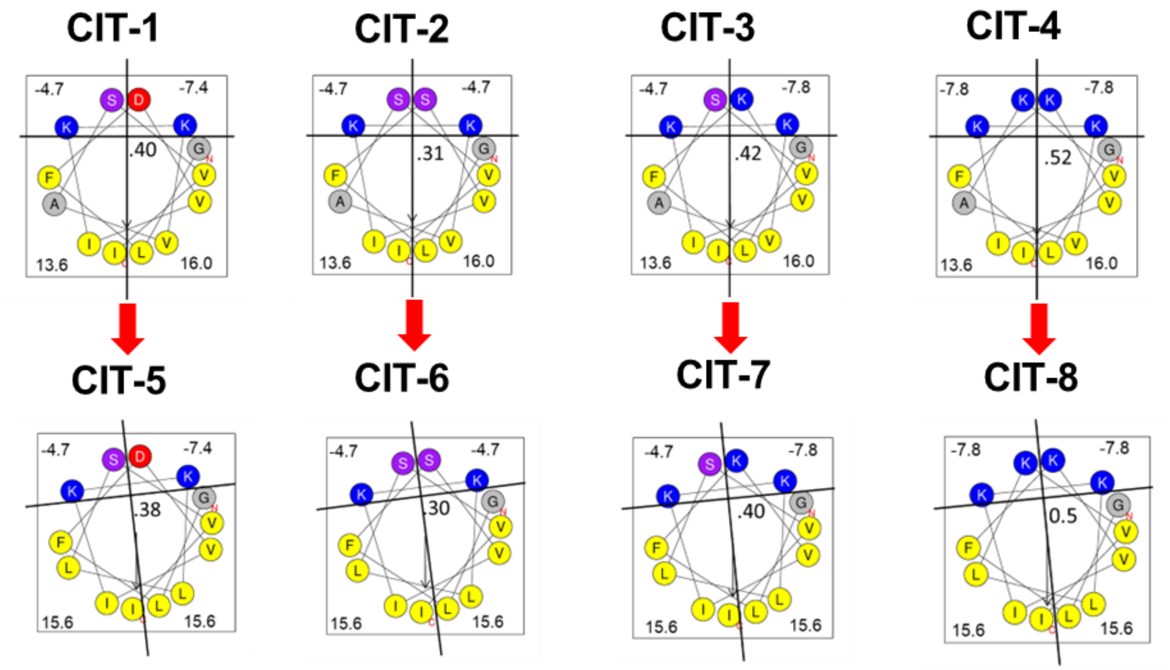

Supplemental Figure 4. Mammalian toxicity assessment of CIT peptides (A) Hemolysis potential of citropin 1.1-derived peptides on human red blood cells (B) cellular toxicity of CIT-8 to HepG2 cell lines compared with gentamicin control (Genta).

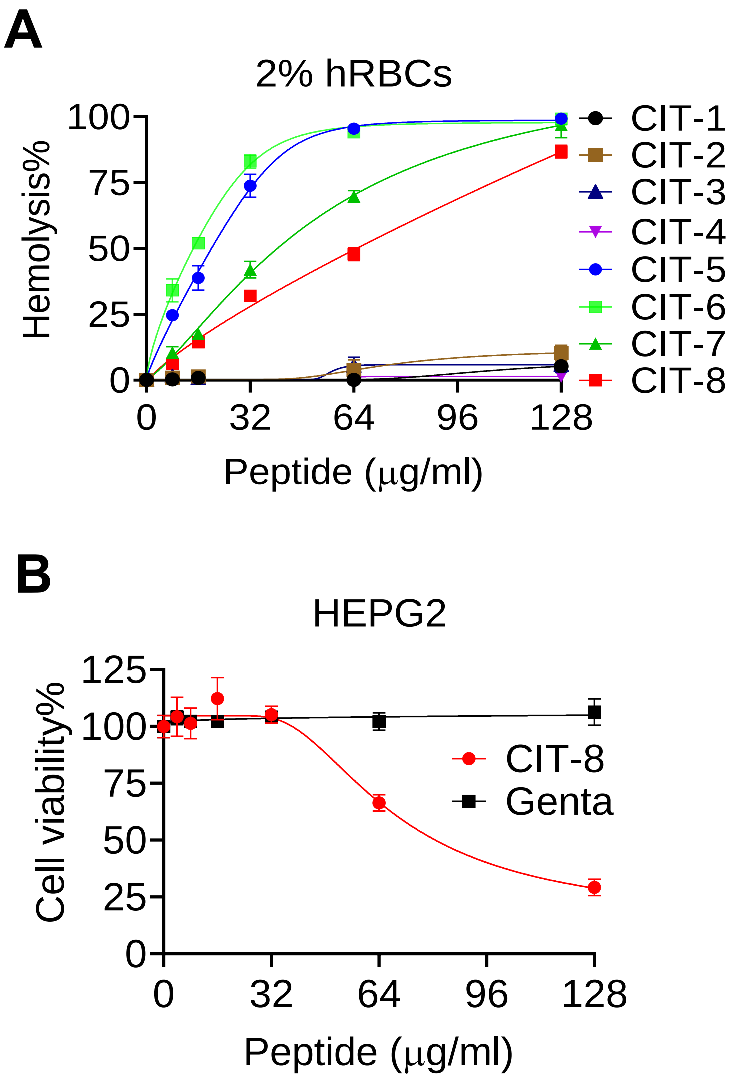

Supplemental Figure 5. Anti-biofilm and anti-persister activity of CIT-8.

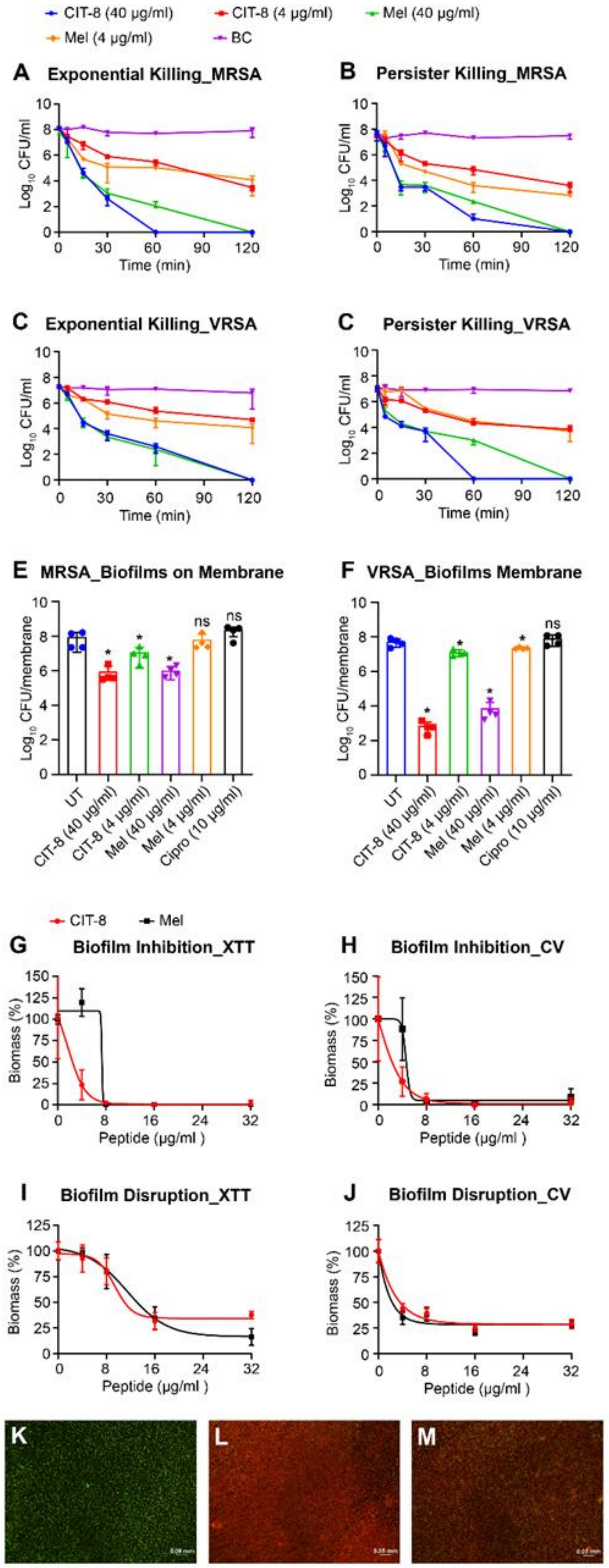

(A-B) Killing kinetics of CIT-8 and melittin (Mel) against *S. aureus* MW2 in (A) exponential phase, and (B) gentamicin-induced persister cells at concentrations of 4 and 40 µg/ml, compared to untreated bacterial controls (BC), CFU counts were monitored over 120 minutes. (C-D) Killing kinetics of CIT-8 and Mel against *S. aureus* VRS1 in (C) exponential phase, and (D) gentamicin-induced persister cells at concentrations of 4 and 40 µg/ml, compared to untreated bacterial controls (BC), CFU counts were monitored over 120 minutes. (E-F) Disruption of 24 hour established biofilms of (E) MRSA (S. *aureus* MW2), and (F) VRSA (S. *aureus* VRS1) by CIT-8 and Mel, measured as log reductions in bacterial loads on solid membranes treated with 4 and 40 µg/ml of each peptide (*$p < 0.05$, student’s t-test; ns: non-significant). (G-H) Inhibition of biofilm formation by CIT-8 and Mel at concentrations ranging from 4–32 µg/ml after 24 hours of treatment, assessed using (G) live-cell viability (XTT assay), and (H) biomass quantification (crystal violet staining). (I-J) Disruption of 24 hour established biofilms by CIT-8 and Mel at 4–32 µg/ml, evaluated by (I) reductions in live-cell viability (XTT assay) and (J) biomass loss (crystal violet staining). (K-M) Fluorescence microscopy images (10×) of 24-hour-established *S. aureus* MW2 biofilms: (K) untreated control, (L) biofilms treated with 32 µg/ml of CIT-8, and (M) biofilms treated with 32 µg/ml of Mel. Live/dead staining highlights the proportion of dead cells within the biofilms.

Supplemental Figure 6. Secondary structure conformation of the peptide CIT-8 in the presence of SDS micelles using circular dichroism.

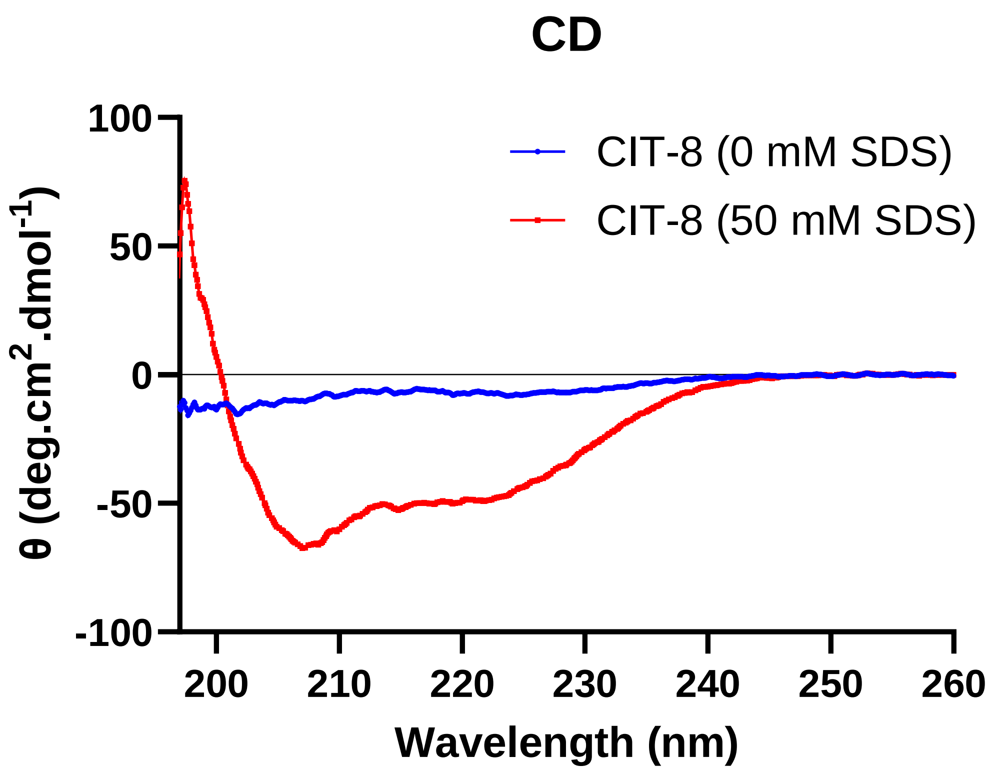

Supplemental Figure 7. Change in lipid to surface area ratio upon CIT-8 binding to DOPC: DOPG (7:3) model membrane.

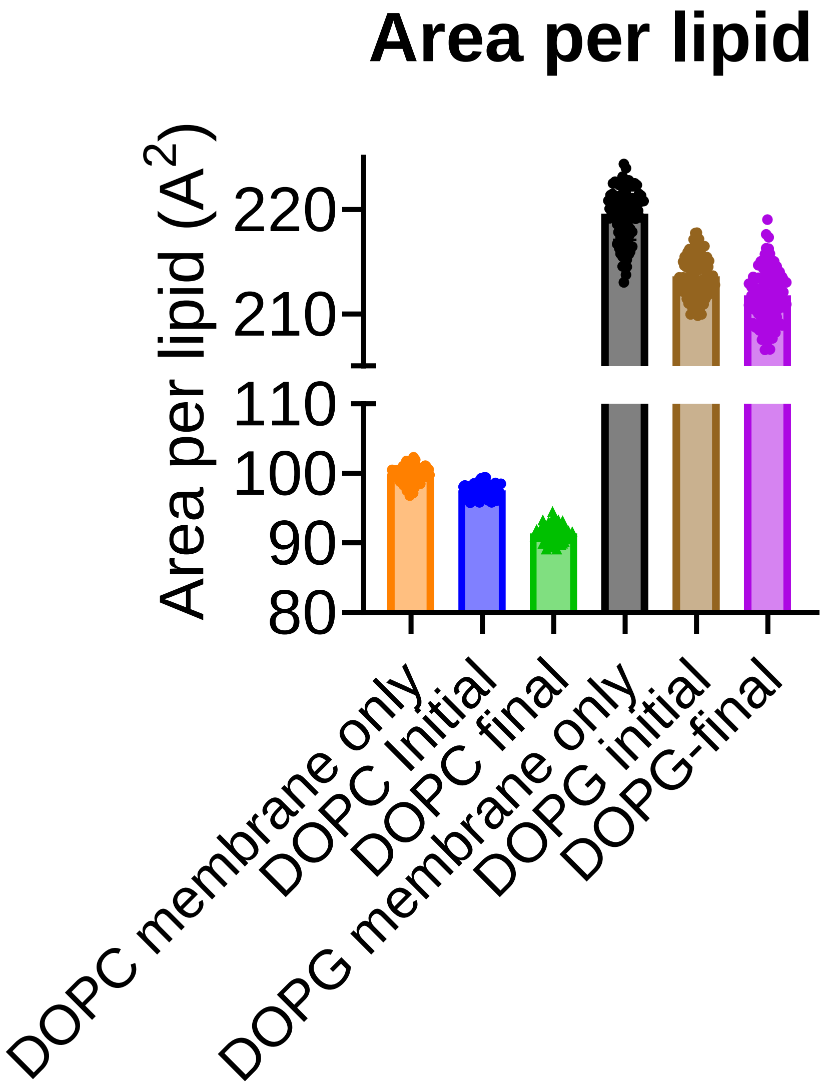

Supplemental Figure 8. Florescence-based membrane permeation of *S. aureus* MW2 evaluated using (A) PI and (B) SYTOX green by Hylaseptin P1, Mastoparan L, and r-Camel peptide templates and their ML-designed corresponding peptides at 32 µg/ml compared to untreated bacteria (UT). Vancomycin (Vanc) and melittin (Mel) at 32 µg/ml were used as controls. Statistical significance (**** denotes p < 0.0001, ns: non-significant) were determined using one way ANOVA.

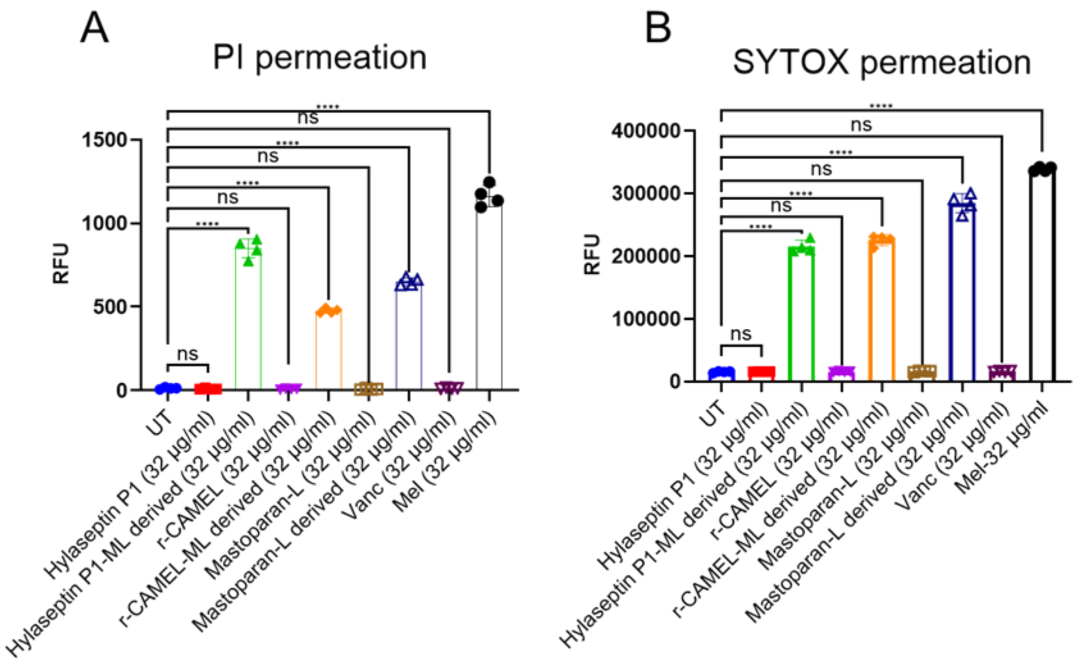

Supplemental Figure 9. Pathways downregulated in *S. aureus* MW2 upon CIT-8 interaction at 0.5 × MIC (Cutoff 2-fold) identified by RNA-seq.

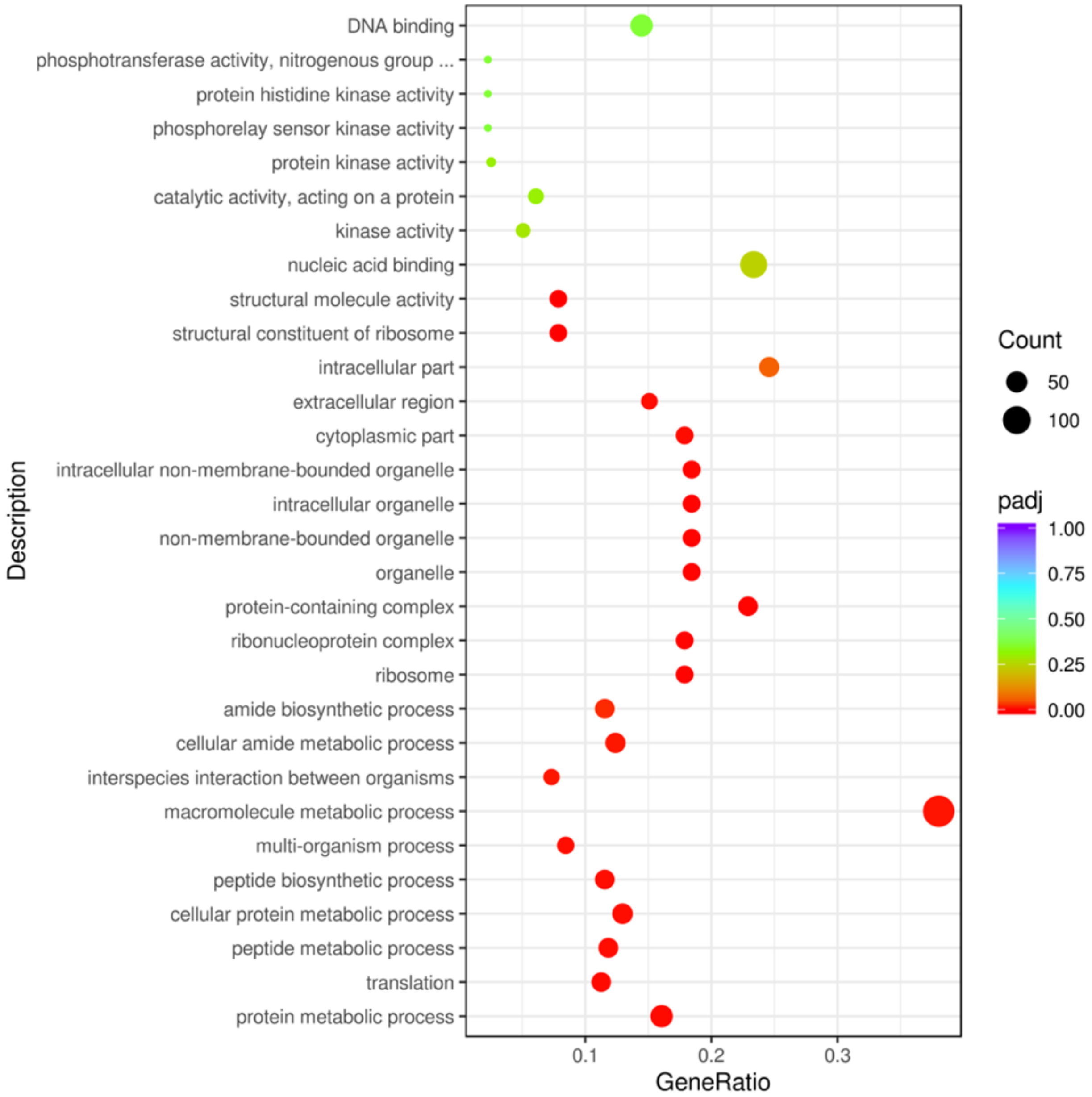

Supplemental Figure 10. Partial least squares-discriminant analysis (PLSDA) plots of metabolites between *S. aureus* MW2 control and CIT-8 treated conditions with three technical replicates.

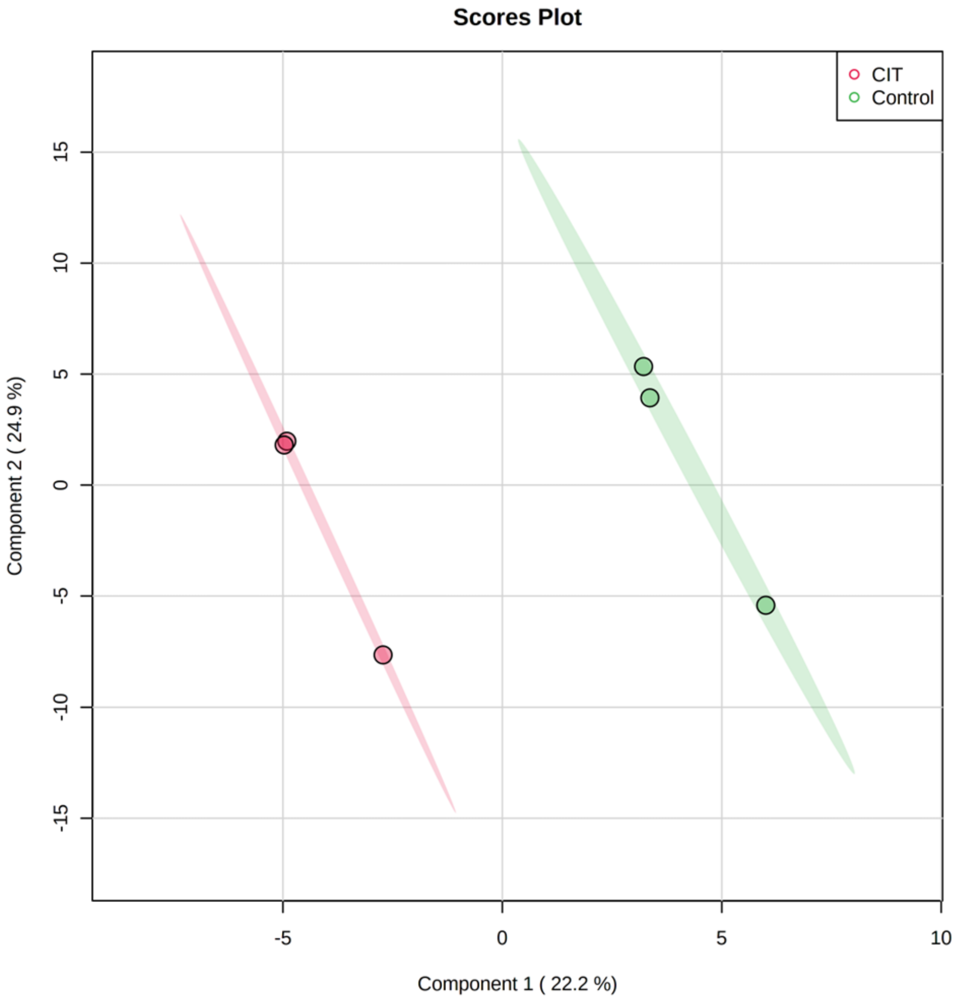

Both graphs show that the *S. aureus* MW2 control (green) and CIT-8 treated (red) bacterial samples are separated, indicating a very distinct metabolite composition. Component 1 indicates the degree of variation between the groups based on their total metabolite content, and component 2 indicates the differences within the groups.

Supplemental Figure 11. Heatmap representation of significantly altered metabolites in *S. aureus* MW2 bacteria treated with CIT-8 (n=3, replicates).

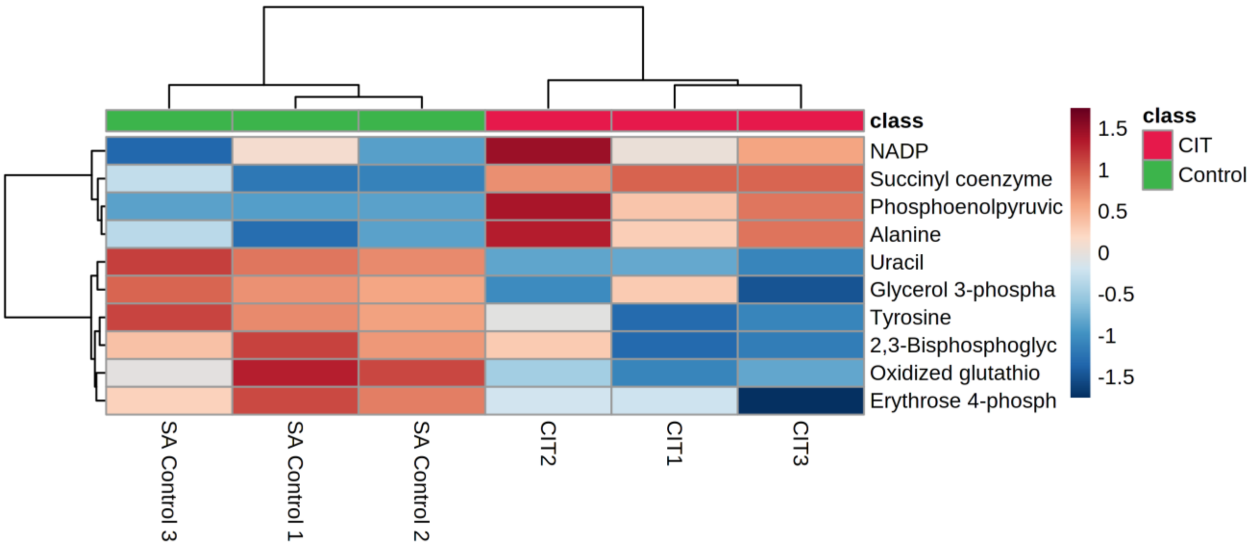

Supplemental Figure 12. Experimental repeat of *in vivo* efficacy of CIT-8 in a skin-abraded prophylactic murine model infected with *S. aureus* MW2. Quantified bacterial load from skin specimens treated after 10 min of bacterial infection with CIT-8 (2% w/w), CIT-8 (1% w/w), and mupirocin (2% w/w) ointments compared with vehicle control (**denotes $p < 0.01$, calculated by one-way ANOVA).

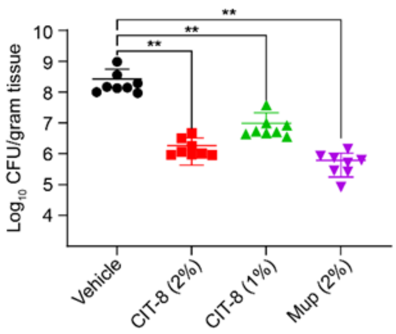

Supplemental Figure 13. Histopathology of vehicle and CIT-8 treated skin in the skin-abraded prophylactic murine model infected with *S. aureus* MW2.

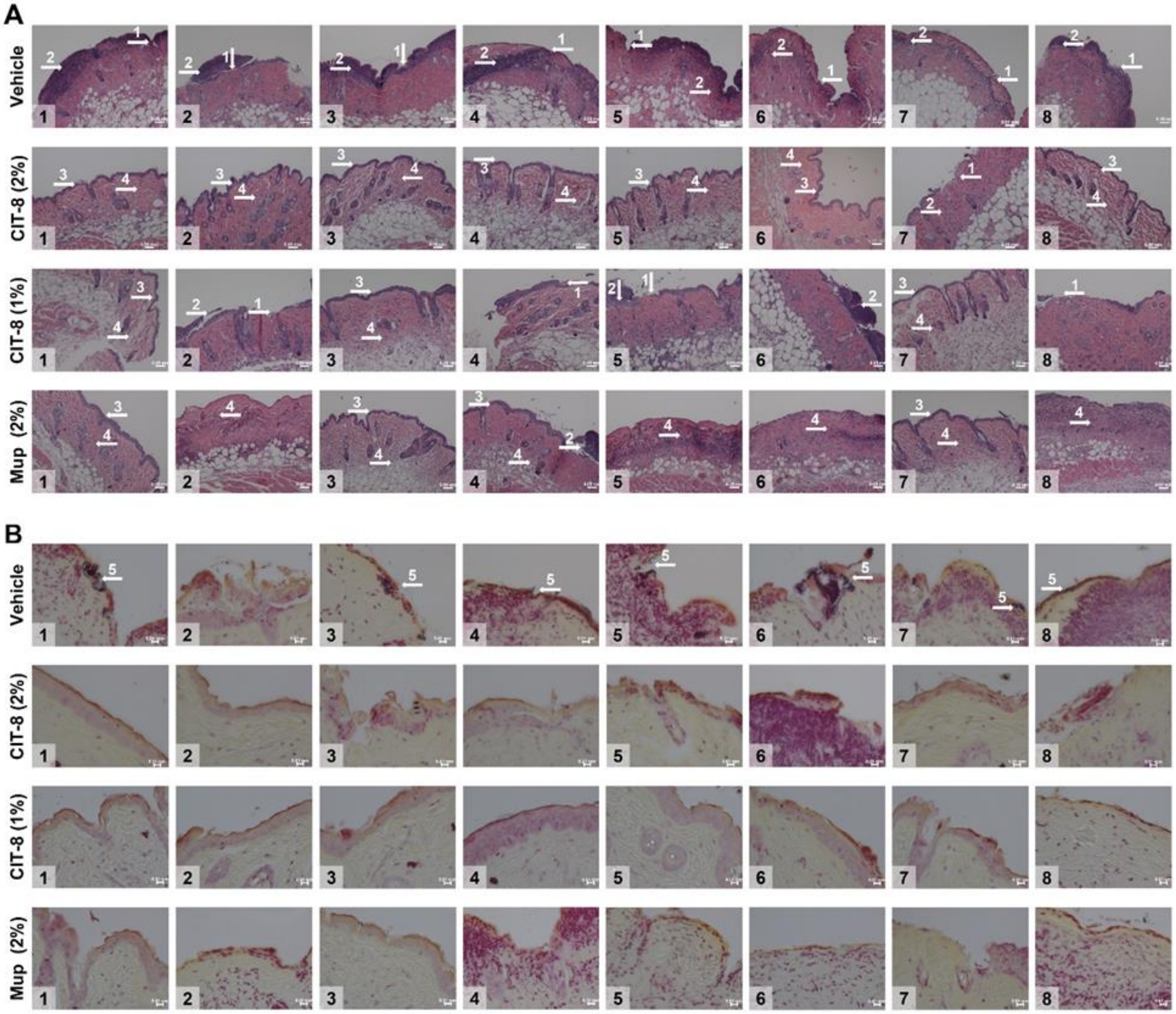

Panel A: Representative 10× images of H&E-stained murine skin sections from all n=32 animals (n=8 per group). Scale bars: 0.05 mm. Panel B: Representative 40× images of Gram-stained murine skin sections. Scale bars: 0.01 mm. White arrows (1-5) indicate key histopathological features; 1: Disorganized epidermal layer with epidermal disruption; 2: Dense infiltration of polymorphonuclear (PMN) and mononuclear (MN) cells in both the epidermis and dermis, indicative of heightened inflammation; 3: More intact epidermis; 4: Reduced numbers of PMN and MN cells; 5: Bacterial patches.

Supplemental Figure 14. Peptide characterization data (HPLC and MS)

Sample Name :CIT1
Sample ID :U6362HA280-1
Time Processed :16:20:53
Month-Day-Year Processed :02/10/2022

Pump A : 0.065% trifluoroacetic in 100% water (v/v)
Pump B : 0.05% trifluoroacetic in 100% acetonitrile (v/v)
Total Flow:1 ml/min
Wavelength:220 nm
<<LC Time Program>>

Time	Module	Command	Value
0.01	Pumps	B.Conc	5
25.00	Pumps	B.Conc	65
25.01	Pumps	B.Conc	95
27.00	Pumps	B.Conc	95
27.01	Pumps	B.Conc	5
35.00	Pumps	B.Conc	5
35.01	Controller	Stop	

<<Column Performance>>
<Detector A>
Column :Inertsil ODS-SP 4.6 x 250 mm
Equipment: ZJ19010324

<Chromatogram>

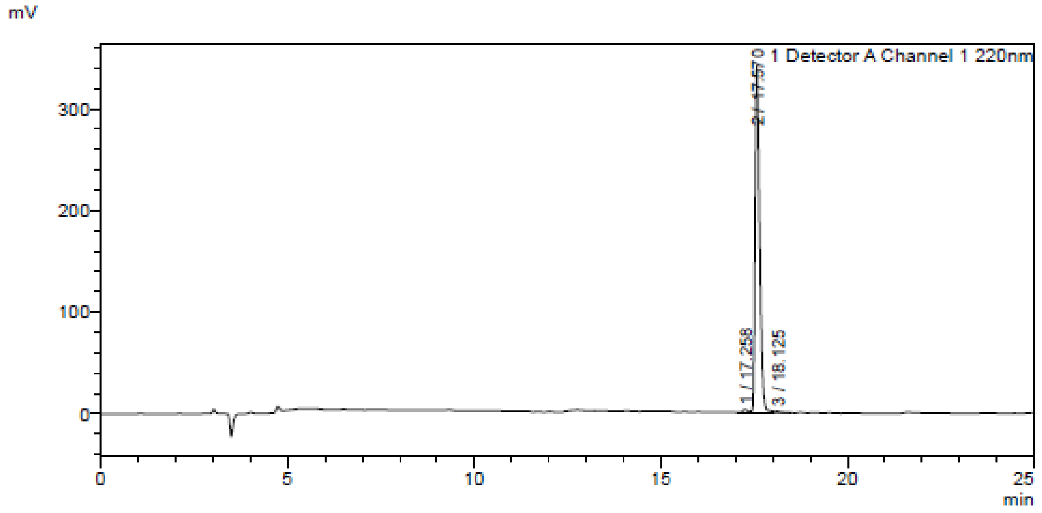

<Peak Table>

Detector A Channel 1 220nm

Peak#	Ret. Time	Area	Height	Area%
1	17.258	24989	2559	0.806
2	17.570	3063620	338936	98.808
3	18.125	11957	1090	0.386
Total		3100566	342584	100.000

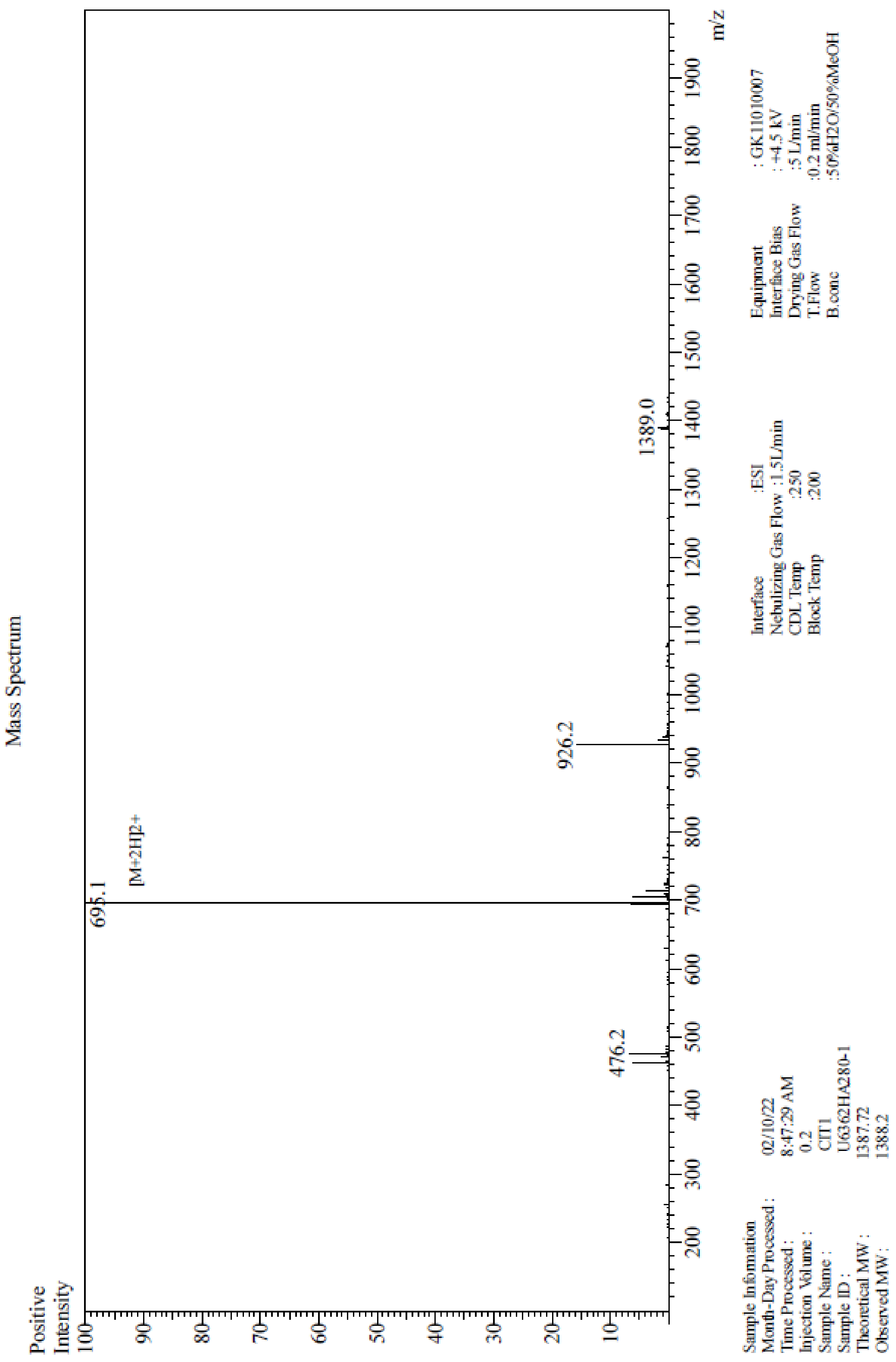

Mass Spectrum
Positive Intensity
100
90
80
70
60
50
40
30
20
10
200
300
400
500
600
700
800
900
1000
1100
1200
1300
1400
1500
1600
1700
1800
1900
m/z
476.2
695.1
[M+2H]2+
926.2
1389.0
Sample Information
Month-Day Processed : 02/10/22
Time Processed : 8:47:29 AM
Injection Volume : 0.2
Sample Name : CIT1
Sample ID : U6362HA280-1
Theoretical MW : 1387.72
Observed MW : 1388.2
Interface :ESI
Nebulizing Gas Flow :1.5L/min
CDL Temp :250
Block Temp :200
Equipment : GK11010007
Interface Bias : +4.5 kV
Drying Gas Flow :5 L/min
T.Flow :0.2 ml/min
B.conc :50%H2O/50%MeOH

Sample Name :CIT2
Sample ID :U6362HA280-3
Time Processed :1:38:39 PM
Month-Day-Year Processed :02/14/2022

Pump A : 0.065% trifluoroacetic in 100% water (v/v)
Pump B : 0.05% trifluoroacetic in 100% acetonitrile (v/v)
Total Flow:1 ml/min
Wavelength:220 nm

Time	Module	Command	Value
0.01	Pumps	Pump A B.Conc	5
25.00	Pumps	Pump A B.Conc	65
25.01	Pumps	Pump A B.Conc	95
27.00	Pumps	Pump A B.Conc	95
27.01	Pumps	Pump A B.Conc	5
35.00	Pumps	Pump A B.Conc	5
35.01	Controller	Stop	

<<Column Performance>>
<Detector A>
Column : Inertsil ODS-3 4.6 x 250 mm

Chromatogram

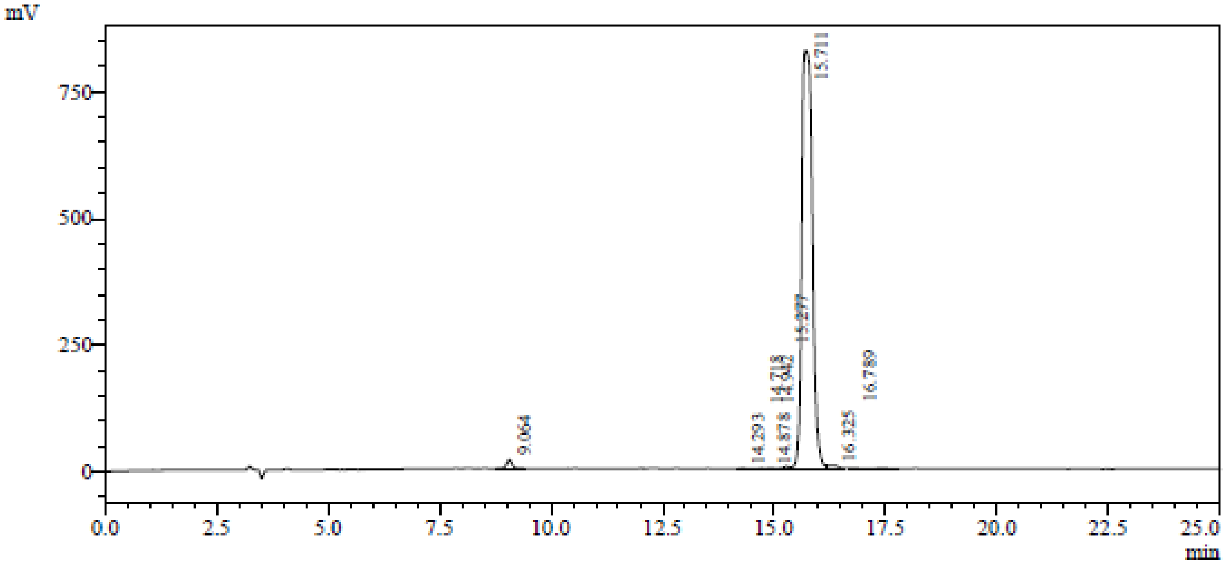

1 Detector A Channel 1 / 220nm

Peak Table

Detector A Channel 1 220nm

Peak#	Ret. Time	Area	Height	Area %
1	9.064	155982	17575	1.102
2	14.293	9702	928	0.069
3	14.718	5477	620	0.039
4	14.878	4675	682	0.033
5	14.942	4650	629	0.033
6	15.277	52955	5433	0.374
7	15.711	13797614	826572	97.463
8	16.325	114667	7143	0.810
9	16.789	11112	937	0.078
Total		14156835	860518	100.000

250

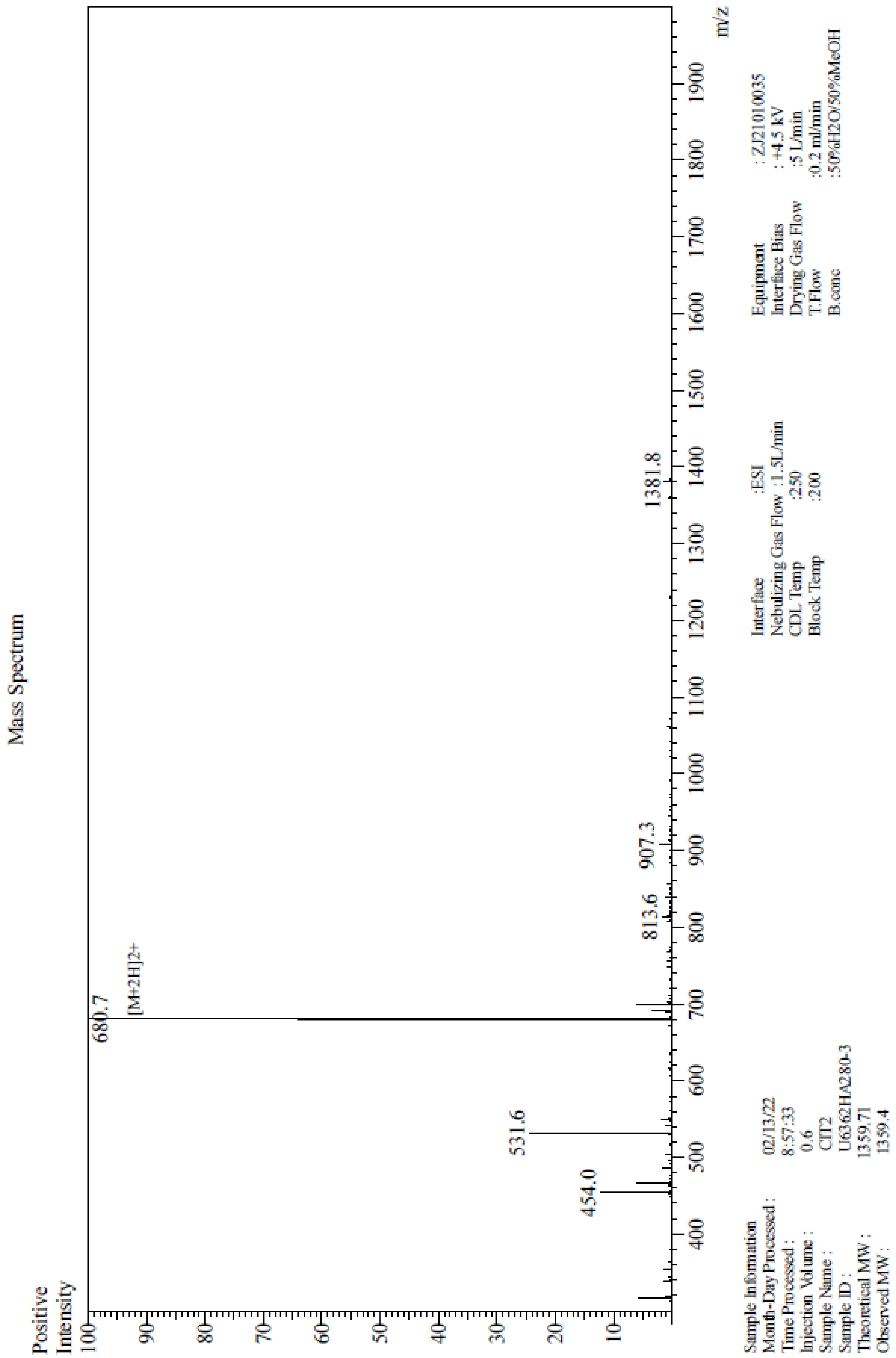
Mass Spectrum
Positive
Intensity
m/z
454.0
531.6
680.7
[M+2H]2+
813.6
907.3
1381.8
Sample Information
Month-Day Processed : 02/13/22
Time Processed : 8:57:33
Injection Volume : 0.6
Sample Name : CIT2
Sample ID : U6362HA280-3
Theoretical MW : 1359.71
Observed MW : 1359.4
Interface :ESI
Nebulizing Gas Flow :1.5L/min
CDL Temp :250
Block Temp :200
Equipment : ZJ2101035
Interface Bias : +4.5 kV
Drying Gas Flow :5 L/min
T.Flow :0.2 ml/min
B.conc :50%H2O/50%MeOH

Sample Name :CIT3
Sample ID :U6362HA280-5
Time Processed :3:57:52
Month-Day-Year Processed :02/15/2022

Pump A : 0.065% trifluoroacetic in 100% water (v/v)
Pump B : 0.05% trifluoroacetic in 100% acetonitrile (v/v)
Total Flow:1 ml/min
Wavelength:220 nm
<<LC Time Program>>

Time	Module	Command	Value
0.01	Pumps	B.Conc	5
25.00	Pumps	B.Conc	65
25.01	Pumps	B.Conc	95
27.00	Pumps	B.Conc	95
27.01	Pumps	B.Conc	5
35.00	Pumps	B.Conc	5
35.01	Controller	Stop	

<<Column Performance>>
<Detector A>
Column :Inertsil ODS-SP 4.6 x 250 mm
Equipment: ZJ21010376

<Chromatogram>

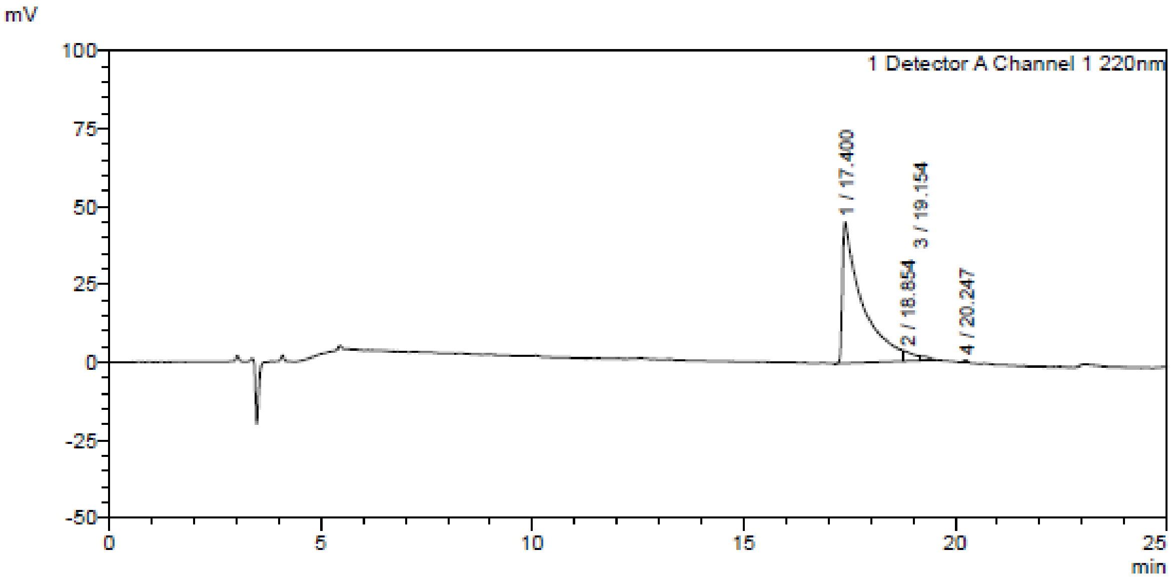

<Peak Table>

Detector A Channel 1 220nm

Peak#	Ret. Time	Area	Height	Area%
1	17.400	1452076	45293	95.295
2	18.854	47854	2981	3.140
3	19.154	18702	1343	1.227
4	20.247	5142	820	0.337
Total		1523774	50437	100.000

Mass Spectrum

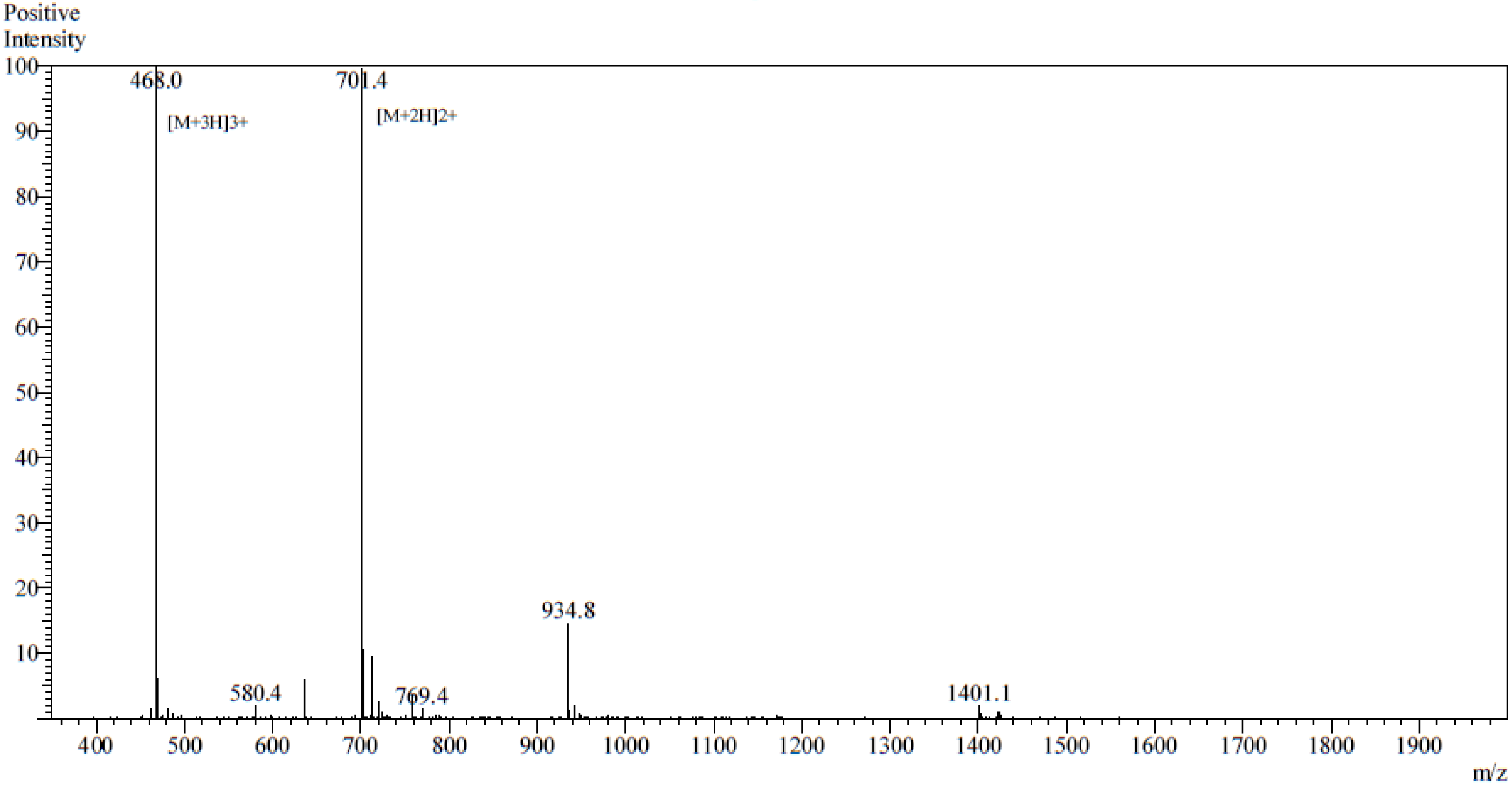

Sample Information	
Month-Day Processed :	02/15/22
Time Processed :	9:05:33 AM
Injection Volume :	0.2
Sample Name :	CIT3
Sample ID :	U6362HA280-5
Theoretical MW :	1400.80
Observed MW :	1400.8

Interface	:ESI
Nebulizing Gas Flow	:1.5L/min
CDL Temp	:250
Block Temp	:200

Equipment	: GK11010007
Interface Bias	: +4.5 kV
Drying Gas Flow	:5 L/min
T.Flow	:0.2 ml/min
B.conc	:50%H2O/50%MeOH

Sample Name :CIT4
Sample ID :U6362HA280-7
Time Processed :22:52:28
Month-Day-Year Processed :02/06/2022

Pump A : 0.065% trifluoroacetic in 100% water (v/v)
Pump B : 0.05% trifluoroacetic in 100% acetonitrile (v/v)
Total Flow:1 ml/min
Wavelength:220 nm
<<LC Time Program>>

Time	Module	Command	Value
0.01	Pumps	B.Conc	5
25.00	Pumps	B.Conc	65
25.01	Pumps	B.Conc	95
27.00	Pumps	B.Conc	95
27.01	Pumps	B.Conc	5
35.00	Pumps	B.Conc	5
35.01	Controller	Stop	

<<Column Performance>>
<Detector A>
Column :Inertsil ODS-3 4.6 x 250 mm
Equipment: SS-CM-0309

<Chromatogram>

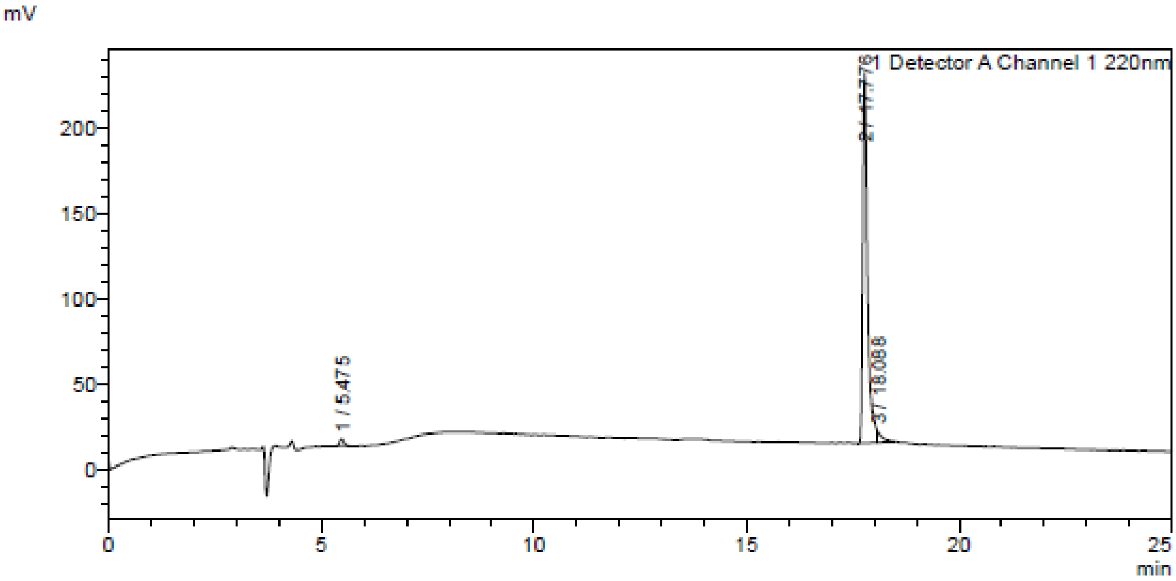

<Peak Table>

Detector A Channel 1 220nm

Peak#	Ret. Time	Area	Height	Area%
1	5.475	26315	4288	1.477
2	17.776	1706666	216055	95.786
3	18.088	48772	6301	2.737
Total		1781753	226644	100.000

258

Mass Spectrum

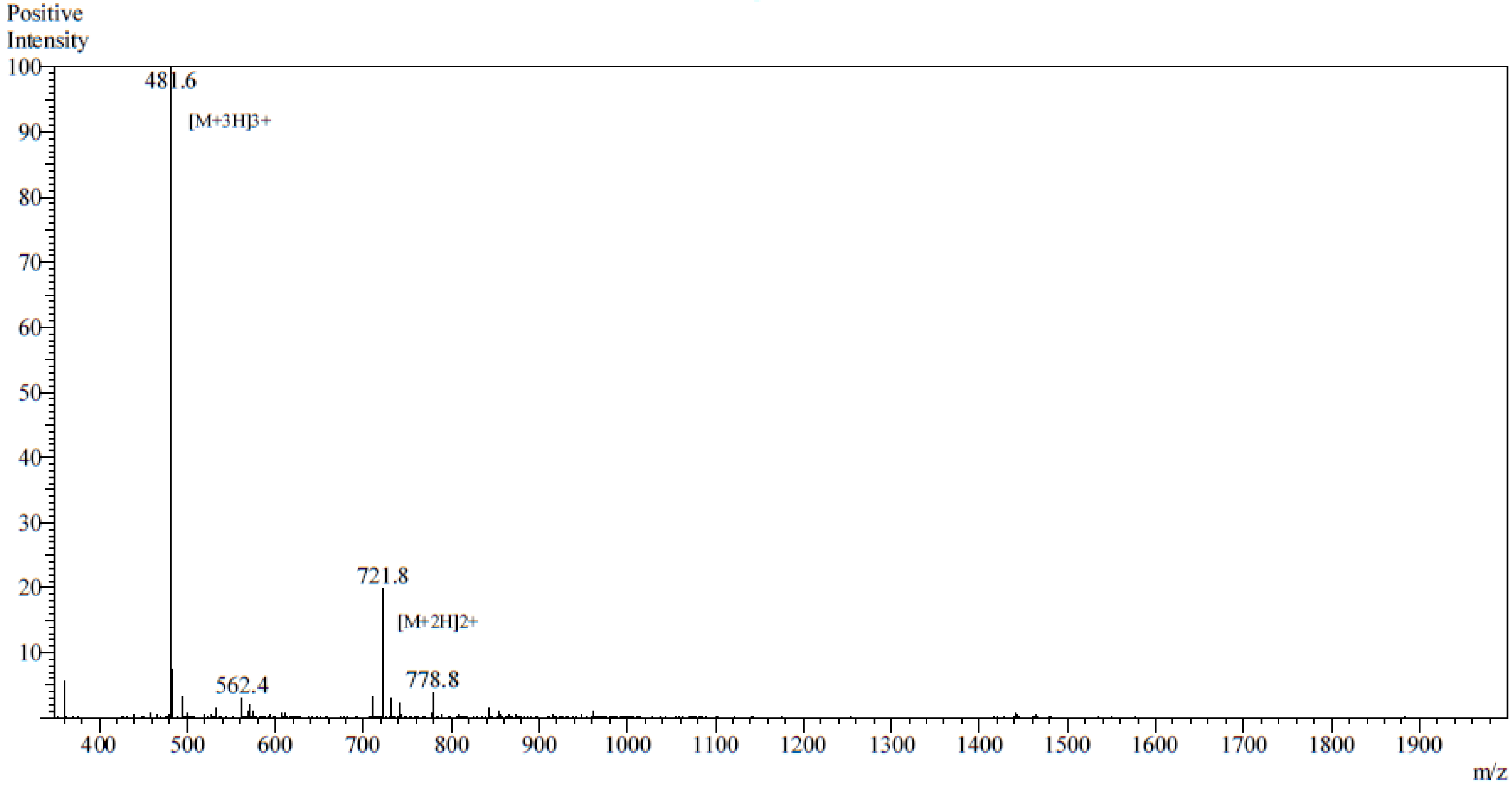

Sample Information	
Month-Day Processed :	02/06/22
Time Processed :	8:42:01 PM
Injection Volume :	0.4
Sample Name :	CIT4
Sample ID :	U6362HA280-7
Theoretical MW :	1441.90
Observed MW :	1441.8

Interface	:ESI
Nebulizing Gas Flow	:1.5L/min
CDL Temp	:250
Block Temp	:200

Equipment	: ZJ21010035
Interface Bias	: +4.5 kV
Drying Gas Flow	:5 L/min
T.Flow	:0.2 ml/min
B.conc	:50%H2O/50%MeOH

Sample Name : CIT5
Sample ID : U6362HA280-9
Time Processed : 15:14:06
Month-Day-Year Processed : 02/12/2022

Pump A : 0.065% trifluoroacetic in 100% water (v/v)
Pump B : 0.05% trifluoroacetic in 100% acetonitrile (v/v)
Total Flow:1 ml/min
Wavelength:220 nm

Time	Module	Command	Value
0.01	Pumps	Pump A B.Conc	5
25.00	Pumps	Pump A B.Conc	65
25.01	Pumps	Pump A B.Conc	95
27.00	Pumps	Pump A B.Conc	95
27.01	Pumps	Pump A B.Conc	5
35.00	Pumps	Pump A B.Conc	5
35.01	Controller	Stop	

<<Column Performance>>
<Detector A>
Column : Inertsil ODS-3 4.6 x 250 mm
Equipment: GK11010017

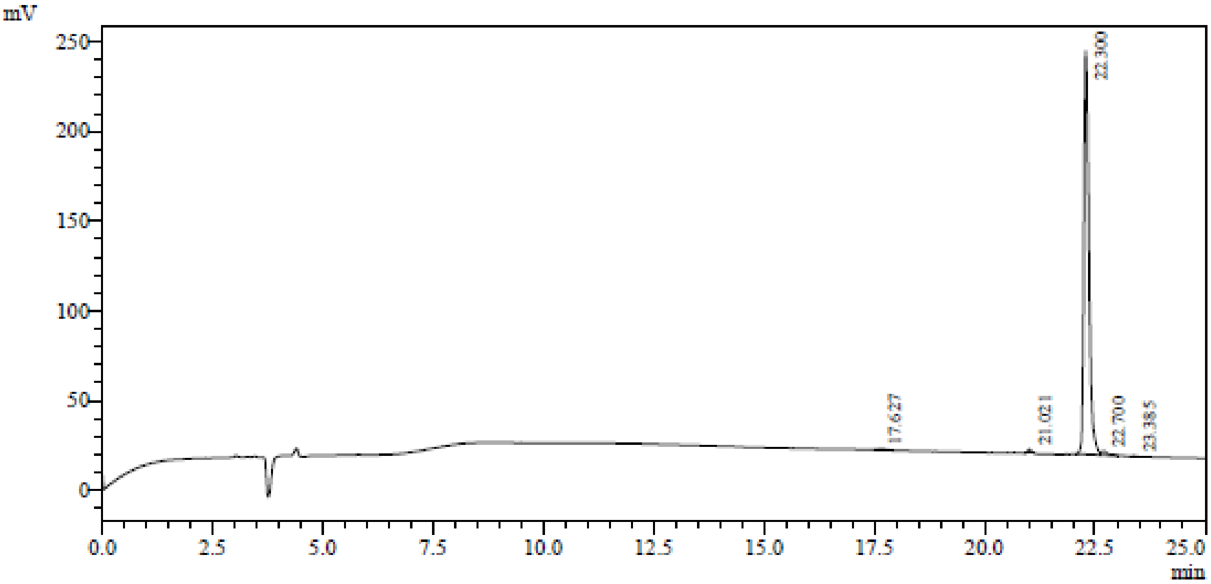

1 Detector A Channel 1 / 220nm

Peak Table

Detector A Channel 1 220nm

Peak#	Ret. Time	Area	Height	Area %
1	17.627	6166	526	0.341
2	21.021	11960	2162	0.662
3	22.300	1762271	224748	97.568
4	22.700	24242	2135	1.342
5	23.385	1561	317	0.086
Total		1806200	229888	100.000

Mass Spectrum

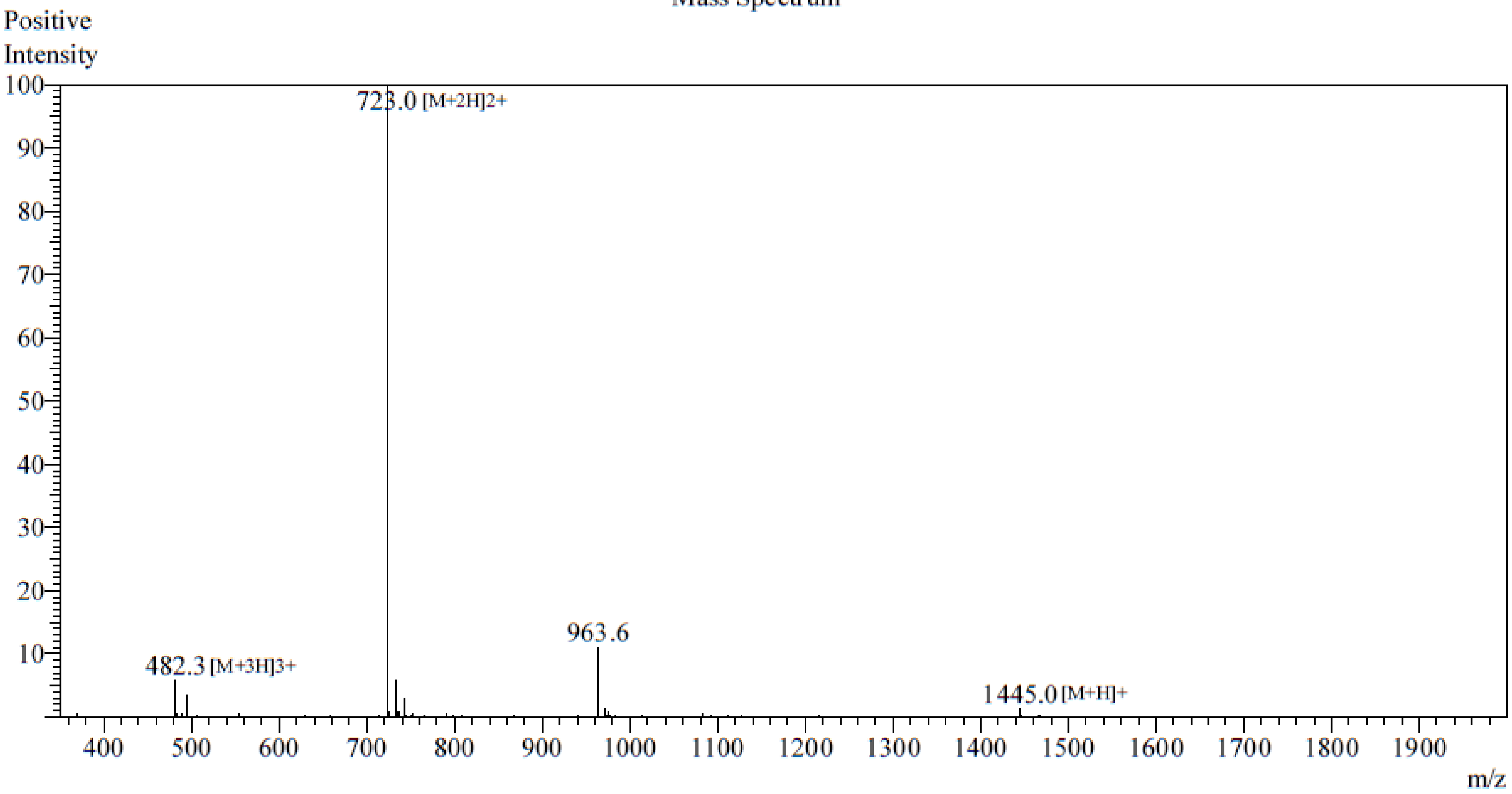

Sample Information	
Month-Day Processed :	02/10/22
Time Processed :	1:10:19 PM
Injection Volume :	0.2
Sample Name :	CIT5
Sample ID :	U6362HA280-9
Theoretical MW :	1443.82
Observed MW :	1444.0

Interface	:ESI
Nebulizing Gas Flow	:1.5L/min
CDL Temp	:250
Block Temp	:200

Equipment	: GK11010007
Interface Bias	: +4.5 kV
Drying Gas Flow	:5 L/min
T.Flow	:0.2 ml/min
B.conc	:50%H2O/50%M

Sample Name:CIT6
Sample ID:U6362HA280-11
Time Processed : 22:25:03
Month-Day-Year Processed : 02/19/2022

Pump A : 0.065% trifluoroacetic in 100% water (v/v)
Pump B : 0.05% trifluoroacetic in 100% acetonitrile (v/v)
Total Flow:1 ml/min
Wavelength:220 nm
<<LC Time Program>>

Time	Module	Command	Value
0.01	Pumps	Pump B Conc.	5
25.00	Pumps	Pump B Conc.	65
25.01	Pumps	Pump B Conc.	95
27.00	Pumps	Pump B Conc.	95
27.01	Pumps	Pump B Conc.	5
32.00	Pumps	Pump B Conc.	5
32.01	Controller	Stop	

<<Column Performance>>
<Detector A>
Column :Inertsil ODS-3 4.6 x 250 mm
Equipment:GK12010012

<Chromatogram>

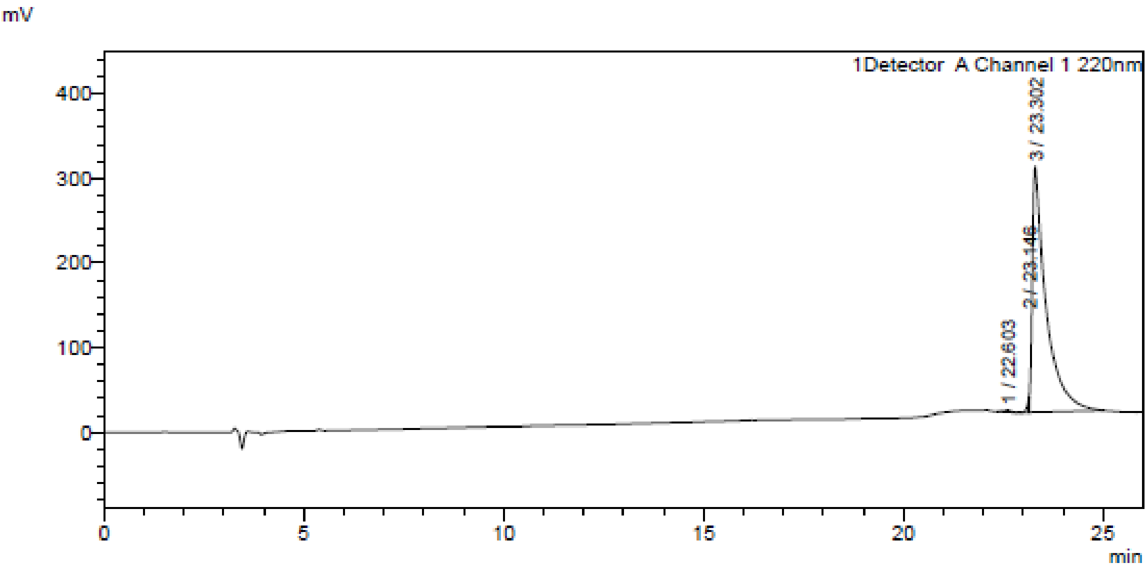

<Peak Table>

Detector A Channel 1 220nm

Peak#	Ret. Time	Area	Height	Area%
1	22.603	27614	2326	0.393
2	23.146	55315	15061	0.788
3	23.302	6937444	289952	98.819
Total		7020374	307339	100.000

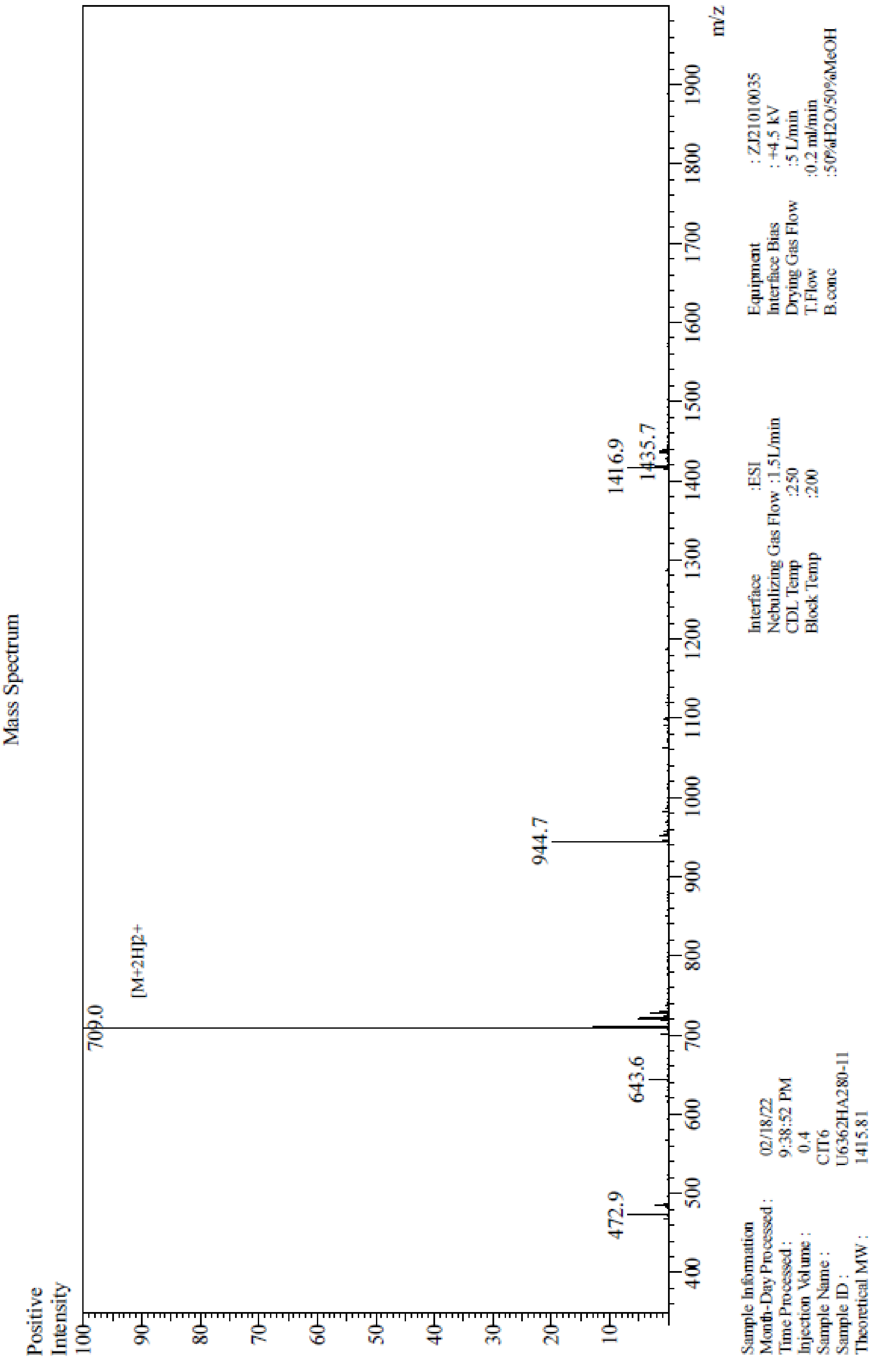

Mass Spectrum
Positive Intensity
709.0
[M+2H]2+
472.9
643.6
944.7
1416.9
1435.7
m/z
Sample Information
Month-Day Processed : 02/18/22
Time Processed : 9:38:52 PM
Injection Volume : 0.4
Sample Name : CIT6
Sample ID : U6362HA280-11
Theoretical MW : 1415.81
Observed MW : 1416.0
Interface :ESI
Nebulizing Gas Flow :1.5L/min
CDL Temp :250
Block Temp :200
Equipment : ZJ2101035
Interface Bias : +4.5 kV
Drying Gas Flow :5 L/min
T.Flow :0.2 ml/min
B.conc :50%H2O/50%MeOH

265

Sample Name : CIT7
Sample ID : U6362HA280-13
Time Processed : 13:12:16
Month-Day-Year Processed : 02/13/2022

Pump A : 0.065% trifluoroacetic in 100% water (v/v)
Pump B : 0.05% trifluoroacetic in 100% acetonitrile (v/v)
Total Flow:1 ml/min
Wavelength:220 nm

Time	Module	Command	Value
0.01	Pumps	B.Conc	5
25.00	Pumps	B.Conc	65
25.01	Pumps	B.Conc	95
27.00	Pumps	B.Conc	95
27.01	Pumps	B.Conc	5
35.00	Pumps	B.Conc	5
35.01	Controller	Stop	

<<Column Performance>>
<Detector A>
Column : Inertsil ODS-3 4.6 x 250 mm
Equipment: GK11010017

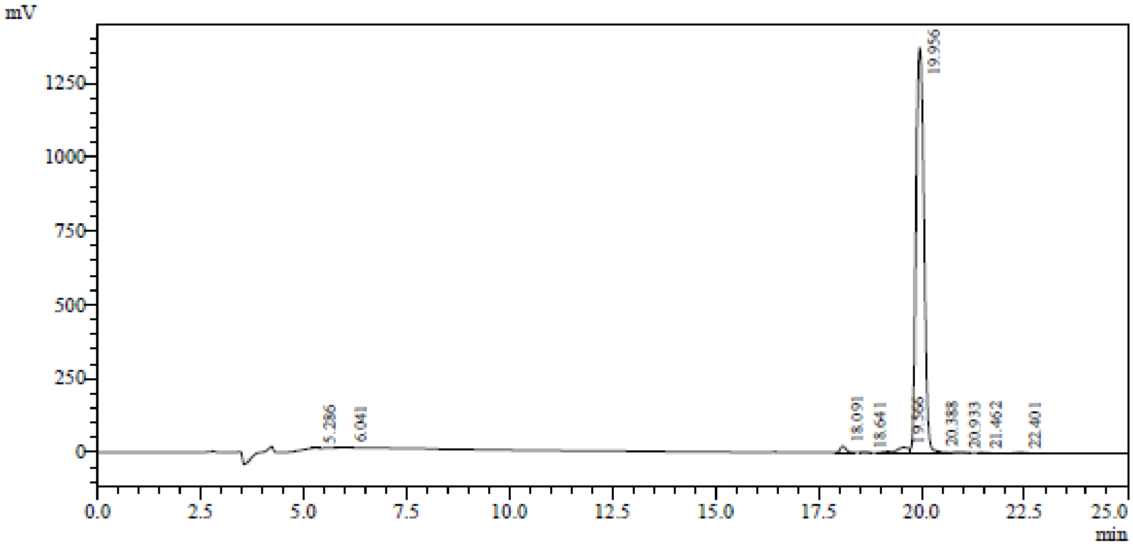

1 Detector A Channel 1 / 220nm

Peak Table

Detector A Channel 1 220nm

Peak#	Ret. Time	Area	Height	Area %
1	5.286	17309	3072	0.090
2	6.041	29944	1922	0.156
3	18.091	220507	22843	1.149
4	18.641	6113	798	0.032
5	19.566	396944	18323	2.069
6	19.956	18391949	1372380	95.842
7	20.388	40898	5710	0.213
8	20.933	29699	2158	0.155
9	21.462	16031	2163	0.084
10	22.401	40497	2688	0.211
Total		19189890	1432058	100.000

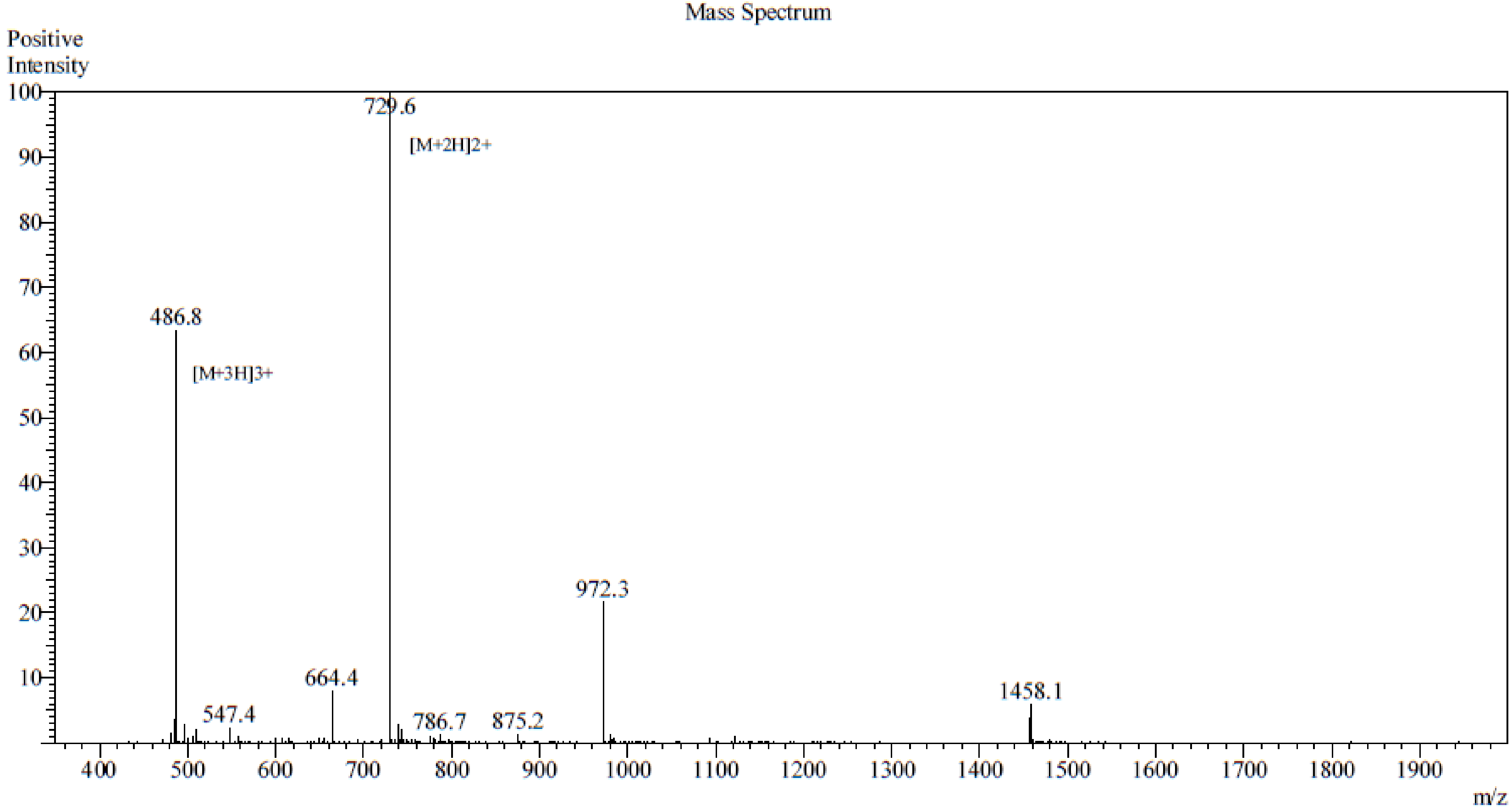

Sample Information		Interface	:ESI	Equipment	: GK11010007
Month-Day Processed :	02/11/22	Nebulizing Gas Flow	:1.5L/min	Interface Bias	: +4.5 kV
Time Processed :	7:08:21 PM	CDL Temp	:250	Drying Gas Flow	:5 L/min
Injection Volume :	0.4	Block Temp	:200	T.Flow	:0.2 ml/min
Sample Name :	CIT7			B.conc	:50%H2O/50%MeOH
Sample ID :	U6362HA280-13				
Theoretical MW :	1456.91				
Observed MW :	1457.2				

Sample Name :CIT8
Sample ID :U6362HA280-15
Time Processed :11:09:16 AM
Month-Day-Year Processed :02/18/2022

Pump A : 0.065% trifluoroacetic in 100% water (v/v)
Pump B : 0.05% trifluoroacetic in 100% acetonitrile (v/v)
Total Flow:1 ml/min
Wavelength:220 nm
<<LC Time Program>>

Time	Module	Command	Value
0.01	Pumps	Pump A B.Conc	5
25.00	Pumps	Pump A B.Conc	65
25.01	Pumps	Pump A B.Conc	95
27.00	Pumps	Pump A B.Conc	95
27.01	Pumps	Pump A B.Conc	5
35.00	Pumps	Pump A B.Conc	5
35.01	Controller	Stop	

<<Column Performance>>
<Detector A>
Column :Inertsil ODS-3 4.6 x 250 mm
Equipment: GR11010440

<Chromatogram>

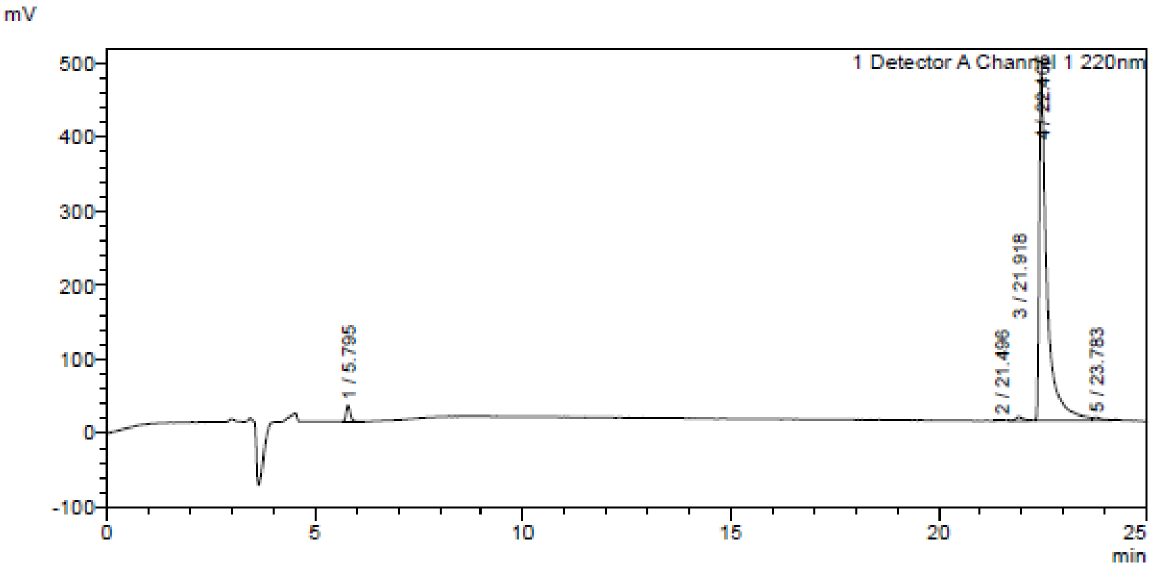

<Peak Table>

Detector A Channel 1 220nm

Peak#	Ret. Time	Area	Height	Area%
1	5.795	145896	21571	2.237
2	21.496	6734	801	0.103
3	21.918	72775	5576	1.116
4	22.465	6229754	470404	95.506
5	23.783	67736	4168	1.038
Total		6522895	502520	100.000

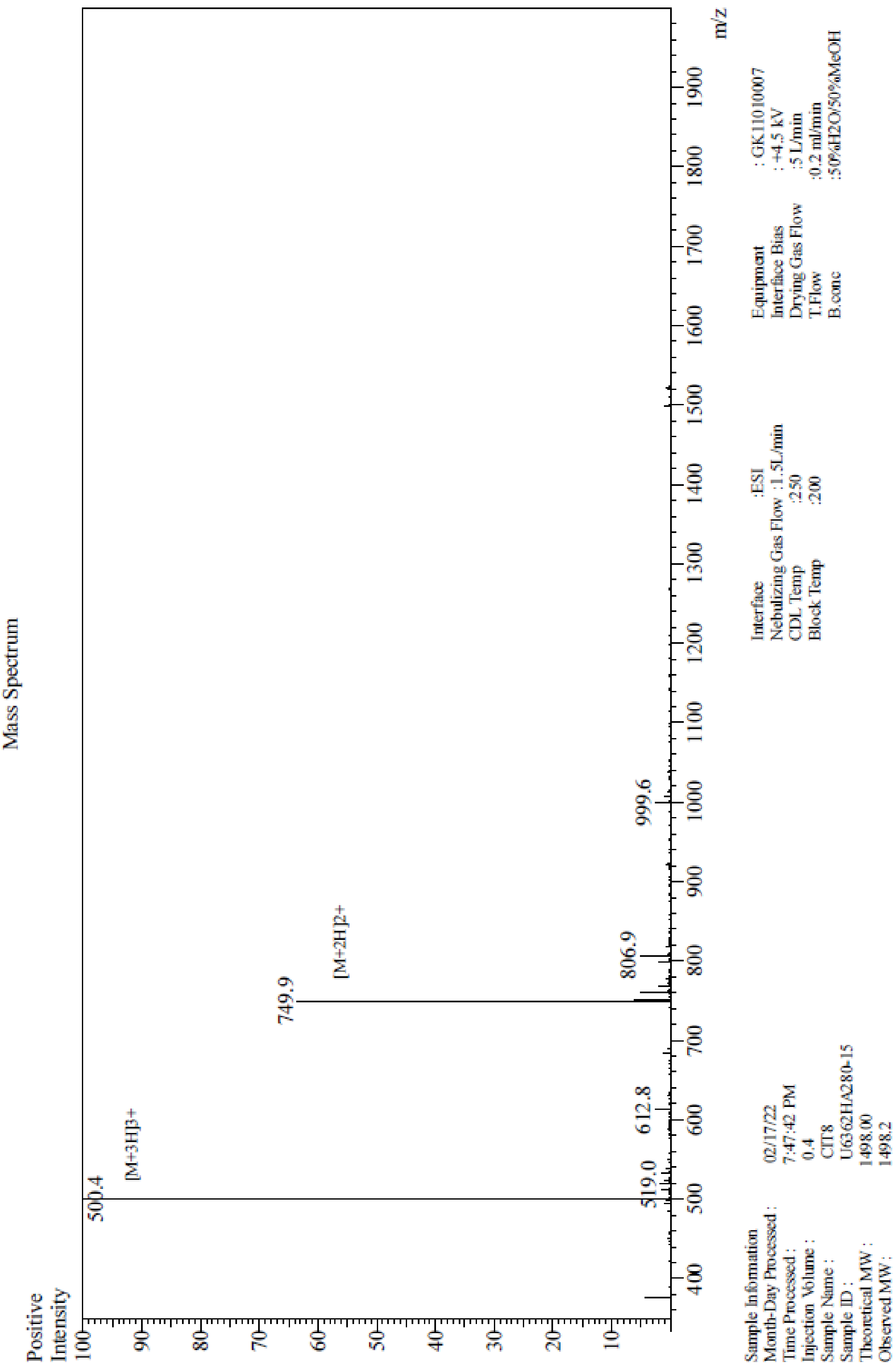
Mass Spectrum
Positive
Intensity
100
90
80
70
60
50
40
30
20
10
500.4
[M+3H]3+
519.0
612.8
749.9
[M+2H]2+
806.9
999.6
400
500
600
700
800
900
1000
1100
1200
1300
1400
1500
1600
1700
1800
1900
m/z
Sample Information
Month-Day Processed : 02/17/22
Time Processed : 7:47:42 PM
Injection Volume : 0.4
Sample Name : CIT8
Sample ID : U6362HA280-15
Theoretical MW : 1498.00
Observed MW : 1498.2
Interface :ESI
Nebulizing Gas Flow :1.5L/min
CDL Temp :250
Block Temp :200
Equipment : GK11010007
Interface Bias : +4.5 kV
Drying Gas Flow :5 L/min
T.Flow :0.2 ml/min
B.conc :50%H2O/50%MeOH

Sample Name : CIT2.1
Sample ID : U6970HC040-1
Time Processed : 9:25:03
Month-Day-Year Processed : 04/01/2022

Pump A : 0.065% trifluoroacetic in 100% water (v/v)
Pump B : 0.05% trifluoroacetic in 100% acetonitrile (v/v)
Total Flow:1 ml/min
Wavelength:220 nm

Time	Module	Command	Value
0.01	Pumps	B.Conc	5
25.00	Pumps	B.Conc	65
25.01	Pumps	B.Conc	95
27.00	Pumps	B.Conc	95
27.01	Pumps	B.Conc	5
35.00	Pumps	B.Conc	5
35.01	Controller	Stop	

<<Column Performance>>
<Detector A>
Column : Inertsil ODS-3 4.6 x 250 mm
Equipment: GK11010017

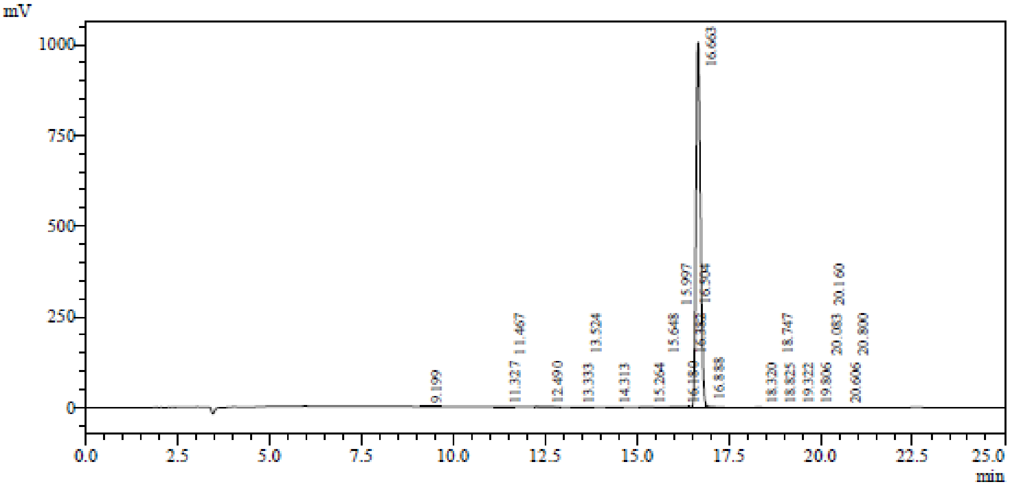

1 Detector A Channel 1 / 220nm

Peak Table

Detector A Channel 1 220nm

Peak#	Ret. Time	Area	Height	Area %
1	9.199	1908	99	0.022
2	11.327	2571	199	0.030
3	11.467	1347	178	0.016
4	12.490	10436	482	0.121
5	13.333	2519	328	0.029
6	13.524	2053	152	0.024
7	14.313	1243	106	0.014
8	15.264	4932	283	0.057
9	15.648	3562	367	0.041
10	15.997	21866	1639	0.254
11	16.180	17824	1699	0.207
12	16.382	14052	1720	0.163
13	16.504	9197	2492	0.107
14	16.663	8439200	1007004	98.072
15	16.888	43638	7592	0.507
16	18.320	10815	982	0.126
17	18.747	1348	155	0.016
18	18.825	1842	178	0.021
19	19.322	4196	260	0.049
20	19.806	2571	164	0.030
21	20.083	2091	241	0.024
22	20.160	3310	267	0.038
23	20.606	1422	146	0.017
24	20.800	1123	124	0.013

Peak#	Ret. Time	Area	Height	Area %
Total		8605068	1026859	100.000

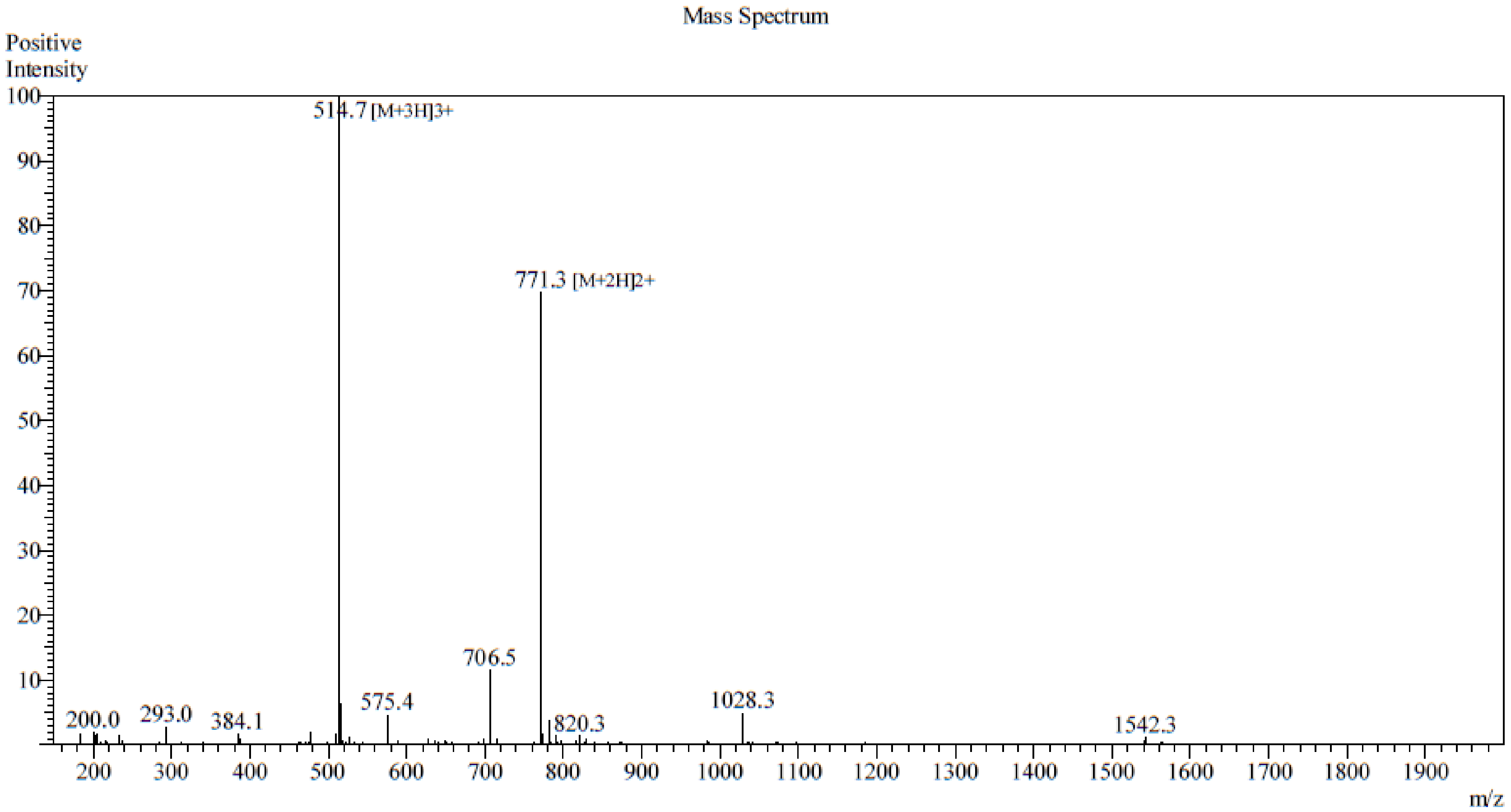

Sample Information
Month-Day Processed : 03/30/22
Time Processed : 8:54:19
Injection Volume : 0.2
Sample Name : CIT2.1
Sample ID : U6970HC040-1
Theoretical MW : 1541.07
Observed MW : 1541.1

Interface :ESI
Nebulizing Gas Flow :1.5L/min
CDL Temp :250
Block Temp :200

Equipment : ZJ21010035
Interface Bias : +4.5 kV
Drying Gas Flow :5 L/min
T.Flow :0.2 ml/min
B.conc :50%H2O/50%MeOH

Sample Name : CIT2.2
Sample ID : U6970HC040-3
Time Processed : 2:55:20 PM
Month-Day-Year Processed : 03/27/2022

Pump A : 0.065% trifluoroacetic in 100% water (v/v)
Pump B : 0.05% trifluoroacetic in 100% acetonitrile (v/v)
Total Flow:1 ml/min
Wavelength:220 nm

Time	Module	Command	Value
0.01	Pumps	B.Conc	5
25.00	Pumps	B.Conc	65
25.01	Pumps	B.Conc	95
27.00	Pumps	B.Conc	95
27.01	Pumps	B.Conc	5
35.00	Pumps	B.Conc	5
35.01	Controller	Stop	

<<Column Performance>>
<Detector A>
Column : Inertsil ODS-3 4.6 x 250 mm
Equipment: GK11010017

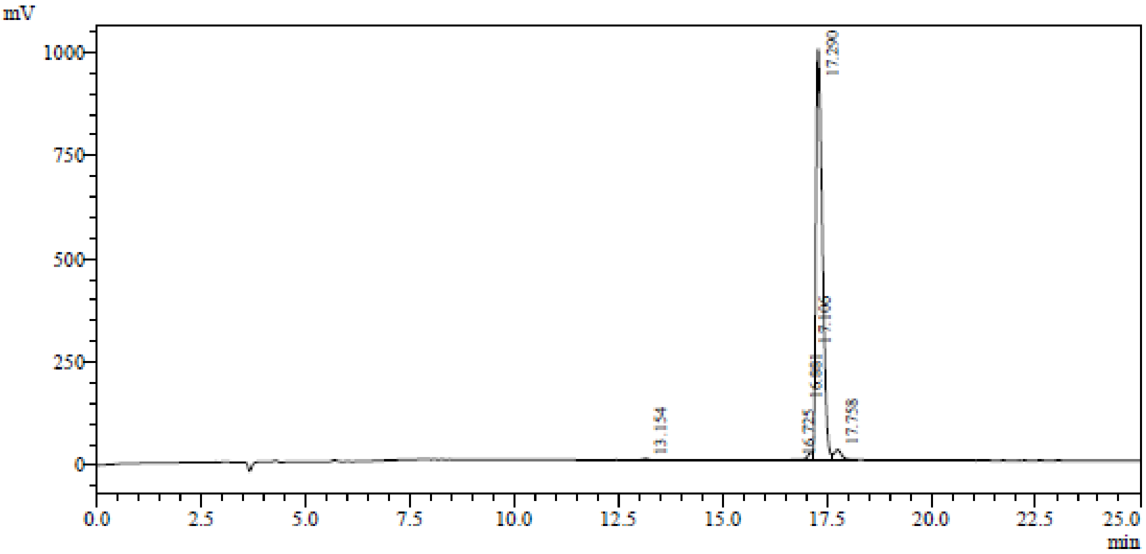

1 Detector A Channel 1 / 220nm

Peak Table

Detector A Channel 1 220nm

Peak#	Ret. Time	Area	Height	Area %
1	13.154	33152	3688	0.298
2	16.725	16423	1744	0.147
3	16.881	11400	1819	0.102
4	17.106	140157	18638	1.258
5	17.290	10590603	996074	95.083
6	17.758	346542	25117	3.111
Total		11138277	1047079	100.000

276

Mass Spectrum

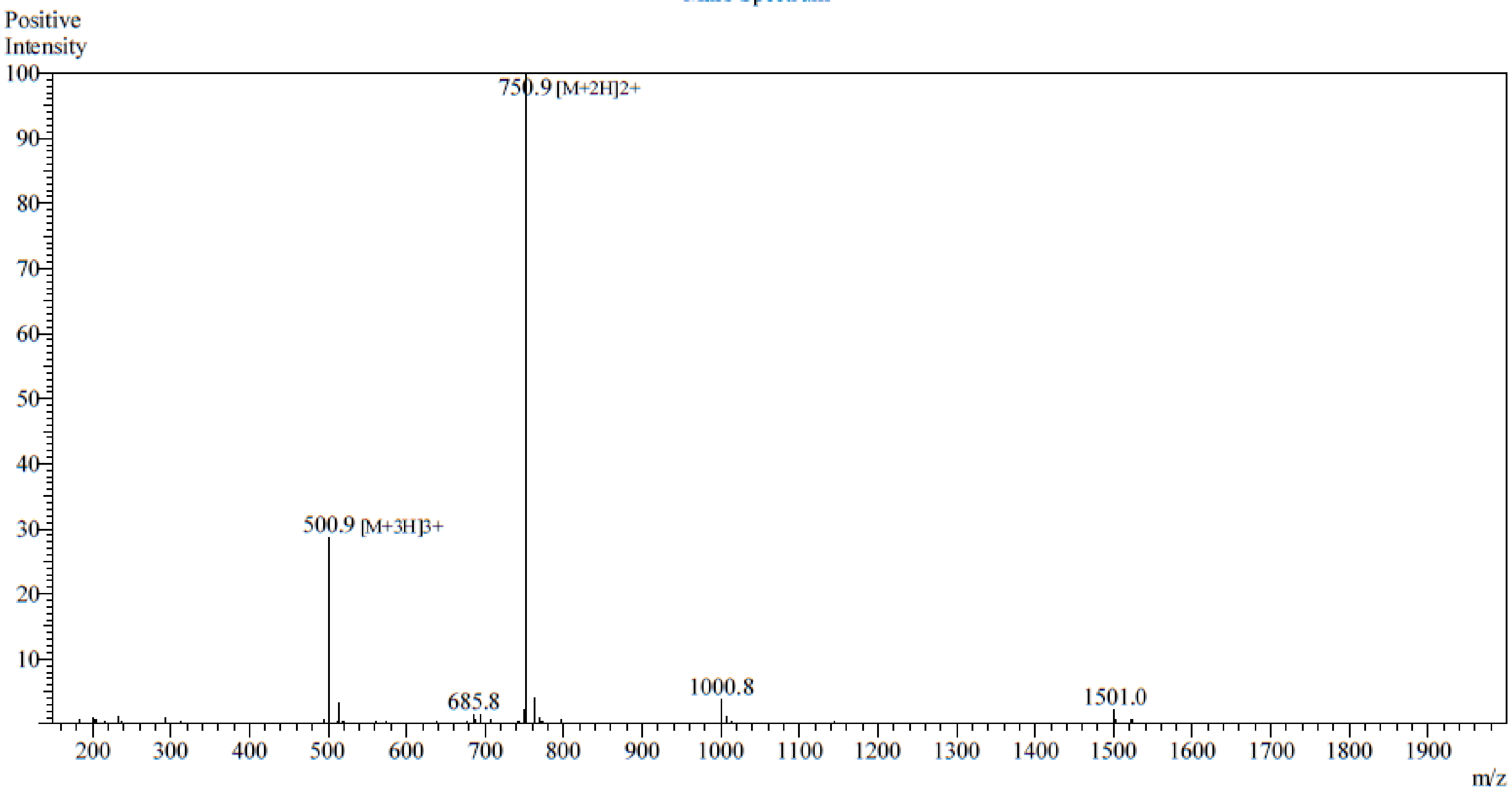

Sample Information
Month-Day Processed : 03/25/22
Time Processed : 19:37:23
Injection Volume : 0.2
Sample Name : CIT2.2
Sample ID : U6970HC040-3
Theoretical MW : 1499.97
Observed MW : 1499.8

Interface :ESI
Nebulizing Gas Flow :1.5L/min
CDL Temp :250
Block Temp :200

Equipment : ZJ21010035
Interface Bias : +4.5 kV
Drying Gas Flow :5 L/min
T.Flow :0.2 ml/min
B.conc :50%H2O/50%MeOH

Materials and Methods

In silico data collection and processing

We parsed these sequences from fasta files utilizing Python and the Biopython library (2). Our preprocessing steps included deduplication and aggregation into pandas DataFrames, with sequences being converted to uppercase to ensure uniformity. We excluded sequences with non-standard amino acid designations specifically X (any amino acid, unknown or unspecified), B (asparagine or aspartic acid, ambiguous), Z (glutamine or glutamic acid, ambiguous), J (leucine or isoleucine, ambiguous), U (selenocysteine), and O (pyrrolysine).

We calculated the molecular weight, grand average of hydropathicity (GRAVY), helicity, hydrophobicity, hydrophobic moment and topological surface area (TPSA) for each peptide sequence. We also calculated the 5-dimensional physico-chemical property descriptors (PCP descriptors) derived by the multidimensional scaling of 237 physical-chemical properties (3). These calculations were made using the Biopython library (2) and the peptides.py library (https://peptides.readthedocs.io/).

Bacterial strains growth conditions and peptide synthesis

We used several *S. aureus* strains with different varying sensitivity to antibiotics. We included *S. aureus* MRSA (strain MW2), vancomycin intermediate-resistant *S. aureus* (VISA) (strains AR0215, AR0216, AR0217, AR0219 and AR0225), vancomycin-resistant *S. aureus* (VRSA) (strain VRS1), and various *S. aureus* clinical isolates (BF-1 to BF-11). See Supplemental Table 9 for a complete strain list. For the growth of *S. aureus* bacteria, we used tryptic soy broth (TSB) (BD, Franklin Lakes, NJ, USA).

Synthesis of peptides

We synthesized CIT-derived peptides by solid-phase chemistry (GenScript Inc., Piscataway, NJ, USA). CIT-derived peptides had a purity of 95% or more (peptide characterization data is provided in the Supplementary information).

Minimal inhibitory concentration (MIC) assay

To determine the minimum concentration of peptide that inhibits the growth of bacteria, termed as the minimal inhibitory concentration (MIC), we used the broth microdilution method described by the Clinical and Laboratory Standards Institute (4). We made serial dilutions of 10× concentrated peptides (10 µl) in duplicate in 96-well plates (Cat no. 3595, Corning, NY, USA). We added 90 µl of logarithmic-phase bacteria at 1×10^6 CFU/ml (in TSB medium) to the peptides and then incubated the plates at 37°C for 18 hours. We determined the MIC using OD_{600} measurements taken on a Spectra Max i3x spectrophotometer (Molecular Devices, CA, USA). To investigate the activity of peptides in various salt concentrations, we included physiologically relevant salt and serum concentrations in the TSB medium. Accordingly, we added 150 mM NaCl, 2.5 mM $CaCl_2$, 8 µM $ZnSO_4$, 1 mM $MgSO_4$, and 5-10% human serum to our peptide MIC assay.

S. aureus *persister cell and time-kill assays*

For the generation of antibiotic-induced MRSA and VRSA persister cells, we followed an established protocol (5). In brief, we grew 25 ml of *S. aureus* strains MW2 or VRS1 cultures to stationary phase and then treated the cells with gentamicin at 20 µg/ml for 4 additional hours. We washed the bacterial cultures with the same volume of phosphate-buffered saline (PBS) three times and adjusted the culture to ~1×10^8 CFU/ml (for *S. aureus* MW2) and ~1×10^6 CFU/ml (for *S.*

aureus VRS1) in PBS. To assess the killing kinetics of CIT-8 in persister cells, we added 1 ml of cell suspension to the wells of a 2-ml deep-well assay block (Cat no. 3960, Corning, NY, USA) containing 10× MIC or 1× MIC of CIT-8. We also included ciprofloxacin (at 10 µg/ml) for for *S. aureus* MW2 and linezolid (100 µg/ml) for *S. aureus* VRS1 as antibiotic controls. Additionally, we included bithionol (at 10 µg/ml) as a positive control, known for its ability to kill stationary phase persister cells (6). We incubated the plates at 37°C, with shaking at 225 rpm. At specific times (0, 15, 30, 60, and 120 min), we collected 10 µl samples, diluted them serially, and then plated them on tryptic soy agar (TSA) (BD Difco, NJ, USA) plates. We incubated the TSA plates for 18 hours at 37°C for colony counting. We performed these experiments in duplicate. As a comparison, we prepared the exponential cells of *S. aureus* MW2 and VRS1 in PBS and analyzed their killing kinetics in the same manner as described for persister cells.

Biofilm viability assay on solid support

We generated gentamicin-induced MRSA and VRSA persister cells as detailed above. We diluted the persister cell culture 1:200 with TSB supplemented with 0.2% glucose (7). To generate biofilms on substrates, we added 1 ml of diluted bacterial culture over a 13-mm diameter Millipore mixed cellulose ester membrane (Cat no. HAWP01300, Millipore, MA, USA) placed at the bottom of a 12-well plate (Cat no. 353043, Falcon, MA, USA) and incubated statically at 37°C for 24 h. To remove planktonic cells, we washed the membranes two times and transferred them to a new 12-well plate. Next, we added 1 ml of PBS with 10× MICs of peptide and antibiotics to each well and incubated the plates statically at 37°C for another 24 h. We then washed the membranes two times with PBS, placed in 1 ml PBS, and sonicated in a FS 30 ultrasonic bath (Fisher Scientific, MA, USA) for 10 min. We serially diluted the sonicated samples with PBS in a 96-well plate, spot-

plated onto TSA plates, and incubated plates at 37°C overnight. The next day, we counted the viable bacterial colonies.

Prevention of S. aureus MW2 *biofilm formation*

We evaluated the ability of the citropin 1.1-derived peptides to inhibit biofilm formation following an established protocol with modifications (5). In short, we prepared exponential cultures of *S. aureus* MW2 (adjusted to OD_{600} = 0.01) in TSB medium (supplemented with 0.2% glucose) from overnight cultures. We added 90 µl of the bacterial culture to 10 µl of serially diluted 10× peptide solution in flat-bottomed 96-well polystyrene microtiter plates (Cat no. Corning 3595, NY, USA) and then incubated the plates at 37°C for 24 hours. We used TSB medium containing bacteria with water as the positive control, while TSB media with sterile water served as the negative control. After incubation, we carefully removed the TSB medium and washed the wells with PBS (Gibco, MD, USA) to remove loosely attached planktonic cells. We quantified the biomass by staining the biofilms with crystal violet following an established protocol and quantitated the concentration of peptide that inhibits 50% of biofilm formation ($MBIC_{50}$)(8). We measured the live cell count of the biofilms using XTT dye [2,3-bis(2-methyloxy-4-nitro-5-sulfophenyl)-2H-tertazolium-5-carboxanilide] (ATCC, VA, USA). We calculated the percentage of biofilm growth by normalizing to biofilm growth in wells containing bacteria without peptide treatment(8).

Disruption of S. aureus *MW2 established biofilms*

We evaluated the ability of the citropin 1.1-derived peptides to disrupt established biofilms following an established protocol with modifications (5). We prepared exponential cultures of *S.*

aureus MW2 (adjusted to 1 × 10^6 CFU/ml) in TSB medium (supplemented with 0.2% glucose) from overnight grown cultures. We added 100 µl of bacterial culture to flat-bottomed 96-well polystyrene microtiter plates (Cat no. 3595, Corning, NY, USA). We then incubated the plates at 37°C for 24 hours in static conditions to allow biofilm formation. After incubation, we carefully pipetted out the TSB medium and washed the wells with PBS (Gibco, MD, USA) to remove loosely attached planktonic cells. We treated the established biofilms in each well with 10 µl of serially diluted 10× peptide solution, followed by the addition of 90 µl TSB medium (supplemented with 0.2% glucose). We incubated the plates for another 24 h at 37°C before processing biomass and live-cell contents. We calculated the concentration of peptide that disrupts 50% of established biofilms ($MBEC_{50}$).

Fluorescence microscopy of CIT-8-treated S. aureus *MW2 established biofilms*

We prepared exponential phase cultures of *S. aureus* MW2 and adjusted the bacterial count to 1 × 10^6 CFU/ml in fresh TSB medium. To establish biofilms, we added 200 µl of the bacterial culture to the chambers of Millicell EZ Slides (Millipore Sigma, MA, USA) and maintained them at 37°C for 24 hours under static conditions (8). After incubation, we carefully removed the TSB medium from the wells and then washed the Millicell wells with PBS (Gibco, MD, USA) to remove loosely attached planktonic cells. We treated the established biofilms in each well with 20 µl of CIT-8 peptide (at 10× MIC) followed by the addition of 180 µl TSB medium and incubated the Millicell for an additional 24 h at 37°C. After incubation, we washed the Millicell carefully with PBS (Gibco, MD, USA) to remove the planktonic cells and stained the biofilms with 50 µl of staining reagent from a LIVE/DEAD kit (Life Technologies, OR, USA) according to the manufacturer's instructions. Finally, we visualized the biofilms using a fluorescent microscope

Nikon ECLIPSE Ti (Melville, NY, U.S.A). We processed the images using the NIS-Elements AR 4.00.07 software (Nikon, Tokyo, Japan).

Hemolysis of human red blood cells (hRBCs)

We evaluated the ability of peptides to cause hemoglobin leakage in human red blood cells using a previously described method (9). Briefly, we washed human erythrocytes (Rockland Immunochemicals, PA, USA) three times in an equal volume PBS and resuspended them as a 4% hRBC solution. We added 100 µl of the blood cells to 100 µl of 2× peptide solution in a 96-well microtiter plate (Cat no. 3595, Corning, NY, USA) and incubated the plates at 37°C for 1 h. Next, we centrifuged the plates at 500× g for 5 min, transferred 100 µl of the supernatant fraction into a fresh 96-well plate, and read the absorbance at 540 nm. We calculated the percent hemolysis, considering 100% hemolysis caused by 1% Triton X-100 and 0% hemolysis in PBS, using the formula: (A_{540} nm in the peptide solution − A_{540} nm in PBS) / (A_{540} nm of 1% Triton X-100 treated sample − A_{540} nm in PBS) × 100.

Mammalian cell cytotoxicity assays

We used liver-derived HepG2 cells (American Type Culture Collection, Manassas, VA, USA) to evaluate the cytotoxicity of AMP based on an established protocol (9). We maintained the HepG2 cells at 37°C in 5% CO_2 in Dulbecco's Modified Eagle Medium (DMEM) (Gibco, MA, USA) supplemented with 10% fetal bovine serum (FBS) (Gibco, MD, USA) and 1% penicillin/streptomycin (Gibco, MD, USA). We harvested and resuspended cells in fresh DMEM medium and distributed 1×10^6 cells (in 50 µl) into a 96-well plate containing 50 µl of serially diluted AMPs in serum- and antibiotic-free DMEM and incubated the plates at 37°C in 5% CO_2

for 24 h. Before the end of the incubation period (at 20 h), we added 10 µl of 2-(4-iodophenyl)-3-(4-nitrophenyl)-5-(2,4-disulfophenyl)-2H-tetrazolium (WST-1) (Roche, Mannheim, Germany) to each well and monitored the reduction of WST-1 at 450 nm using a SpectraMax i3x (Molecular Devices, San Jose, CA, USA). We performed all assays in triplicate and calculated the percentage of cell survival.

Circular dichroism (CD)

We recorded CIT-8 (0.2 mg/ml) CD spectra in the presence or absence of 50 mM sodium dodecyl sulfate (SDS) using a Jasco J-815 Circular Dichroism (CD) Spectropolarimeter (Jasco, OK, USA) using a previously described method (10). The samples were placed in a thermostat cell holder maintained at 25°C in a quartz cell with a path length of 1 mm. The CD data were expressed as the mean residue ellipticity. We obtained the baseline scans using the same parameters for samples containing buffer, micelles, and vesicles only, and further subtracted from the respective data scans with peptide containing samples.

Nuclear magnetic resonance (NMR)

We used two-dimensional nuclear magnetic resonance (2D-NMR) spectroscopy to investigate the membrane targeting mechanism of CIT-8 based on a previously published method with minor modifications (11). The NMR data was measured at 25ºC using a Bruker Avance II 500 MHz NMR spectrometer (Bruker, MA, USA) with 600 µl of 2 mM CIT-8 dissolved in 80 mM deuterated SDS (SDS-d25; pH 5.5). We collected the homonuclear 2D- total correlation spectroscopy (TOCSY) with 80 ms mixing time and nuclear overhauser effect spectroscopy (NOESY) spectra with 300 ms mixing time, 5000 Hz spectral widths in both dimensions and 512

increments in the indirect dimension. We acquired the natural abundance 2D- ^{13}C- heteronuclear single quantum coherence (HSQC) and ^{13}C-HSQCTOCSY spectra with 128 scans to obtain ^{13}C chemical shifts, which were used to derive the peptide dihedral angles using TALOS+ (12). We acquired and processed all NMR data using the Topspin software version 3.2 (Bruker, MA, USA) and analyzed these data using Sparky version 3.115 (UCSF, CA, USA). A semi-automated assignment of NOESY cross-peaks was performed in the CYANA version 3.98.15 (13). All assigned peaks were manually verified in Sparky for accuracy. We performed the initial peptide structure calculation using 254 NOE-derived distance restraints and 22 dihedral angles. In the final run, we complemented with 14 hydrogen bond restraints, which were supported by the NOESY patterns. We used 200 structures in the final calculation and took the 20 lowest-energy structures as the CIT-8 structure ensemble.

Molecular dynamics (MD) simulation

We performed MD simulations in membranes composed of DOPC (1,2-Dioleoyl-sn-glycero-3-phosphocholine): DOPG (Dioleoyl phosphatidylglycerol) (DOPC:DOPG = 7:3). Each membrane bilayer was made of 128 molecules (6). We constructed the starting structure of CIT-8 using the Alphafold2 Colab server(14). We placed CIT-8 at least 1.5 nm from the membrane upper leaflet in parallel to the upper membrane system. We performed each simulation run in two stages(15). In the first stage, the membrane-peptide system as developed above were converted into Coarse Grained (CG) models using the Charmm-GUI server and simulated for 500 ns (16). In the second stage, frames corresponding to the important events during the CG simulation run, such as the initiation of membrane-peptide binding and complete membrane-binding after 500 ns, were converted into all-atom models and further simulated for 2 ns. We performed the CG-MD runs

using the Martini-22p forcefield and all-atom simulations with Charmm-36 forcefield(16). We carried out all the simulation runs in GROMACS 2020.1-1 simulation software package and visualized the data obtained using two software packages: the VMD (17) platform and the trial version of BIOVIA Discovery Studio Visualizer (Accelreys Inc., SD, USA).

Membrane depolarization

We measured bacterial membrane potential using an established protocol with minor modifications (9). We prepared an exponential phase culture of *S. aureus* MW2 in fresh TSB medium, washed the culture two times in PBS, and resuspended it in a double volume of PBS. To energize the bacterial cells, we added 25 mM of glucose for 15 mins at 37°C, followed by 500 nM of DiBAC4(3) (bis-(1,3-dibutylbarbituric acid) trimethine oxonol) (Thermo Fisher Scientific, ON, Canada). DiBAC4(3) is a potential-sensitive probe that enters depolarized cells, binds to intracellular proteins or membranes, and exhibits enhanced fluorescence. We distributed 90 µl of this bacterial culture per well in a black, clear bottomed 96-well plate (Cat. no. 3904, Corning, NY, USA) and monitored the fluorescence using a SpectraMax i3x (Molecular Devices, CA, USA) for 20 min, with excitation at 485 nm and emission at 520 nm, until the baseline stabilized. Then we added 10 µl of serially diluted peptide solutions and recorded the fluorescence for another 40 min. We included triton X-100 (1%) as a positive control.

SYTOX-based cell membrane permeability assay

We generated *S. aureus* MW2 exponential cells (as described above) and diluted them in PBS to OD_{600}=0.2. We added SYTOX Green (Molecular Probes, Life Technologies, Oregon, USA) to the diluted cell suspension to a final concentration of 5 µM and incubated them for 30

min at room temperature in the dark based on a previously established protocol (8). We added 90 µl of the dye/bacteria mixture to 10 µl of serially diluted 10× peptide concentration in black, 96-well plates (Cat. no. 3904, Corning, NY, USA) and incubated the plates for 1 hour at room temperature. We measured the fluorescence with excitation and emission wavelengths of 485 nm and 525 nm, respectively in endpoint assay mode using a SpectraMax i3x (Molecular Devices, CA, USA) fluorescence reader.

Propidium iodide-based membrane permeability

We used a propidium iodide (Thermo Fisher Scientific, MA, USA) fluorescence-based bacterial permeation assay to establish membrane-oriented interactions with the designed peptides based on a previously established protocol (8). We prepared exponential phase *S. aureus* MW2 bacteria and diluted to OD_{600} = 0.4 in PBS. We added PI dye (final concentration = 2 µM) to the bacteria in 1:10 serial dilutions in black, 96-well plates (Cat. no. 3904, Corning, NY, USA) and incubated the plates for 1 hour at room temperature. We then measured the fluorescence with an excitation wavelength of 584 nm and an emission wavelength of 620 nm in endpoint assay mode using a SpectraMax i3x (Molecular Devices, CA, USA) fluorescence reader.

ATP release assay

To determine the ATP leakage potential of the peptides, we employed a luciferase-based assay(18). In brief, we washed exponential phase of *S. aureus* MW2 bacteria three times with PBS (pH 7.4) and adjusted the washed cells to OD_{600} = 0.4 in PBS. Following treatment of the bacteria with 32 µg/ml of CIT-8 peptide at 37°C for 30 min, we centrifuged the cells at 14,000× g for 5 min. We transferred 50 µl of each supernatant fraction to black, clear-bottom, 96-well plates

containing 50 µl BacTiter-Glo reagent (Promega, WI, USA) and measured plate luminescence as previously described (18) after 5 min of incubation at room temperature.

Cryo-electron microscopy (cryo-EM)

We statically treated 10 ml of an exponential-phase *S. aureus* MW2 culture (OD_{600} = 0.4, washed and diluted in PBS) with 20× MIC of CIT-8 for 1h at 37°C. For imaging, we vitrified the bacteria cells using Leica EM-GP2® plunger (Leica Microsystems, Wetzlar, Germany) on R2×2 Quantifoil® carbon holey film (Micro Tools GmbH, Jena, Germany) grids as previously described (19). Briefly, we applied a 4 µl of bacterial suspensions to the grids pre-cleaned in a Gatan Solarus 950 plasma cleaner (Gatan, Inc, USA), blotted with filter paper, and plunged into liquid ethane. We stored the frozen grids under liquid nitrogen until used for microscopy. We then transferred the grids into a JEM 2200FS electron microscope (JEOL Ltd, Akishima, Japan) operating at 200 keV and equipped with a field emission gun, in-column electron energy filter (omega type) and a DE20 direct electron detector camera (Direct Electron Inc., CA, USA). We used a 20 eV energy slit width during data acquisition. Total electron dose/image was ~15 electrons/$Å^2$. Image pixel size was 3.66 Å on the specimen scale.

Scanning electron microscopy (SEM)

We followed an established protocol with minor modification to perform SEM imaging of *S. aureus* MW2 bacteria treated with CIT-8 peptide (20). In short, we statically treated 2 ml of an exponential-phase *S. aureus* MW2 culture (OD_{600} = 0.4, washed and diluted in PBS) with 10× MIC of CIT-8 for 1h at 37°C. After that, we spun the bacterial cultures at 13000× g for 10 mins at room temperature. We fixed the bacteria using 50 µl of fixing solution containing 2.5%

glutaraldehyde in PBS buffer, pH 7.3, for over 1 h (room temperature). After fixation, we applied a volume of 20 µl sample droplet on to an (3-Aminopropyl) triethoxysilane (APTES) functionalized Si (Silicon) (100) surface for incubation for 1 hour. Then we washed the Si substrate thoroughly in PBS, pH 7.3. For dehydration, we sequentially subjected the substrate to 20%, 40%, 60%, 90% and 100% ethanol solution (v/v in water) for 5 min each. We placed the samples in a tert-butyl alcohol (50% v/v in ethanol) for 5 min and let the sample dry in the air. Next, we used double-sided carbon conductive tape (Cat no. 16084-7, Ted Pella Inc., CA, USA) for immobilizing the wafer on an aluminum SEM sample holder. To enhance the SEM image contract, we coated the samples with a thin Pt/Pd film of 5 nm with a magnetron 208HR High Resolution sputtering Coater (Ted Pella Inc., CA, USA) and visualized the sample in a Nava Nano SEM 230 (FEI, OR, USA) with a work distance of 5 mm. We conducted all tests at room temperature and in a high vacuum (2E-6 Torr). In the process of imaging, we set the e-beam diameter at 3 nm and the acceleration high voltage at 5 kV.

Use of S. aureus *transposon mutants*

We used *S. aureus* transposon mutants from the Nebraska Transposon Mutant Library (NTML)(21). The library has a collection of 1,952 strains, each containing a single mutation within a nonessential gene of USA300, an epidemic community-associated MRSA (CA-MRSA) isolate (21). We selected a transposon insertion mutant from the NTML library in the *fmtc* gene, which catalyzes the transfer of a lysyl group from L-lysyl-tRNA to membrane-bound phosphatidylglycerol (PG) to produce lysylphosphatidylglycerol (LPG) (11). LPG imparts a less-negative charge to the overall membrane potential and thus contributes to bacterial virulence by contributing a resistance mechanism against cationic AMPs (11).

RNAseq assays

For RNAseq studies, we followed the recommended procedure by Novogene (Sacramento, CA, USA) (22). In brief, we used exponential cultures of *S. aureus* MW2 grown in TSB medium adjusted to OD_{600}=0.4. We treated 10 ml of the bacterial culture with 0.5× MIC of CIT-8 or water (as a negative control) for 30 min at 37°C (180 rpm). After that, we washed the bacteria three times with PBS and stored the pellet at -80°C. We isolated total RNA from the pellet and checked the purity using the NanoPhotometer® spectrophotometer (Implen, CA, USA). We assessed RNA integrity and concentration using the RNA Nano 6000 Assay Kit following manufacturer instructions in a Bioanalyzer 2100 system (Agilent Technologies, CA, USA). We evaluated RNA degradation and contamination on 1% agarose gels. To prepare libraries for transcriptome sequencing, we used 1 µg RNA per sample as input material and generated sequencing libraries using NEBNext® Ultra™ RNA Library Prep Kit for Illumina® (NEB, MA, USA) following manufacturer's recommendations and added index codes to attribute sequences to each sample. Briefly, we purified mRNA from total RNA using poly-T oligo-attached magnetic beads, followed by fragmentation using divalent cations under elevated temperature in NEBNext First Strand Synthesis Reaction Buffer (5×). To synthesize cDNA, we used random hexamer primers and M-MuLV Reverse Transcriptase (RNase H-) to create the first strand, followed by DNA Polymerase I and RNase H to create the second strand (23). The remaining overhangs were converted into blunt ends using exonuclease/polymerase activities. We adenylated the 3' ends of the DNA fragments, and further ligated NEBNext Adaptors with hairpin loop structures to prepare for hybridization. To select cDNA fragments of preferred length (150-200 bp), we purified the whole library fragments with AMPure XP system (Beckman Coulter, CA, USA). Next, to the size-selected, adaptor ligated cDNA, we added 3 µl USER Enzyme (NEB, MA, USA) at 37°C for 15

min followed by 5 min at 95°C before carrying out the polymerase chain reaction (PCR). We performed the PCR with Phusion High-Fidelity DNA polymerase, Universal PCR primers, and Index (X) Primer. Finally, we purified the PCR products with an AMPure XP system (Beckman Coulter, CA, USA) and library quality was assessed on the Agilent Bioanalyzer 2100 system (Agilent Technologies, CA, USA).

Metabolomic analysis

For the metabolomics sample preparation, we followed an established protocol with minor modifications (8). In short, we used exponential-phase cultures of *S. aureus* MW2 grown in TSB medium adjusted to OD_{600}=0.5. We treated 10 ml of the bacterial culture with 2× the determined MIC of peptide CIT-8 (or water as a negative control) for 30 min at 37°C (n=3, technical replicates). After that, we washed the bacterial pellet three times with PBS and stored the pellet at -20°C. Next, to each cell pellet, we added 0.2 ml of 2-propanol: 100 mM ammonium bicarbonate (Millipore Sigma, MA, USA), pH 7.4 (1:1 v/v) and sonicated the cells. For spiking, we added 20 µl of stable isotope to each sample and vortexed the mixture. Subsequently, we added 1.0 ml methanol for deproteinization and cooled the mixture at -20°C for 10 min. After centrifugation for 10 min at 14,000× g at 4ºC, we transferred the supernatant fraction into glass tubes and dried the supernate under a stream of nitrogen at 40°C. Finally, we dissolved the residues in 100 µl of water and injected 3 µl of this solution into an LC-MS/MS 8060 system (Shimadzu Scientific Inc, MD, USA), equipped with a DUIS source operated in both positive and negative electrospray ionization modes. For liquid chromatographic analysis, we used the Nexera UPLC system (Shimadzu Scientific Inc, MD, USA). We quantified primary metabolites (~150) following an established LC-MS/MS method (24).

In vivo murine skin infection and treatment

We used a topical skin-abraded murine infection model with minor modifications to test the *in vivo* efficacy of the CIT-8 peptide (25). We performed all mouse studies following the protocol approved by the Institutional Animal Care and Use Committee. In brief, we anesthetized female C57BL/6 mice (6 weeks) (Jackson Laboratory, Bar Harbor, ME, USA) with ketamine-xylazine (90 mg/kg ketamine and 10 mg/kg xylazine) via intraperitoneal injection. We shaved the dorsal side of the mice in the middle of the back (area=2 cm^2). To disrupt skin integrity, we tape-stripped with Tensoplast® seven times in succession, replacing the tape each time, resulting in visibly damaged skin. We infected the damaged skin area with 1×10^7 CFU of *S. aureus* MW2 (exponential phase bacteria in PBS) with an inoculum of 50 µl using a pipette tip. Ten minutes (prophylactic model) and 24 h (established model) after bacterial inoculation, we applied 30 mg of 1 and 2% (w/w) CIT-8 ointment in a white petroleum jelly base (Cat no. 19-090-843, Fisher Scientific, MA, USA) to the skin. We also included animal groups treated with only petroleum jelly or with 2% commercial mupirocin ointment as negative and positive controls, respectively. For pain management, we used the analgesic buprenorphine SR (0.05 mg/kg).

After 24 h of treatment, we euthanized the animals, separated the skin from the underlying fascia and muscle tissue, and excised about 2 cm^2 of skin from the infected area. We ground the skin in 1ml of PBS buffer (Gibco, MD, USA). We serially diluted 100 µl of the solution in PBS and plated it on Remel™ TSA plates (Fisher, MA, USA) to estimate viable colony counts after 18 h of incubation at 37ºC. Further, we added 10 µl of EDTA-Free, protease inhibitor cocktail Set III (Millipore Sigma, MA, USA) to 500 µl of the ground skin PBS solution for cytokine estimation. The Cytokine Core, LLC (Indianapolis, IN, USA) performed the cytokine concentration analysis

based on their in-house protocol on a Luminex® MagPix™ system(26). We also performed the histopathology of the murine skin by staining with haematoxylin and eosin (H&E) and Gram stain.

References

1. Kyte J, and Doolittle RF. A simple method for displaying the hydropathic character of a protein. *J Mol Biol.* 1982;157(1):105-32.
2. Cock PJ, Antao T, Chang JT, Chapman BA, Cox CJ, Dalke A, et al. Biopython: freely available Python tools for computational molecular biology and bioinformatics. *Bioinformatics.* 2009;25(11):1422-3.
3. Venkatarajan MS, and Braun W. New quantitative descriptors of amino acids based on multidimensional scaling of a large number of physical-chemical properties. *J Mol Model.* 2001;7(12):445-53.
4. Sahu C, Jain V, Mishra P, and Prasad KN. Clinical and laboratory standards institute versus European committee for antimicrobial susceptibility testing guidelines for interpretation of carbapenem antimicrobial susceptibility results for Escherichia coli in urinary tract infection (UTI). *J Lab Physicians.* 2018;10(3):289-93.
5. Felix L, Mishra B, Khader R, Ganesan N, and Mylonakis E. In Vitro and In Vivo Bactericidal and Antibiofilm Efficacy of Alpha Mangostin Against Staphylococcus aureus Persister Cells. *Front Cell Infect Microbiol.* 2022;12:898794.
6. Kim W, Zou G, Hari TPA, Wilt IK, Zhu W, Galle N, et al. A selective membrane-targeting repurposed antibiotic with activity against persistent methicillin-resistant Staphylococcus aureus. *Proc Natl Acad Sci U S A.* 2019;116(33):16529-34.
7. Kim W, Conery AL, Rajamuthiah R, Fuchs BB, Ausubel FM, and Mylonakis E. Identification of an Antimicrobial Agent Effective against Methicillin-Resistant Staphylococcus aureus Persisters Using a Fluorescence-Based Screening Strategy. *PLoS One.* 2015;10(6):e0127640.
8. Mishra B, Felix L, Basu A, Kollala SS, Chhonker YS, Ganesan N, et al. Design and Evaluation of Short Bovine Lactoferrin-Derived Antimicrobial Peptides against Multidrug-Resistant Enterococcus faecium. *Antibiotics (Basel).* 2022;11(8).
9. Peng J, Mishra B, Khader R, Felix L, and Mylonakis E. Novel Cecropin-4 Derived Peptides against Methicillin-Resistant Staphylococcus aureus. *Antibiotics (Basel).* 2021;10(1).
10. Mishra B, Basu A, Chua RRY, Saravanan R, Tambyah PA, Ho B, et al. Site specific immobilization of a potent antimicrobial peptide onto silicone catheters: evaluation against urinary tract infection pathogens. *J Mater Chem B.* 2014;2(12):1706-16.
11. Mishra B, Lakshmaiah Narayana J, Lushnikova T, Wang X, and Wang G. Low cationicity is important for systemic in vivo efficacy of database-derived peptides against drug-resistant Gram-positive pathogens. *Proc Natl Acad Sci U S A.* 2019;116(27):13517-22.
12. Shen Y, Delaglio F, Cornilescu G, and Bax A. TALOS+: a hybrid method for predicting protein backbone torsion angles from NMR chemical shifts. *J Biomol NMR.* 2009;44(4):213-23.
13. Guntert P. Automated NMR structure calculation with CYANA. *Methods Mol Biol.* 2004;278:353-78.
14. Jumper J, Evans R, Pritzel A, Green T, Figurnov M, Ronneberger O, et al. Highly accurate protein structure prediction with AlphaFold. *Nature.* 2021;596(7873):583-9.
15. Kumar A, Mishra B, Konar AD, Mylonakis E, and Basu A. Molecular Dynamics Simulations Help Determine the Molecular Mechanisms of Lasioglossin-III and Its Variant Peptides' Membrane Interfacial Interactions. *J Phys Chem B.* 2024;128(25):6049-58.
16. Jo S, Kim T, Iyer VG, and Im W. CHARMM-GUI: a web-based graphical user interface for CHARMM. *J Comput Chem.* 2008;29(11):1859-65.
17. Humphrey W, Dalke A, and Schulten K. VMD: visual molecular dynamics. *J Mol Graph.* 1996;14(1):33-8, 27-8.

18. Kim SM, Zou G, Kim H, Kang M, Ahn S, Heo HY, et al. Antimicrobial activity of the membrane-active compound nTZDpa is enhanced at low pH. *Biomed Pharmacother.* 2022;150:112977.
19. Sherman MB, Guenther R, Reade R, Rochon D, Sit T, and Smith TJ. Near-Atomic-Resolution Cryo-Electron Microscopy Structures of Cucumber Leaf Spot Virus and Red Clover Necrotic Mosaic Virus: Evolutionary Divergence at the Icosahedral Three-Fold Axes. *J Virol.* 2020;94(2).
20. Mishra B, Leishangthem GD, Gill K, Singh AK, Das S, Singh K, et al. A novel antimicrobial peptide derived from modified N-terminal domain of bovine lactoferrin: design, synthesis, activity against multidrug-resistant bacteria and Candida. *Biochim Biophys Acta.* 2013;1828(2):677-86.
21. Fey PD, Endres JL, Yajjala VK, Widhelm TJ, Boissy RJ, Bose JL, and Bayles KW. A genetic resource for rapid and comprehensive phenotype screening of nonessential Staphylococcus aureus genes. *mBio.* 2013;4(1):e00537-12.
22. https://www.novogene.com/us-en/technology/platforms/?_gl=1*1d56su*_up*MQ..&gclid=Cj0KCQjwxeyxBhC7ARIsAC7dS38sDzS-boZzCboRHC_8bR7ie2Q6EnZs8BEPl-uGN_4Oucq0WYyp6mgaAjuFEALw_wcB.
23. Oscorbin IP, and Filipenko ML. M-MuLV reverse transcriptase: Selected properties and improved mutants. *Comput Struct Biotechnol J.* 2021;19:6315-27.
24. Nimmakayala RK, Leon F, Rachagani S, Rauth S, Nallasamy P, Marimuthu S, et al. Metabolic programming of distinct cancer stem cells promotes metastasis of pancreatic ductal adenocarcinoma. *Oncogene.* 2021;40(1):215-31.
25. de Breij A, Riool M, Cordfunke RA, Malanovic N, de Boer L, Koning RI, et al. The antimicrobial peptide SAAP-148 combats drug-resistant bacteria and biofilms. *Sci Transl Med.* 2018;10(423).
26. . https://www.multiplex-cytokine-analysis.com/.
